# Homogenisation of dynamical optimal transport on periodic graphs

**DOI:** 10.1007/s00526-023-02472-z

**Published:** 2023-04-28

**Authors:** Peter Gladbach, Eva Kopfer, Jan Maas, Lorenzo Portinale

**Affiliations:** 1 Institut für Angewandte Mathematik, Universität Bonn, Endenicher Allee 60, 53115 Bonn, Germany; 2 Institute of Science and Technology Austria (IST Austria), Am Campus 1, 3400 Klosterneuburg, Austria

**Keywords:** Primary: 49Q22 Secondary: 49M25, 49J45, 65K10, 74Q10

## Abstract

This paper deals with the large-scale behaviour of dynamical optimal transport on $\mathbb {Z}^d$-periodic graphs with general lower semicontinuous and convex energy densities. Our main contribution is a homogenisation result that describes the effective behaviour of the discrete problems in terms of a continuous optimal transport problem. The effective energy density can be explicitly expressed in terms of a cell formula, which is a finite-dimensional convex programming problem that depends non-trivially on the local geometry of the discrete graph and the discrete energy density. Our homogenisation result is derived from a $\Gamma$-convergence result for action functionals on curves of measures, which we prove under very mild growth conditions on the energy density. We investigate the cell formula in several cases of interest, including finite-volume discretisations of the Wasserstein distance, where non-trivial limiting behaviour occurs.

## Introduction

In the past decades there has been intense research activity in the field of optimal transport, both in pure mathematics and in applied areas [35, 39, 41, 42]. In continuous settings, a central result in the field is the *Benamou–Brenier formula* [6], which establishes the equivalence of static and dynamical optimal transport. It asserts that the classical Monge–Kantorovich problem, in which a cost functional is minimised over couplings of given probability measures $\mu _0$ and $\mu _1$, is equivalent to a dynamical transport problem, in which an energy functional is minimised over all solutions to the continuity equation connecting $\mu _0$ and $\mu _1$.

In discrete settings, the equivalence between static and dynamical optimal transport breaks down, and it turns out that the dynamical formulation [11, 30, 32] is essential in applications to evolution equations, discrete Ricci curvature, and functional inequalities [15–20, 33]. Therefore, it is an important problem to analyse the discrete-to-continuum limit of dynamical optimal transport in various setting.

This limit passage turns out to be highly nontrivial. In fact, seemingly natural discretisations of the Benamou–Brenier formula do not necessarily converge to the expected limit, even in one-dimensional settings [25]. The main result in [26] asserts that, for a sequence of meshes on a bounded convex domain in ${\mathbb {R}}^d$, an isotropy condition on the meshes is required to obtain the convergence of the discrete dynamical transport distances to ${\mathbb {W}}_2$. This is in sharp contrast to the scaling behaviour of the corresponding gradient flow dynamics, for which no additional symmetry on the meshes is required to ensure the convergence of discretised evolution equations to the expected continuous limit [12, 21].

The goal of this paper is to investigate the large-scale behaviour of dynamical optimal transport on graphs with a $\mathbb {Z}^d$-periodic structure. Our main contribution is a homogenisation result that describes the effective behaviour of the discrete problems in terms of a continuous optimal transport problem, in which the effective energy density depends non-trivially on the geometry of the discrete graph and the discrete transport costs.

### Main results

We give here an informal presentation of the main results of this paper, ignoring several technicalities for the sake of readability. Precise formulations and a more general setting can be found from Sect. 2 onwards.

#### Dynamical optimal transport in the continuous setting

For $1 \le p < \infty$, let $\mathbb {W}_p$ be the Wasserstein–Kantorovich–Rubinstein distance between probability measures on a metric space $(X,\textsf{d})$: for $\mu ^0, \mu ^1 \in \mathcal {P}(X)$,

$$\begin{aligned} \mathbb {W}_p(\mu ^0, \mu ^1) := \inf _{ \gamma \in \Gamma (\mu ^0,\mu ^1) } \bigg \{ \int _{X \times X} \textsf{d}(x,y)^p \, \textrm{d}\gamma (x,y) \bigg \}^{1/p}, \end{aligned}$$

where $\Gamma (\mu ^0,\mu ^1)$ denotes the set of couplings of $\mu ^0$ and $\mu ^1$, i.e., all measures $\gamma \in \mathcal {P}(X \times X)$ with marginals $\mu ^0$ and $\mu ^1$. On the torus $\mathbb {T}^d$ (or more generally, on Riemannian manifolds), the Benamou–Brenier formula [3, 6] provides an equivalent dynamical formulation for $p > 1$, namely

1.1 $$\begin{aligned} \mathbb {W}_p(\mu ^0, \mu ^1) = \inf _{(\rho , j) } \bigg \{ \int _0^1 \int _{\mathbb {T}^d} \frac{|j_t(x)|^p}{\rho _t^{p-1}(x)} \, \textrm{d}x \, \textrm{d}t \bigg \}^{1/p}, \end{aligned}$$

where the infimum runs over all solutions $(\rho , j)$ to the continuity equation $\partial _t \rho + \nabla \cdot j = 0$ with boundary conditions $\rho _0(x) \, \textrm{d}x = \mu ^0(\textrm{d}x)$ and $\rho _1(x) \, \textrm{d}x = \mu ^1(\textrm{d}x)$.

In this paper we consider general lower semicontinuous and convex energy densities $f: \mathbb {R}_+ \times \mathbb {R}^d \rightarrow \mathbb {R}\cup \{ + \infty \}$ under suitable (super-)linear growth conditions. (The Benamou–Brenier formula above corresponds to the special case $f(\rho ,j) = \frac{|j|^p}{\rho ^{p-1}}$). For sufficiently regular curves of measures ${\varvec{\mu }}: (0,1) \rightarrow \mathcal {M}_+(\mathbb {T}^d)$, we consider the continuous action

1.2 $$\begin{aligned} \mathbb {A}({\varvec{\mu }}) := \inf _{{\varvec{\nu }}} \bigg \{ \int _0^1 \int _{\mathbb {T}^d} f \bigg ( \frac{ \textrm{d}\mu _t}{\textrm{d}\mathscr {L}^d}, \frac{ \textrm{d}\nu _t}{\textrm{d}\mathscr {L}^d} \bigg ) \, \textrm{d}x \, \textrm{d}t \ : \ ({\varvec{\mu }}, {\varvec{\nu }}) \in \mathbb{C}\mathbb{E}\bigg \}. \end{aligned}$$

Here, the infimum runs over all time-dependent vector-valued measures ${\varvec{\nu }}: (0,1) \rightarrow \mathcal {M}^d(\mathbb {T}^d)$ satisfying the continuity equation $(\mathbb{C}\mathbb{E})$ $\partial _t \mu _t + \nabla \cdot \nu _t = 0$ in the sense of distributions.

#### Dynamical optimal transport in the discrete setting

A natural discrete counterpart to (1.2) can be defined on finite (undirected) graphs $(\mathcal {X}, \mathcal {E})$. For each edge $(x,y) \in \mathcal {E}$ we fix a lower semicontinuous and convex energy density^1^$F_{xy}: \mathbb {R}_+ \times \mathbb {R}_+ \times \mathbb {R}\rightarrow \mathbb {R}_+$. For sufficiently regular curves ${\pmb {m}}: (0,1) \rightarrow \mathcal {M}_+(\mathcal {X})$ we then consider the discrete action

1.3 $$\begin{aligned} \mathcal {A}({\pmb {m}}) := \inf _{\pmb {J}} \bigg \{ \int _0^1 \sum _{(x,y) \in \mathcal {E}} F_{xy} \big ( m_t(x), m_t(y), J_t(x,y) \big ) \, \textrm{d}t \ : \ ({\pmb {m}}, \pmb {J}) \in \mathcal{C}\mathcal{E}\bigg \}. \end{aligned}$$

Here, the infimum runs over all time-dependent “discrete vector fields”, i.e., all anti-symmetric functions $\pmb {J}: (0,1) \rightarrow \mathbb {R}^\mathcal {E}$ satisfying the discrete continuity equation $(\mathcal{C}\mathcal{E})$ $\partial _t m_t(x) + {{\,\mathrm{\text {\textsf{div}}}\,}}J_t(x) = 0$ for all $x \in \mathcal {X}$, where ${{\,\mathrm{\text {\textsf{div}}}\,}}J_t(x):= \sum _{y: (x,y) \in \mathcal {E}} J_t(x,y)$ denotes the discrete divergence. Variational problems of the form (1.3) arise naturally in the formulation of jump processes as generalised gradient flows [37].

#### Dynamical optimal transport on $\mathbb {Z}^d$-periodic graphs

**Fig. 1:**
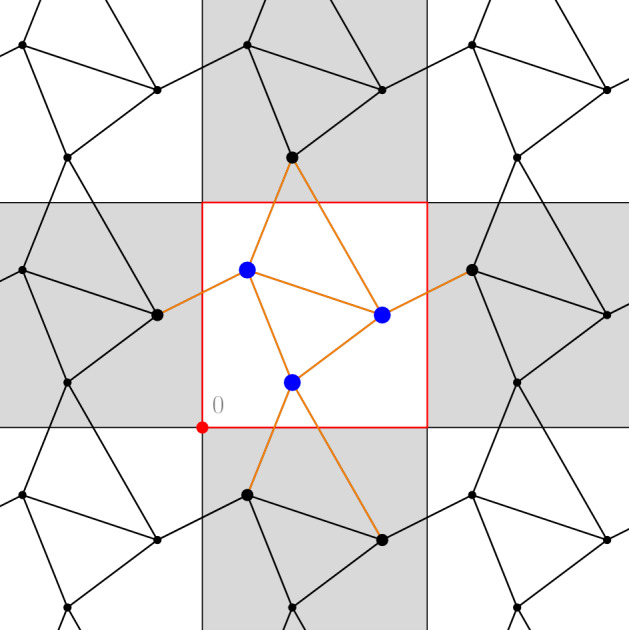
A fragment of a $\mathbb {Z}^d$-periodic graph $(\mathcal {X}, \mathcal {E})$. The unit cube $Q:= [0,1)^d \subset \mathbb {R}^d$ is shown in red. In blue and in orange, respectively, $\mathcal {X}^Q$ and $\mathcal {E}^Q$ (color figure online)

In this work we fix a $\mathbb {Z}^d$-periodic graph $(\mathcal {X},\mathcal {E})$ embedded in $\mathbb {R}^d$, as in Fig. 1. For sufficiently small $\varepsilon > 0$ with $1/\varepsilon \in \mathbb {N}$, we then consider the finite graph $(\mathcal {X}_\varepsilon , \mathcal {E}_\varepsilon )$ obtained by scaling $(\mathcal {X}, \mathcal {E})$ by a factor $\varepsilon$, and wrapping the resulting graph around the torus, so that the resulting graph is embedded in $\mathbb {T}^d$. We are interested in the behaviour of the rescaled discrete action, defined for curves ${\pmb {m}}: (0,1) \rightarrow \mathcal {M}_+(\mathcal {X}_\varepsilon )$ by

1.4 $$\begin{aligned} \mathcal {A}_\varepsilon ({\pmb {m}}) := \inf _{\pmb {J}} \bigg \{ \int _0^1 \sum _{(x,y) \in \mathcal {E}_\varepsilon } \varepsilon ^d F_{xy} \bigg ( \frac{m_t(x)}{\varepsilon ^d}, \frac{m_t(y)}{\varepsilon ^d}, \frac{J_t(x,y)}{\varepsilon ^{d-1}} \bigg ) \, \textrm{d}t \ : \ ({\pmb {m}}, \pmb {J}) \in \mathcal{C}\mathcal{E}_\varepsilon \bigg \}. \end{aligned}$$

As above, the infimum runs over all time-dependent “discrete vector fields” $\pmb {J}: (0,1) \rightarrow \mathbb {R}^{\mathcal {E}_\varepsilon }$ satisfying the discrete continuity equation $(\mathcal{C}\mathcal{E}_\varepsilon )$ on the rescaled graph $(\mathcal {X}_\varepsilon , \mathcal {E}_\varepsilon )$.

#### Convergence of the action

One of our main results (Theorem 5.1) asserts that, as $\varepsilon \rightarrow 0$, the action functionals $\mathcal {A}_\varepsilon$ converge to a limiting functional $\mathbb {A}= \mathbb {A}_\textrm{hom}$ of the form (1.2), with an effective energy density $f = f_\textrm{hom}$ which depends non-trivially on the geometry of the graph $(\mathcal {X},\mathcal {E})$ and the discrete energy densities $F_{xy}$. We only require a very mild linear growth condition on the energy densities $F_{xy}$:

*As* $\varepsilon \rightarrow 0$, *the functionals* $\mathcal {A}_\varepsilon$ $\Gamma$-*converge to* $\mathbb {A}_{\textrm{hom}}$ *in the weak (and vague) topology of* $\mathcal {M}_+\big ((0,1) \times \mathbb {T}^d\big )$.

The precise formulation of this result involves an extension of $\mathbb {A}_\textrm{hom}$ to measures on $(0,1) \times \mathbb {T}^d$; see Sect. 3 below.

Let us now explain the form of the effective energy density $f_\textrm{hom}$, which is given by a cell formula. For given $\rho \ge 0$ and $j \in \mathbb {R}^d$, $f_\textrm{hom}(\rho , j)$ is obtained by minimising the discrete energy per unit cube among all periodic mass distributions $m: \mathcal {X}\rightarrow \mathbb {R}_+$ representing $\rho$, and all periodic divergence-free discrete vector fields $J: \mathcal {E}\rightarrow \mathbb {R}$ representing *j* in the following sense. Set $\mathcal {X}^Q:= \mathcal {X}\cap [0,1)^d$ and $\mathcal {E}^Q:= \big \{ (x,y) \in \mathcal {E}\ : \ x \in \mathcal {X}^Q \big \}$. Then $f_\textrm{hom}:\mathbb {R}_+ \times \mathbb {R}^d \rightarrow \mathbb {R}_+$ is given by

1.5 $$\begin{aligned} f_\textrm{hom}(\rho ,j) := \inf _{m,J} \bigg \{ \sum _{(x,y) \in \mathcal {E}^Q} F_{xy} \big ( m(x), m(y), J(x,y) \big ) \ : \ (m,J) \in {{\,\mathrm{{\textsf{Rep}}}\,}}(\rho ,j) \bigg \}, \end{aligned}$$

where the set of representatives ${{\,\mathrm{{\textsf{Rep}}}\,}}(\rho ,j)$ consists of all $\mathbb {Z}^d$-periodic functions $m: \mathcal {X}\rightarrow \mathbb {R}_+$ and all $\mathbb {Z}^d$-periodic discrete vector fields satisfying

1.6 $$\begin{aligned} \sum _{x \in \mathcal {X}^Q} m(x) = \rho \, , \quad {{\,\mathrm{\text {\textsf{div}}}\,}}J = 0 \, , \quad \text { and }\quad {{\,\mathrm{\textsf{Eff}}\,}}(J) := \frac{1}{2} \sum _{(x,y) \in \mathcal {E}^Q} J(x,y) (y-x) = j. \end{aligned}$$

#### Boundary value problems

Our second main result deals with the corresponding boundary value problems, which arise by minimising the action functional among all curves with given boundary conditions, as in the Benamou–Brenier formula (1.1). We define

$$\begin{aligned} \mathcal{M}\mathcal{A}_\varepsilon (m^0,m^1)&:= \inf _{{\pmb {m}}} \big \{ \mathcal {A}_\varepsilon ({\pmb {m}}) \ : \ m_0 = m^0, \; m_1 = m^1 \big \}{} & {} \text {for } m^0, m^1 \in \mathcal {P}(\mathcal {X}_\varepsilon ), \\ \mathbb{M}\mathbb{A}_\textrm{hom}(\mu ^0,\mu ^1)&:= \inf _{{\varvec{\mu }}} \big \{ \mathbb {A}_\textrm{hom}({\varvec{\mu }}) \ : \ \mu _0 = \mu ^0, \; \mu _1 = \mu ^1 \big \}{} & {} \text {for } \mu ^0, \mu ^1 \in \mathcal {P}(\mathbb {T}^d). \end{aligned}$$

We then obtain the following result (Theorem 5.10):

*As* $\varepsilon \rightarrow 0$, *the minimal actions* $\mathcal{M}\mathcal{A}_\varepsilon$ $\Gamma$-*converge to* $\mathbb{M}\mathbb{A}_{\textrm{hom}}$

*in the weak topology of* $\mathcal {M}_+(\mathbb {T}^d) \times \mathcal {M}_+(\mathbb {T}^d)$.

This result is proved under a superlinear growth condition on the discrete energy densities, which holds for discretisations of the Wasserstein distance $\mathbb {W}_p$ for $p > 1$.

A special case of interest is the case where $\mathcal{M}\mathcal{A}_\varepsilon$ is a Riemannian transport distance associated to a gradient flow structure for Markov chains as in [30, 32]. In this situation, we show that the discrete transport distances converge to a 2-Wasserstein distance on the torus (Corollary 5.3). Interestingly, the underlying distance is induced by a Finsler metric, which is not necessarily Riemannian.

We also investigate transport distances with nonlinear mobility [13, 29] and their finite-volume discretisations on the torus $\mathbb {T}^d$. In the spirit of [26], we give a geometric characterisation of finite-volume meshes for which the discretised transport distances converge to the expected limit.

#### Compactness

The results for boundary value problems are obtained by combining our first main result with a compactness result for sequence of measures with bounded action, which is of independent interest. We obtain two results of this type.

In the first compactness result (Theorem 5.4) we assume at least linear growth of the discrete energies $F_{xy}$ at infinity. Under this condition we prove compactness in the space $\textrm{BV}_{\textrm{KR}}\big ((0,1); \mathcal {M}_+(\mathbb {T}^d) \big )$, which consists of curves of bounded variation, with respect to the Kantorovich–Rubinstein ($\textrm{KR}$) norm on the space of measures. The convergence holds for almost every $t\in (0,1)$.

In the second compactness result (Theorem 5.9), which is used in the analysis of the boundary value problems, we assume a stronger condition of at least superlinear growth on the energy densities $F_{xy}$. We then obtain compactness in the space $W_\textrm{KR}^{1,1}\big ((0,1); \mathcal {M}_+(\mathbb {T}^d)\big )$, which consists of absolutely continuous curves with respect to the $\textrm{KR}$-norm. The convergence is uniform for $t \in (0,1)$. We refer to the “Appendix” for precise definitions of these spaces.

#### Related works

For a classical reference to the study of flows on networks, we refer to Ford and Fulkerson [22].

Many works are devoted to discretisations of continuous energy functionals in the framework of Sobolev and BV spaces, e.g., [1, 4, 5, 36]. Cell formulas appear in various discrete and continuous variational homogenisation problems; see, e.g., [4, 7, 9, 27, 31].

The large scale behaviour of optimal transport on random point clouds has been studied by Garcia–Trillos, who proved convergence to the Wasserstein distance [23].

### Organisation of the paper

Sects. 2 and 3 contain the necessary definitions as well as the assumptions we use throughout the article in the discrete and continuous settings. Section 4 deals with the definition of the homogenised action functional. In Sect. 5 we present the rigorous statements of our main results, including the $\Gamma$-convergence of the discrete energies to the effective homogenised limit and the compactness theorems for curves of bounded discrete energies. The proof of our main results can be found in Sect. 6 (compactness and convergence of the boundary value problems) and Sects. 7 and 8 ($\Gamma$-convergence of $\mathcal {A}_\varepsilon$). Finally, in Sect. 9, we discuss several examples and apply our results to some common finite-volume and finite-difference discretisations.

### Sketch of the proof of Theorem 5.1

In the last part of this section, we sketch the proof of our main result on the convergence of $\mathcal {A}_\varepsilon$ to the homogenised limit (Theorem 5.1). Crucial tools to show both the lower bound and the upper bound in Theorem 5.1 are regularisation procedures for solutions to the continuity equation, both at the discrete and at the continuous level.

In this section, we use the informal notation $\lessapprox$ and $> rapprox$ to mean that the corresponding inequality holds up to a small error in $\varepsilon >0$, e.g., $A_\varepsilon \lessapprox B_\varepsilon$ means that $A_\varepsilon \le B_\varepsilon + o_\varepsilon (1)$ where $o_\varepsilon (1) \rightarrow 0$ as $\varepsilon \rightarrow 0$.

For $\varepsilon > 0$ and $z \in \mathbb {Z}^d$ (or more generally, for $z \in \mathbb {R}^d$), we set $Q_\varepsilon ^z:= \varepsilon z + [0, \varepsilon )^d \subseteq \mathbb {T}^d$. For $x \in \mathcal {X}_\varepsilon \subset \mathbb {T}^d$, we denote by $x_\textsf{z}$ the unique element of $\mathbb {Z}_\varepsilon ^d$ satisfying $x \in Q_\varepsilon ^{x_\textsf{z}}$. Note that $\{Q_\varepsilon ^z \ : \ z \in \mathbb {Z}_\varepsilon ^d\}$ defines a partition of $\mathbb {T}^d$.

To compare discrete and continuous objects, we consider embeddings of probability measures $m \in \mathcal {P}(\mathcal {X}_\varepsilon )$ and anti-symmetric functions $J: \mathcal {E}_\varepsilon \rightarrow \mathbb {R}$ defined by

$$\begin{aligned} \iota _\varepsilon m&:= \varepsilon ^{-d} \sum _{x \in \mathcal {X}_\varepsilon } m(x) \mathscr {L}^d|_{Q_\varepsilon ^{x_\textsf{z}}} \ \in \mathcal {P}(\mathbb {T}^d) , \\ \iota _\varepsilon J&:= \varepsilon ^{-d+1} \sum _{(x,y) \in \mathcal {E}_\varepsilon } \frac{J(x,y)}{2} \bigg (\int _0^1 \mathscr {L}^d|_{ Q_\varepsilon ^{(1-s)x_\textsf{z}+ s y_\textsf{z}}} \, \textrm{d}s\bigg ) (y_\textsf{z}- x_\textsf{z}) \ \in \mathcal {M}^d(\mathbb {T}^d). \end{aligned}$$

These embeddings preserve the continuity equation in the following sense: if $({\pmb {m}}, \pmb {J}) \in \mathcal{C}\mathcal{E}_\varepsilon$, then $(\iota _\varepsilon {\pmb {m}}, \iota _\varepsilon \pmb {J}) \in \mathbb{C}\mathbb{E}$.

We also use the notation $\mathcal {F}_\varepsilon (m,J):= \sum _{(x,y) \in \mathcal {E}_\varepsilon } \varepsilon ^d F_{xy} \Big ( \frac{m(x)}{\varepsilon ^d}, \frac{m(y)}{\varepsilon ^d}, \frac{J(x,y)}{\varepsilon ^{d-1}} \Big )$.

*Sketch of the liminf inequality*. For $\varepsilon > 0$ with $\frac{1}{\varepsilon } \in \mathbb {N}$, consider the curve $(m_t^\varepsilon )_{t \in (0,1)} \subseteq \mathcal {M}_+(\mathcal {X}_\varepsilon )$ and let ${\pmb {m}}^\varepsilon \in \mathcal {M}_+\big ((0,1) \times \mathcal {X}_\varepsilon \big )$ be the corresponding measure on space-time defined by ${\pmb {m}}^\varepsilon (\textrm{d}x, \textrm{d}t) = m_t^\varepsilon (\textrm{d}x) \, \textrm{d}t$. Suppose that $\iota _\varepsilon {\pmb {m}}^\varepsilon \rightarrow {\varvec{\mu }}$ vaguely in $\mathcal {M}_+\big ((0,1) \times \mathbb {T}^d\big )$ as $\varepsilon \rightarrow 0$. The goal is to show the *liminf inequality*

1.7 $$\begin{aligned} \liminf _{\varepsilon \rightarrow 0} \mathcal {A}_\varepsilon ({\pmb {m}}^\varepsilon ) \ge \mathbb {A}_{\textrm{hom}}({\varvec{\mu }}). \end{aligned}$$

Without loss of generality we assume that $\mathcal {A}_\varepsilon ({\pmb {m}}^\varepsilon ) = \mathcal {A}_\varepsilon ({\pmb {m}}^\varepsilon , \pmb {J}^\varepsilon ) \le C < \infty$ for every $\varepsilon >0$, for some sequence of vector fields $\pmb {J}^\varepsilon$ such that $({\pmb {m}}^\varepsilon , \pmb {J}^\varepsilon ) \in \mathcal{C}\mathcal{E}_\varepsilon$. As we will see in (4.11), the embedded solutions to the continuity equation $(\iota _\varepsilon {\pmb {m}}^\varepsilon , \iota _\varepsilon \pmb {J}^\varepsilon ) \in \mathbb{C}\mathbb{E}$ define curves of measures with densities with respect to $\mathscr {L}^d$ on $\mathbb {T}^d$ of the form

$$\begin{aligned} \rho _t(u) = \varepsilon ^{-d} \sum _{\begin{array}{c} x \in \mathcal {X}_\varepsilon \\ x_\textsf{z}= \bar{z} \end{array}} m_t^\varepsilon (x) \quad \text { and }\quad j_t(u) = \frac{1}{2\varepsilon ^{d-1}} \sum _{\begin{array}{c} (x,y) \in \mathcal {E}_\varepsilon \\ x_\textsf{z}= \bar{z} \end{array}} J_{t,u}^\varepsilon (x,y) \big ( y_\textsf{z}- x_\textsf{z}\big ) \end{aligned}$$

for every $u \in Q_\varepsilon ^{\bar{z}} \subset \mathbb {T}^d$. Here, $J_{t,u}^\varepsilon \in \mathbb {R}^{\mathcal {E}_\varepsilon }$ is a convex combination of the functions $\big \{ J_t^\varepsilon \big ( \cdot -\varepsilon z \big ) \,: \, z \in \mathbb {Z}_\varepsilon ^d, \, |z|_\infty \le R_0 + 1 \big \}$.

As we will estimate the discrete energies at any time $t \in (0,1)$, for simplicity we drop the time dependence and write $\rho = \rho _t$, $j= j_t$, $m^\varepsilon =m_t^\varepsilon$, $J^\varepsilon =J_t^\varepsilon$, $J_u^\varepsilon = J_{t,u}^\varepsilon$. A crucial step is to construct, for every $u \in Q_\varepsilon ^{\bar{z}}$, a representative

1.8 $$\begin{aligned} \bigg ( \frac{\widehat{m}_u}{\varepsilon ^d}, \frac{\widehat{J}_u}{\varepsilon ^{d-1}} \bigg ) \in {{\,\mathrm{{\textsf{Rep}}}\,}}\big ( \rho (u), j(u) \big ) \end{aligned}$$

which is approximately equal to the values of $(m^\varepsilon , J^\varepsilon )$ close to $\mathcal {X}\cap \{ x_\textsf{z}= \bar{z} \}$. The lower bound (1.7) would then follow by time-integration of the static estimate

1.9 $$\begin{aligned} \begin{aligned} \mathcal {F}_\varepsilon (m^\varepsilon , J^\varepsilon )& > rapprox \sum _{\bar{z} \in \mathbb {Z}_\varepsilon ^d} \sum _{(x,y) \in \mathcal {E}^Q} \varepsilon ^d F_{xy} \bigg ( \frac{\widehat{m}_{\varepsilon \bar{z}}(x)}{\varepsilon ^d}, \frac{\widehat{m}_{\varepsilon \bar{z}}(y)}{\varepsilon ^d}, \frac{\widehat{J}_{\varepsilon \bar{z}}(x,y)}{\varepsilon ^{d-1}} \bigg ) \\ {}& > rapprox \int _{\mathbb {T}^d} f_\textrm{hom}\big ( \rho (u), j(u) \big ) \, \textrm{d}u = \mathbb {F}_\textrm{hom}(\iota _\varepsilon m^\varepsilon , \iota _\varepsilon J^\varepsilon ), \end{aligned} \end{aligned}$$

together with the lower semicontinuity of $\mathbb {A}_\textrm{hom}$. In the last inequality we used the definition of the homogenised density $f_\textrm{hom}\big (\rho (u),j(u)\big )$, which corresponds to the minimal microscopic cost with total mass $\rho (u)$ and flux *j*(*u*).

To find the sought representatives in (1.8), it may seem natural to define $\widehat{m}_u \in \mathbb {R}_+^{\mathcal {X}}$ and $\widetilde{J}_u \in \mathbb {R}_a^{\mathcal {E}}$ by taking the values of *m* and $J_u$ in the $\varepsilon$-cube at $\bar{z}$, and insert these values at every cube in $(\mathcal {X}, \mathcal {E})$, so that the result is $\mathbb {Z}^d$-periodic. Precisely:

$$\begin{aligned} \widehat{m}_u(x) := m(\varepsilon \bar{x}) , \quad \widetilde{J}_u ( x,y ) := J_u \big ( \varepsilon \bar{x}, \varepsilon (y - x_\textsf{z}+ \bar{z} ) \big ) , \quad \text {for } ( x,y ) \in \mathcal {E}, \end{aligned}$$

where $\bar{x}:= x - x_\textsf{z}+ \bar{z}$. This would ensure that $\varepsilon ^{-d} \widehat{m}_u \in {{\,\mathrm{{\textsf{Rep}}}\,}}\big ( \rho (u) \big )$. Unfortunately, this construction produces a vector field $\varepsilon ^{-(d-1)}\widetilde{J}_u$ which does not in general belong to ${{\,\mathrm{{\textsf{Rep}}}\,}}\big (j(u)\big )$: indeed, while $\widetilde{J}_u$ has the desired effective flux (i.e., ${{\,\mathrm{\textsf{Eff}}\,}}(\varepsilon ^{-(d-1)}\widetilde{J}_u) = j(u)$, as given in (1.6)), it is not in general divergence-free.

To deal with this complication, we introduce a *corrector field* $\bar{J}_u$ associated to $\widetilde{J}_u$, i.e., an anti-symmetric and $\mathbb {Z}^d$-periodic function $\bar{J}_u: \mathcal {E}\rightarrow \mathbb {R}$ satisfying

1.10 $$\begin{aligned} {{\,\mathrm{\text {\textsf{div}}}\,}}\bar{J}_u = -{{\,\mathrm{\text {\textsf{div}}}\,}}\widetilde{J}_u, \quad {{\,\mathrm{\textsf{Eff}}\,}}(\bar{J}_u ) = 0, \quad \text { and }\quad \big \Vert \bar{J}_u \big \Vert _{\ell ^\infty (\mathcal {E}^Q)} \le \tfrac{1}{2} \big \Vert {{\,\mathrm{\text {\textsf{div}}}\,}}\widetilde{J}_u \big \Vert _{\ell ^1(\mathcal {X}^Q)} , \end{aligned}$$

whose existence we prove in Lemma 7.3.

It is clear that if we set $\widehat{J}_u:= \widetilde{J}_u + \bar{J}_u$ by construction we have ${{\,\mathrm{\text {\textsf{div}}}\,}}\widehat{J}_u = 0$ and ${{\,\mathrm{\textsf{Eff}}\,}}\big (\varepsilon ^{-(d-1)}\widehat{J}_u\big ) = j(u)$, thus

$$\begin{aligned} \frac{\widehat{J}_u}{\varepsilon ^{d-1}} := \frac{\widetilde{J}_u + \bar{J}_u}{{\varepsilon ^{d-1}}} \in {{\,\mathrm{{\textsf{Rep}}}\,}}\big (j_u \big ). \end{aligned}$$

To carry out this program and prove a lower bound of the form (1.9), we need to quantify the error we perform passing from $(m^\varepsilon , J^\varepsilon )$ to $\big \{ (\widehat{m}_u, \widehat{J}_u) \ : \ u \in \mathbb {T}^d\big \}$. It is evident by construction and from (1.10) that spatial and time regularity of $(m^\varepsilon , J^\varepsilon )$ are crucial to this purpose. For example, an $\ell ^\infty$-bound on the time derivative of the form $\Vert \partial _t m_t^\varepsilon \Vert _\infty \le C \varepsilon ^d$ (or, in other words, a Lipschitz bound in time for $\rho _t$) together with $({\pmb {m}}^\varepsilon , \pmb {J}^\varepsilon ) \in \mathcal{C}\mathcal{E}_\varepsilon$ would imply a control on ${{\,\mathrm{\text {\textsf{div}}}\,}}J$ and thus a control of the error in (1.10) of the form $\Vert \varepsilon ^{1-d}\bar{J}_u \Vert _\infty \le C \varepsilon$.

This is why the key first step in our proof is a regularisation procedure at the discrete level: for any given sequence of curves $\big \{ ({\pmb {m}}^\varepsilon , \pmb {J}^\varepsilon ) \in \mathcal{C}\mathcal{E}_\varepsilon \ : \ \varepsilon >0 \big \}$ of (uniformly) bounded action $\mathcal {A}_\varepsilon$, we can exihibit another sequence $\big \{ (\widetilde{{\pmb {m}}{}}^\varepsilon , \widetilde{\pmb {J}{}}^\varepsilon ) \in \mathcal{C}\mathcal{E}_\varepsilon \ : \ \varepsilon >0 \big \}$, quantitatively close as measures and in action $\mathcal {A}_\varepsilon$ to the first one, which enjoy good Lipschitz and $l^\infty$ properties and for which the above explained program can be carried out.

This result is the content of Proposition 7.1 and it is based on a three-fold regularisation, that is in energy, in time, and in space (see Sect. 7.1).

*Sketch of the limsup inequality*. Fix $({\varvec{\mu }},{\varvec{\nu }}) \in \mathbb{C}\mathbb{E}$. The goal is to find ${\pmb {m}}^\varepsilon \in \mathcal {M}_+((0,1) \times \mathcal {X}_\varepsilon )$ such that $\iota _\varepsilon {\pmb {m}}^\varepsilon \rightarrow {\varvec{\mu }}$ weakly in $\mathcal {M}_+((0,1) \times \mathbb {T}^d)$ and

1.11 $$\begin{aligned} \limsup _{\varepsilon \rightarrow 0} \mathcal {A}_\varepsilon ({\pmb {m}}^\varepsilon ) \le \mathbb {A}_{\textrm{hom}}({\varvec{\mu }}, {\varvec{\nu }}). \end{aligned}$$

As in the the proof of the liminf inequality, the first step is a regularisation procedure, this time at the continuous level (Proposition 8.26). Thanks to this approximation result, we can assume without loss of generality that

1.12 $$\begin{aligned} \mathbb {A}_\textrm{hom}({\varvec{\mu }}, {\varvec{\nu }}) < \infty \quad \text { and }\quad \Big \{ \big (\rho _t(x),j_t(x)\big ) \ : \ (t,x) \in (0,1) \times \mathbb {T}^d\Big \} \Subset {{\,\mathrm{\textsf{D}}\,}}(f_{\textrm{hom}})^\circ , \end{aligned}$$

where $(\rho _t,j_t)_t$ are the *smooth* densities of $({\varvec{\mu }},{\varvec{\nu }})\in \mathbb{C}\mathbb{E}$ with respect to $\mathscr {L}^{d+1}$ on $(0,1) \times \mathbb {T}^d$, and ${{\,\mathrm{\textsf{D}}\,}}(f_{\textrm{hom}})^\circ$ denotes the interior of the domain of $f_{\textrm{hom}}$ (see “Appendix 1”). The convexity of $f_\textrm{hom}$ ensures its Lipschitz-continuity on every compact set $K \Subset {{\,\mathrm{\textsf{D}}\,}}(f_{\textrm{hom}})^\circ$, hence the assumption (1.12) allows us to assume such regularity for the rest of the proof.

We split the proof of the upper bound into several steps. In short, we first discretise the continuous measures $({\varvec{\mu }},{\varvec{\nu }})$ and identify an *optimal discrete microstructure*, i.e., minimisers of the cell problem described by $f_\textrm{hom}$, on each $\varepsilon$-cube $Q_\varepsilon ^z$, $z \in \mathbb {Z}_\varepsilon ^d$. A key difficulty at this stage is that the optimal selection does not preserve the continuity equation, hence an additional *correction* is needed. For this purpose, we first apply the discrete regularisation result Proposition 7.1 to obtain regular discrete curves and then find suitable *small* correctors that provide discrete competitors for $\mathcal {A}_\varepsilon$, i.e., solutions to $\mathcal{C}\mathcal{E}_\varepsilon$ which are *close* to the optimal selection.

Let us explain these steps in more detail.

*Step 1*: For every $z \in \mathbb {Z}_\varepsilon ^d$, $t \in (0,1)$, and each cube $Q_\varepsilon ^z$ we consider the natural discretisation of $({\varvec{\mu }},{\varvec{\nu }})$, that we denote by $\big ( \textrm{P}_\varepsilon \mu _t(z),\textrm{P}_\varepsilon \nu _t(z) \big )_{t,z} \subset \mathbb {R}_+ \times \mathbb {R}^d$, given by

$$\begin{aligned} \textrm{P}_\varepsilon \mu _t(z):=\mu _t(Q_\varepsilon ^z), \quad \textrm{P}_\varepsilon \nu _t(z):= \left( \int _{\partial Q_\varepsilon ^z\cap \partial Q_\varepsilon ^{z+ e_i}}j_t\cdot e_i\, \textrm{d}\mathcal {H}^{d-1} \right) _{i=1}^d. \end{aligned}$$

An important feature of this construction is that the continuity equation is preserved from $\mathbb {T}^d$ to $\mathbb {Z}_\varepsilon ^d$, in the sense that

$$\begin{aligned} \partial _t \textrm{P}_\varepsilon \mu _t(z) + \sum _{i=1}^d \big ( \textrm{P}_\varepsilon \nu _t(z) - \textrm{P}_\varepsilon \nu _t(z - e_i) \big ) \cdot e_i = 0 \end{aligned}$$

for $$t \in (0,1)$$ and $$z \in \mathbb {Z}_\varepsilon ^d$$.

*Step 2*: We build the associated *optimal discrete microstructure* for the cell problem for each cube $Q_\varepsilon ^z$, meaning we select $({\pmb {m}},\pmb {J}) = \big ( m_t^z, J_t^z \big )_{t\in (0,1), z \in \mathbb {Z}_\varepsilon ^d}$ such that

$$\begin{aligned} \bigg ( \frac{m_t^z}{\varepsilon ^d},\frac{J_t^z}{\varepsilon ^{d-1}} \bigg ) \in {{\,\mathrm{{\textsf{Rep}}}\,}}_o \bigg ( \frac{\textrm{P}_\varepsilon \mu _t(z)}{\varepsilon ^d} , \frac{\textrm{P}_\varepsilon \nu _t(z)}{\varepsilon ^{d-1}} \bigg ) , \end{aligned}$$

where ${{\,\mathrm{{\textsf{Rep}}}\,}}_o$ denotes the set of optimal representatives in the definition of the cell-formula (1.5). Using the smoothness of ${\varvec{\mu }}$ and ${\varvec{\nu }}$, one can in particular show that

1.13 $$\begin{aligned} \sum _{z \in \mathbb {Z}_\varepsilon ^d} \sum _{(x,y) \in \mathcal {E}^Q} \varepsilon ^d F_{xy} \left( \frac{ m_t^z(x)}{\varepsilon ^d}, \frac{ m_t^z(y)}{\varepsilon ^d}, \frac{ J_t^z(x,y)}{\varepsilon ^{d-1}} \right) \lessapprox \mathbb {F}_{\textrm{hom}}(\mu _t, \nu _t). \end{aligned}$$

*Step 3*: The next step is to glue together the microstructures $({\pmb {m}}, \pmb {J})$ defined for every $z \in \mathbb {Z}_\varepsilon ^d$ via a *gluing operator* $\mathcal {G}_\varepsilon$ (Definition 8.4) to produce a global microstructure $(\widehat{{\pmb {m}}{}}^\varepsilon , \widehat{\pmb {J}{}}^\varepsilon ) \in \mathcal {M}_+((0,1) \times \mathcal {X}_\varepsilon ) \times \mathcal {M}((0,1) \times \mathcal {E}_\varepsilon )$. As the gluing operators are mass preserving and $m_t^z \in {{\,\mathrm{{\textsf{Rep}}}\,}}(\textrm{P}_\varepsilon \mu _t(z))$, it is not hard to see that $\iota _\varepsilon \widehat{{\pmb {m}}{}}^\varepsilon \rightarrow {\varvec{\mu }}$ weakly in $\mathcal {M}_+((0,1) \times \mathbb {T}^d)$ as $\varepsilon \rightarrow 0$.

*Step 4*: In contrast to $\textrm{P}_\varepsilon$, the latter operation produces curves $(\widehat{{\pmb {m}}{}}^\varepsilon , \widehat{\pmb {J}{}}^\varepsilon )$ which do not in general solve the discrete continuity equation $\mathcal{C}\mathcal{E}_\varepsilon$. Therefore, we seek to find suitable *corrector vector fields* in order to obtain a discrete solution, and thus a candidate for $\mathcal {A}_\varepsilon (\widehat{{\pmb {m}}{}}^\varepsilon )$. For this purpose we regularise $(\widehat{{\pmb {m}}{}}^\varepsilon , \widehat{\pmb {J}{}}^\varepsilon )$ using Proposition 7.1 below. This yields a regular curve which is close in the sense of measures and in energy to the original one. Note that no discrete regularity is guaranteed for $(\widehat{{\pmb {m}}{}}^\varepsilon , \widehat{\pmb {J}{}}^\varepsilon )$, despite the smoothness assumption on $({\varvec{\mu }},{\varvec{\nu }})$, due to possible singularities of $F_{xy}$.

For the sake of the exposition, we shall discuss the last steps of the proof assuming that $(\widehat{{\pmb {m}}{}}^\varepsilon , \widehat{\pmb {J}{}}^\varepsilon )$ already enjoy the Lipschitz and $\ell ^\infty$–regularity properties ensured by Proposition 7.1.

*Step 5*: For sufficiently regular $(\widehat{{\pmb {m}}{}}^\varepsilon , \widehat{\pmb {J}{}}^\varepsilon )$, we seek a discrete competitor for $\mathcal {A}_\varepsilon (\widehat{{\pmb {m}}{}}^\varepsilon )$ which is close to $(\widehat{{\pmb {m}}{}}^\varepsilon , \widehat{\pmb {J}{}}^\varepsilon )$. As the latter does not necessary belong to $\mathcal{C}\mathcal{E}_\varepsilon$, we find suitable correctors $\pmb {V}^\varepsilon$ such that the corrected curves $(\widehat{{\pmb {m}}{}}^\varepsilon , \widehat{\pmb {J}{}}^\varepsilon + \pmb {V}^\varepsilon )$ belong to $\mathcal{C}\mathcal{E}_\varepsilon$, with $\pmb {V}^\varepsilon$ small in the sense that it satisfies the bound

1.14 $$\begin{aligned} \sup _{t \in (0,1)} \big \Vert \varepsilon ^{1-d} V_t^\varepsilon \big \Vert _{\ell _\infty (\mathcal {E}_\varepsilon )} \le C \varepsilon . \end{aligned}$$

The proof of existence of the corrector $\pmb {V}^\varepsilon$, together with the quantitative bound relies on a localisation argument (Lemma 8.22) and a study of the divergence equation on periodic graphs (Lemma 8.16), performed at the level of each cube $Q_\varepsilon ^z$, for every $z \in \mathbb {Z}_\varepsilon ^d$. The regularity of $(\widehat{{\pmb {m}}{}}^\varepsilon , \widehat{\pmb {J}{}}^\varepsilon )$ is crucial in order to obtain the estimate (1.14).

*Step 6*: The final step consists of estimating the action of the measures defined by ${\pmb {m}}^\varepsilon := \widehat{{\pmb {m}}{}}^\varepsilon \rightarrow {\varvec{\mu }}$ weakly as $\varepsilon \rightarrow 0$, and the vector fields $\pmb {J}^\varepsilon := \widehat{\pmb {J}{}}^\varepsilon + \pmb {V}^\varepsilon$.

Using the regularity assumption on $(\widehat{{\pmb {m}}{}}^\varepsilon , \widehat{\pmb {J}{}}^\varepsilon )$, the smoothness (1.12) of $({\varvec{\mu }},{\varvec{\nu }})$, and the convexity of $f_\textrm{hom}$, together with the bounds (1.13) and (1.14) for the corrector, we obtain

$$\begin{aligned} \mathcal {F}_\varepsilon (m_t^\varepsilon , J_t^\varepsilon )&\lessapprox \mathcal {F}_\varepsilon (\widehat{m}_t^\varepsilon , \widehat{J}_t^\varepsilon ) \lessapprox \sum _{z \in \mathbb {Z}_\varepsilon ^d} \sum _{(x,y) \in \mathcal {E}^Q} \varepsilon ^d F_{xy} \left( \frac{ m_t^z(x)}{\varepsilon ^d}, \frac{ m_t^z(y)}{\varepsilon ^d}, \frac{ J_t^z(x,y)}{\varepsilon ^{d-1}} \right) \\&\lessapprox \mathbb {F}_{\textrm{hom}}(\mu _t, \nu _t). \end{aligned}$$

Using this bound and the fact that $({\pmb {m}}^\varepsilon , \pmb {J}^\varepsilon ) \in \mathcal{C}\mathcal{E}_\varepsilon$, an integration in time yields

$$\begin{aligned} \limsup _{\varepsilon \rightarrow 0} \mathcal {A}_\varepsilon ({\pmb {m}}^\varepsilon ) \le \limsup _{\varepsilon \rightarrow 0} \mathcal {A}_\varepsilon ({\pmb {m}}^\varepsilon , \pmb {J}^\varepsilon ) \le \mathbb {A}_\textrm{hom}({\varvec{\mu }},{\varvec{\nu }}), \end{aligned}$$

which is the sought upper bound (1.11).

## Discrete dynamical optimal transport on $\mathbb {Z}^d$-periodic graphs

This section contains the definition of the optimal transport problem in the discrete periodic setting. In Sect. 2.1 we introduce the basic objects: a $\mathbb {Z}^d$-periodic graph $(\mathcal {X}, \mathcal {E})$ and an admissible cost function *F*. Given a triple $(\mathcal {X}, \mathcal {E}, F)$, we introduce a family of discrete transport actions on rescaled graphs $(\mathcal {X}_\varepsilon , \mathcal {E}_\varepsilon )$ in Sect. 2.2.

### Discrete $\mathbb {Z}^d$-periodic setting

Our setup consists of the following data:

#### Assumption 2.1

$(\mathcal {X}, \mathcal {E})$ is a locally finite and $\mathbb {Z}^d$-periodic connected graph of bounded degree.

More precisely, we assume that

$$\begin{aligned} \mathcal {X}= \mathbb {Z}^d \times V, \end{aligned}$$

where $V$ is a finite set. The coordinates of $x = (z, v) \in \mathcal {X}$ will be denoted by

$$\begin{aligned} x_\textsf{z}:= z, \qquad x_\textsf{v}:= v. \end{aligned}$$

The set of edges $\mathcal {E}\subseteq \mathcal {X}\times \mathcal {X}$ is symmetric and $\mathbb {Z}^d$-periodic, in the sense that

$$\begin{aligned} (x, y) \in \mathcal {E}\quad \text {iff} \quad \big ( S^z(x), S^z(y) \big ) \in \mathcal {E}\text { for all } z \in \mathbb {Z}^d. \end{aligned}$$

Here, $S^{\bar{z}}: \mathcal {X}\rightarrow \mathcal {X}$ is the *shift operator* defined by

$$\begin{aligned} S^{\bar{z}} (x) = (\bar{z} + z, v) \quad \text {for } x = (z, v) \in \mathcal {X}. \end{aligned}$$

We write $x \sim y$ whenever $(x,y) \in \mathcal {E}$.

**Fig. 2:**
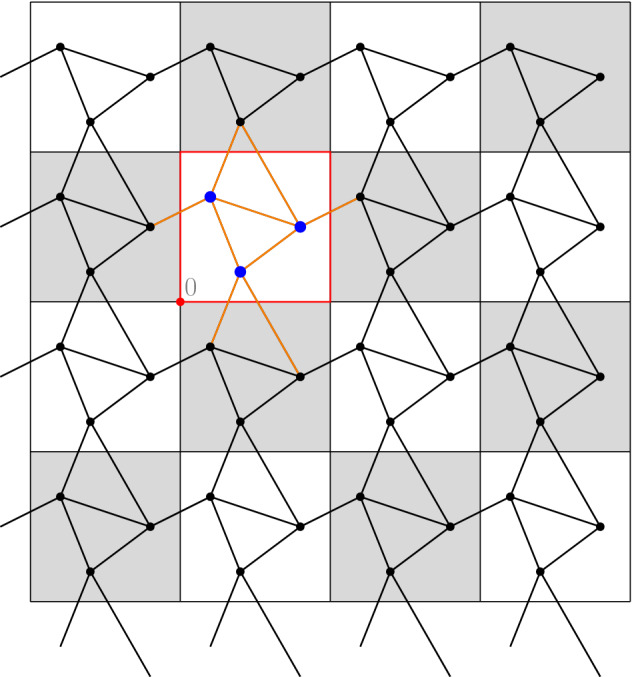
A fragment of a $\mathbb {Z}^d$-periodic graph $(\mathcal {X}, \mathcal {E})$. The blue nodes represent $\mathcal {X}^Q$ and the orange edges represent $\mathcal {E}^Q$ (color figure online)

Let $R_0:= \max _{(x,y) \in \mathcal {E}} | x_\textsf{z}- y_\textsf{z}|_{\ell _\infty ^d}$ be the maximal edge length, measured with respect to the supremum norm $|\cdot |_{\ell _\infty ^d}$ on $\mathbb {R}^d$. It will be convenient to use the notation

$$\begin{aligned} \mathcal {X}^Q:= \{ x \in \mathcal {X}\ : \ x_\textsf{z}= 0 \} \quad \text { and }\quad \mathcal {E}^Q:= \big \{ (x, y) \in \mathcal {E}\ : \ x_\textsf{z}= 0 \big \}. \end{aligned}$$

#### Remark 2.2

(Abstract vs. embedded graphs) Rather than working with abstract $$\mathbb {Z}^d$$-periodic graphs, it is possible to regard $$\mathcal {X}$$ as a $$\mathbb {Z}^d$$-periodic subset of $$\mathbb {R}^d$$, by choosing $$V$$ to be a subset of $$[0,1)^d$$ and using the identification $$(z,v) \equiv z + v$$, see Fig. 2. Since the embedding plays no role in the formulation of the discrete problem, we work with the abstract setup. Note that edges between nodes that are not in adjacent cells are also allowed.

#### Assumption 2.3

(Admissible cost function) The function $F: \mathbb {R}_+^\mathcal {X}\times \mathbb {R}_a^\mathcal {E}\rightarrow \mathbb {R}\cup \{+\infty \}$ is assumed to have the following properties:

- *F* is convex and lower semicontinuous.
- *F* is *local* in the sense that there exists $R_1 < \infty$ such that $F(m,J) = F(m',J')$ whenever $m, m' \in \mathbb {R}_+^\mathcal {X}$ and $J, J' \in \mathbb {R}_a^\mathcal {E}$ agree within a ball of radius $R_1$, i.e.,
  $$\begin{aligned} m(x)&= m'(x){} & {} \text {for all } x \in \mathcal {X}\text { with } |x_\textsf{z}|_{\ell _\infty ^d} \le R_1, \quad and \\ J(x,y)&= J'(x,y){} & {} \text {for all } (x,y) \in \mathcal {E}\text { with } |x_\textsf{z}|_{\ell _\infty ^d}, |y_\textsf{z}|_{\ell _\infty ^d} \le R_1. \end{aligned}$$
- *F* is of at least *linear growth*, i.e., there exist $c > 0$ and $C < \infty$ such that
  2.1 $$\begin{aligned} F(m,J) \ge c \sum _{ (x, y) \in \mathcal {E}^Q} |J(x,y)| - C \Bigg ( 1 + \sum _{\begin{array}{c} x\in \mathcal {X}\\ |x|_{\ell _\infty ^d} \le R \end{array}} m(x) \Bigg ) \end{aligned}$$
  for any $m \in \mathbb {R}_+^\mathcal {X}$ and $J \in \mathbb {R}_a^\mathcal {E}$. Here, $R:= \max \{R_0, R_1\}$.
- There exist a $\mathbb {Z}^d$-periodic function $m^\circ \in \mathbb {R}_+^\mathcal {X}$ and a $\mathbb {Z}^d$-periodic and divergence-free vector field $J^\circ \in \mathbb {R}_a^\mathcal {E}$ such that
  2.2 $$\begin{aligned} ( m^\circ , J^\circ ) \in {{\,\mathrm{\textsf{D}}\,}}(F)^\circ . \end{aligned}$$

#### Remark 2.4

As *F* is local, it depends on finitely many parameters. Therefore, ${{\,\mathrm{\textsf{D}}\,}}(F)^\circ$, the topological interior of the domain ${{\,\mathrm{\textsf{D}}\,}}(F)$ of *F* is defined unambiguously.

#### Remark 2.5

In many examples, the function *F* takes one of the following forms, for suitable functions $F_x$ and $F_{xy}$:

$$\begin{aligned} F(m,J)&= \sum _{x\in \mathcal {X}^Q} F_x\Big ( m(x), \big ( J(x,y) \big )_{y\sim x} \Big ), \quad F(m,J) = \frac{1}{2} \sum _{(x,y) \in \mathcal {E}^Q} F_{xy}\Big (m(x),m(y),J(x,y)\Big ). \end{aligned}$$

We then say that *F* is vertex-based (respectively, edge-based).

#### Remark 2.6

Of particular interest are edge-based functions of the form

2.3 $$\begin{aligned} F(m,J) = \frac{1}{2} \sum _{(x,y) \in \mathcal {E}^Q} \frac{|J(x,y)|^p}{\Lambda \big (q_{xy} m(x), q_{yx} m(y)\big )^{p-1}}, \end{aligned}$$

where $1 \le p < \infty$, the constants $q_{xy}, q_{yx} > 0$ are fixed parameters defined for $(x,y)\in \mathcal {E}^Q$, and $\Lambda$ is a suitable mean (i.e., $\Lambda : \mathbb {R}_+ \times \mathbb {R}_+ \rightarrow \mathbb {R}_+$ is a jointly concave and 1-homogeneous function satisfying $\Lambda (1,1) = 1$). Functions of this type arise naturally in discretisations of Wasserstein gradient-flow structures [11, 30, 32].

We claim that these cost functions satisfy the growth condition (2.1). Indeed, using Young’s inequality $|J| \le \tfrac{1}{p} \tfrac{|J|^p}{\Lambda ^{p-1}} + \tfrac{p-1}{p} \Lambda$ we infer that

$$\begin{aligned} \sum _{(x,y) \in \mathcal {E}^Q} |J(x,y)|&\le \frac{1}{p} \sum _{(x,y) \in \mathcal {E}^Q} \frac{|J(x,y)|^p}{\Lambda \big ( q_{xy} m(x), q_{yx} m(y)\big )^{p-1}} \\ {}&\qquad + \frac{p-1}{p} \sum _{(x,y) \in \mathcal {E}^Q} \Lambda \big (q_{xy} m(x), q_{yx} m(y)\big ) \\ {}&\le \frac{2}{p} F(m,J) + C\sum _{ x \in \mathcal {X}, |x|_{\ell _\infty ^d} \le R_0 } m(x), \end{aligned}$$

with constant $C>0$ depending on $\max _{x,y} (q_{xy} + q_{yx})$. This shows that (2.1) is satisfied.

### Rescaled setting

Let $(\mathcal {X}, \mathcal {E})$ be a locally finite and $\mathbb {Z}^d$-periodic graph as above. Fix $\varepsilon > 0$ such that $\frac{1}{\varepsilon }\in \mathbb {N}$. *The assumption that* $\frac{1}{\varepsilon }\in \mathbb {N}$ *remains in force throughout the paper.*

*The rescaled graph.* Let $\mathbb {T}_\varepsilon ^d = (\varepsilon \mathbb {Z}/ \mathbb {Z})^d$ be the discrete torus of mesh size $\varepsilon$. The corresponding equivalence classes are denoted by $[\varepsilon z]$ for $z \in \mathbb {Z}^d$. To improve readability, we occasionally omit the brackets. Alternatively, we may write $\mathbb {T}_\varepsilon ^d = \varepsilon \mathbb {Z}_\varepsilon ^d$ where $\mathbb {Z}_\varepsilon ^d = \big (\mathbb {Z}/ \tfrac{1}{\varepsilon }\mathbb {Z}\big )^d$.

The rescaled graph $(\mathcal {X}_\varepsilon , \mathcal {E}_\varepsilon )$ is constructed by rescaling the $\mathbb {Z}^d$-periodic graph $(\mathcal {X}, \mathcal {E})$ and wrapping it around the torus. More formally, we consider the finite sets

$$\begin{aligned} \mathcal {X}_\varepsilon := \mathbb {T}_\varepsilon ^d \times V\quad \text {and}\quad \mathcal {E}_\varepsilon := \big \{ \big ( T_\varepsilon ^0 (x), T_\varepsilon ^0 (y) \big ) \ : \ (x, y) \in \mathcal {E}\big \} \end{aligned}$$

where, for $$\bar{z} \in \mathbb {Z}_\varepsilon ^d$$,

2.4 $$\begin{aligned} T_\varepsilon ^{\bar{z}} : \mathcal {X}\rightarrow \mathcal {X}_\varepsilon , \qquad (z, v) \mapsto \big ( [\varepsilon (\bar{z} + z)], v \big ). \end{aligned}$$

Throughout the paper we always assume that $\varepsilon R_0 < \frac{1}{2}$, to avoid that edges in $\mathcal {E}$ “bite themselves in the tail” when wrapped around the torus. For $x = \big ([\varepsilon z], v\big ) \in \mathcal {X}_\varepsilon$ we will write

$$\begin{aligned} x_\textsf{z}:= z \in \mathbb {Z}_\varepsilon ^d, \qquad x_\textsf{v}:= v\in V. \end{aligned}$$

*The rescaled energies.* Let $F: \mathbb {R}_+^\mathcal {X}\times \mathbb {R}_a^\mathcal {E}\rightarrow \mathbb {R}\cup \{ + \infty \}$ be a cost function satisfying Assumption 2.3. For $\varepsilon > 0$ satisfying the conditions above, we shall define a corresponding energy functional $\mathcal {F}_\varepsilon$ in the rescaled periodic setting.

**Fig. 3:**
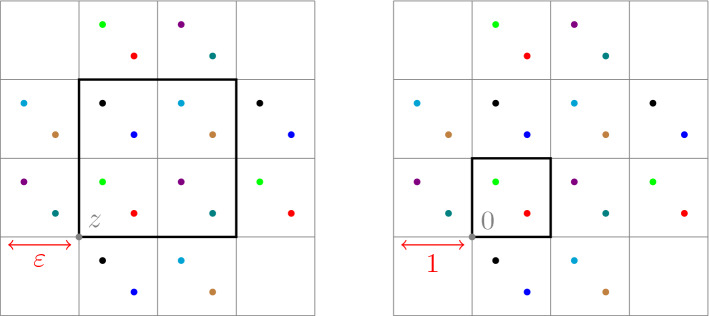
On the left, the values of a function $\psi : \mathcal {X}_\varepsilon \rightarrow \mathbb {R}$ correspond to different colors over the nodes. On the right, the corresponding values of $\tau _\varepsilon ^z \psi : \mathcal {X}\rightarrow \mathbb {R}$ (color figure online)

First we introduce some notation, which we use to transfer functions defined on $\mathcal {X}_\varepsilon$ to $\mathcal {X}$ (and from $\mathcal {E}_\varepsilon$ to $\mathcal {E}$). Let $\bar{z} \in \mathbb {Z}_\varepsilon ^d$. Each function $\psi : \mathcal {X}_\varepsilon \rightarrow \mathbb {R}$ induces a $\frac{1}{\varepsilon }\mathbb {Z}^d$-periodic function

$$\begin{aligned} \tau _\varepsilon ^{\bar{z}} \psi : \mathcal {X}\rightarrow \mathbb {R}, \qquad \big (\tau _\varepsilon ^{\bar{z}} \psi \big )(x) := \psi \big ( T_\varepsilon ^{\bar{z}}(x) \big ) \quad \text { for } x \in \mathcal {X}. \end{aligned}$$

see Fig. 3. Similarly, each function $J: \mathcal {E}_\varepsilon \rightarrow \mathbb {R}$ induces a $\frac{1}{\varepsilon }\mathbb {Z}^d$-periodic function

$$\begin{aligned} \tau _\varepsilon ^{\bar{z}} J : \mathcal {E}\rightarrow \mathbb {R}, \qquad \big ( \tau _\varepsilon ^{\bar{z}} J \big )(x,y) := J \big ( T_\varepsilon ^{\bar{z}}(x), T_\varepsilon ^{\bar{z}}(y) \big ) \quad \text { for } (x,y) \in \mathcal {E}. \end{aligned}$$

#### Definition 2.7

(Discrete energy functional) The rescaled energy is defined by

$$\begin{aligned} \mathcal {F}_\varepsilon : \mathbb {R}_+^{\mathcal {X}_\varepsilon }\times \mathbb {R}_a^{\mathcal {E}_\varepsilon }&\rightarrow \mathbb {R}\cup \{ + \infty \}, \qquad (m, J) \mapsto \sum _{z \in \mathbb {Z}_\varepsilon ^d} \varepsilon ^d F\bigg ( \frac{\tau _\varepsilon ^z m}{\varepsilon ^d} , \frac{\tau _\varepsilon ^z J}{\varepsilon ^{d-1}} \bigg ). \end{aligned}$$

#### Remark 2.8

We note that $\mathcal {F}_\varepsilon (m,J)$ is well-defined as an element in $\mathbb {R}\cup \{ + \infty \}$. Indeed, the (at least) linear growth condition (2.1) yields

$$\begin{aligned} \mathcal {F}_\varepsilon ( m, J ) = \sum _{z \in \mathbb {Z}_\varepsilon ^d} \varepsilon ^d F\bigg ( \frac{\tau _\varepsilon ^z m}{\varepsilon ^d} , \frac{\tau _\varepsilon ^z J}{\varepsilon ^{d-1}} \bigg )&\ge - C \sum _{z \in \mathbb {Z}_\varepsilon ^d} \varepsilon ^d \Bigg ( 1 + \sum _{\begin{array}{c} x\in \mathcal {X}\\ |x|_{\ell _\infty ^d} \le R \end{array}} \frac{\tau _\varepsilon ^z m(x)}{\varepsilon ^d} \Bigg ) \\ {}&\ge - C \bigg ( 1 + (2R + 1)^d \sum _{x \in \mathcal {X}_\varepsilon } m(x) \bigg ) > - \infty . \end{aligned}$$

For ${\bar{z}} \in \mathbb {Z}_\varepsilon ^d$ it will be useful to consider the *shift operator* $S^{\bar{z}}_\varepsilon : \mathcal {X}_\varepsilon \rightarrow \mathcal {X}_\varepsilon$ and $S^{\bar{z}}_\varepsilon : \mathcal {E}_\varepsilon \rightarrow \mathcal {E}_\varepsilon$ defined by

$$\begin{aligned} S^{\bar{z}}_\varepsilon (x)&= \big ( [\varepsilon (\bar{z} + z)], v\big ){} & {} \text {for } x = ([\varepsilon z], v) \in \mathcal {X}_\varepsilon , \\ S^{\bar{z}}_\varepsilon (x,y)&= \big ( S^{\bar{z}}_\varepsilon (x), S^{\bar{z}}_\varepsilon (y) \big ){} & {} \text {for } (x,y) \in \mathcal {E}_\varepsilon . \end{aligned}$$

Moreover, for $\psi :\mathcal {X}_\varepsilon \rightarrow \mathbb {R}$ and $J: \mathcal {E}_\varepsilon \rightarrow \mathbb {R}$ we define

2.5 $$\begin{aligned} \begin{aligned}&\sigma _\varepsilon ^{\bar{z}} \psi : \mathcal {X}_\varepsilon \rightarrow \mathbb {R}, \qquad{} & {} \big (\sigma _\varepsilon ^{\bar{z}} \psi \big )(x) := \psi (S_\varepsilon ^{\bar{z}}(x)){} & {} \text {for} \; x \in \mathcal {X}_\varepsilon , \\&\sigma _\varepsilon ^{\bar{z}}J : \mathcal {E}_\varepsilon \rightarrow \mathbb {R}, \qquad{} & {} \big (\sigma _\varepsilon ^{\bar{z}} J\big )(x,y) := J(S_\varepsilon ^{\bar{z}}(x,y)){} & {} \text {for} \; (x,y) \in \mathcal {E}_\varepsilon . \end{aligned} \end{aligned}$$

#### Definition 2.9

(Discrete continuity equation) A pair $({\pmb {m}}, \pmb {J})$ is said to be a solution to the discrete continuity equation if ${\pmb {m}}: \mathcal {I}\rightarrow \mathbb {R}_+^{\mathcal {X}_\varepsilon }$ is continuous, $\pmb {J}: \mathcal {I}\rightarrow \mathbb {R}_a^{\mathcal {E}_\varepsilon }$ is Borel measurable, and

2.6 $$\begin{aligned} \partial _t m_t(x) + \sum _{y\sim x}J_t(x,y) = 0 \end{aligned}$$

for all $x \in \mathcal {X}_\varepsilon$ in the sense of distributions. We use the notation

$$\begin{aligned} ({\pmb {m}}, \pmb {J}) \in \mathcal{C}\mathcal{E}_\varepsilon ^\mathcal {I}. \end{aligned}$$

#### Remark 2.10

We may write (2.6) as $\partial _t m_t + {{\,\mathrm{\text {\textsf{div}}}\,}}J_t = 0$ using the notation (B.15).

#### Lemma 2.11

(Mass preservation) Let $({\pmb {m}}, \pmb {J}) \in \mathcal{C}\mathcal{E}_\varepsilon ^\mathcal {I}$. Then we have $m_s(\mathcal {X}_\varepsilon ) = m_t(\mathcal {X}_\varepsilon )$ for all $s, t \in \mathcal {I}$.

#### Proof

Without loss of generality, suppose that $s, t \in \mathcal {I}$ with $s < t$. Approximating the characteristic function $\chi _{[s,t]}$ by smooth test functions, we obtain, for all $x \in \mathcal {X}_\varepsilon$,

$$\begin{aligned} m_t(x) - m_s(x) = \int _s^t \sum _{y \sim x} J_r(x,y) \, \textrm{d}r. \end{aligned}$$

Summing the above over $x\in \mathcal {X}_\varepsilon$ and using the anti-symmetry of $\pmb {J}$, the result follows.

We are now ready to define one of the main objects in this paper.

#### Definition 2.12

(Discrete action functional) For any continuous function ${\pmb {m}}: \mathcal {I}\rightarrow \mathbb {R}_+^{\mathcal {X}_\varepsilon }$ such that $t \mapsto \sum _{x \in \mathcal {X}_\varepsilon } m_t(x) \in L^1(\mathcal {I})$ and any Borel measurable function $\pmb {J}: \mathcal {I}\rightarrow \mathbb {R}_a^{\mathcal {E}_\varepsilon }$, we define

$$\begin{aligned} \mathcal {A}_\varepsilon ^\mathcal {I}({\pmb {m}}, \pmb {J})&:= \int _\mathcal {I}\mathcal {F}_\varepsilon ( m_t, J_t ) \, \textrm{d}t \in \mathbb {R}\cup \{ + \infty \}. \end{aligned}$$

Furthermore, we set

$$\begin{aligned} \mathcal {A}_\varepsilon ^\mathcal {I}({\pmb {m}})&:= \inf _{\pmb {J}} \Big \{ \mathcal {A}_\varepsilon ^\mathcal {I}({\pmb {m}}, \pmb {J}) \ : \ ({\pmb {m}}, \pmb {J}) \in \mathcal{C}\mathcal{E}_\varepsilon ^\mathcal {I}\Big \}. \end{aligned}$$

#### Remark 2.13

We claim that $\mathcal {A}_\varepsilon ^\mathcal {I}({\pmb {m}}, \pmb {J})$ is well-defined as an element in $\mathbb {R}\cup \{ + \infty \}$. Indeed, the (at least) linear growth condition (2.1) yields as in Remark 2.8

$$\begin{aligned} \mathcal {F}_\varepsilon ( m_t, J_t ) \ge - C \bigg ( 1 + (2R + 1)^d \sum _{x \in \mathcal {X}_\varepsilon } m_t(x) \bigg ). \end{aligned}$$

for any $t \in \mathcal {I}$. Since $t \mapsto \sum _{x \in \mathcal {X}_\varepsilon } m_t(x) \in L^1(\mathcal {I})$, the claim follows.

In particular, $\mathcal {A}_\varepsilon ^\mathcal {I}({\pmb {m}}, \pmb {J})$ is well-defined whenever $({\pmb {m}}, \pmb {J}) \in \mathcal{C}\mathcal{E}_\varepsilon ^\mathcal {I}$, since $t \mapsto \sum _{x \in \mathcal {X}_\varepsilon } m_t(x)$ is constant by Lemma 2.11.

#### Remark 2.14

If the time interval is clear from the context, we often simply write $\mathcal{C}\mathcal{E}_\varepsilon$ and $\mathcal {A}_\varepsilon$.

The aim of this work is to study the asymptotic behaviour of the energies $\mathcal {A}_\varepsilon ^\mathcal {I}$ as $\varepsilon \rightarrow 0$.

## Dynamical optimal transport in the continuous setting

We shall now define a corresponding class of dynamical optimal transport problems on the continuous torus $\mathbb {T}^d$. We start in Sect. 3.1 by defining the natural continuous analogues of the discrete objects from Sect. 2. In Sect. 3.2 we define generalisations of these objects that have better compactness properties.

### Continuous continuity equation and action functional

First we define solutions to the continuity equation on a bounded open time interval $\mathcal {I}$.

#### Definition 3.1

(Continuity equation) A pair $({\varvec{\mu }}, {\varvec{\nu }})$ is said to be a solution to the continuity equation $\partial _t {\varvec{\mu }}+ \nabla \cdot {\varvec{\nu }}= 0$ if the following conditions holds:

(i) ${\varvec{\mu }}: \mathcal {I}\rightarrow \mathcal {M}_+(\mathbb {T}^d)$ is vaguely continuous;
(ii) ${\varvec{\nu }}: \mathcal {I}\rightarrow \mathcal {M}^d(\mathbb {T}^d)$ is a Borel family satisfying $\int _\mathcal {I}|\nu _t|(\mathbb {T}^d) \, \textrm{d}t < \infty$;
(iii) The equation
  3.1 $$\begin{aligned} \partial _t \mu _t(x) + \nabla \cdot \nu _t(x) = 0 \end{aligned}$$
  holds in the sense of distributions, i.e., for all $\varphi \in \mathcal {C}_c^1\big (\mathcal {I}\times \mathbb {T}^d \big )$,
  $$\begin{aligned} \int _\mathcal {I}\int _{\mathbb {T}^d} \partial _t \varphi _t(x) \, \textrm{d}\mu _t(x) \, \textrm{d}t + \int _\mathcal {I}\int _{\mathbb {T}^d} \nabla \varphi _t(x) \cdot \, \textrm{d}\nu _t(x) \, \textrm{d}t = 0. \end{aligned}$$

We use the notation

$$\begin{aligned} ({\varvec{\mu }}, {\varvec{\nu }}) \in \mathcal{C}\mathcal{E}^\mathcal {I}. \end{aligned}$$

We will consider the energy densities *f* with the following properties.

#### Assumption 3.2

Let $f: \mathbb {R}_+ \times \mathbb {R}^d \rightarrow \mathbb {R}\cup \{+\infty \}$ be a lower semicontinuous and convex function, whose domain has nonempty interior. We assume that there exist constants $c > 0$ and $C < \infty$ such that the (at least) linear growth condition

3.2 $$\begin{aligned} f(\rho , j) \ge c |j| - C (\rho + 1) \end{aligned}$$

holds for all $\rho \in \mathbb {R}_+$ and $j \in \mathbb {R}^d$.

The corresponding *recession function* $f^\infty : \mathbb {R}_+ \times \mathbb {R}^d \rightarrow \mathbb {R}\cup \{+\infty \}$ is defined by

$$\begin{aligned} f^\infty (\rho ,j) := \lim _{t \rightarrow + \infty } \frac{f(\rho _0 + t \rho , j_0 + t j)}{t}, \end{aligned}$$

where $(\rho _0, j_0) \in {{\,\mathrm{\textsf{D}}\,}}(f)$ is arbitrary. It is well known that the function $f^\infty$ is lower semicontinuous and convex, and it satisfies

3.3 $$\begin{aligned} f^\infty (\rho ,j) \ge c|j| - C\rho . \end{aligned}$$

We refer to [2, Section 2.6] for a proof of these facts.

Let $\mathscr {L}^d$ denote the Lebesgue measure on $\mathbb {T}^d$. For $\mu \in \mathcal {M}_+(\mathbb {T}^d)$ and $\nu \in \mathcal {M}^d(\mathbb {T}^d)$ we consider the Lebesgue decompositions given by

$$\begin{aligned} \mu = \rho \mathscr {L}^d + \mu ^\perp , \qquad \nu = j \mathscr {L}^d + \nu ^\perp \end{aligned}$$

for some $\rho \in L_+^1(\mathbb {T}^d)$ and $j \in L^1(\mathbb {T}^d; \mathbb {R}^d)$. It is always possible to introduce a measure $\sigma \in \mathcal {M}_+(\mathbb {T}^d)$ such that

$$\begin{aligned} \mu ^\perp = \rho ^\perp \sigma , \qquad \nu ^\perp = j^\perp \sigma , \end{aligned}$$

for some $\rho ^\perp \in L_+^1(\sigma )$ and $j^\perp \in L^1(\sigma ;\mathbb {R}^d)$. (Take, for instance, $\sigma = \mu ^\perp + |\nu ^\perp |$.) Using this notation we define the continuous energy as follows.

#### Definition 3.3

(Continuous energy functional) Let $f: \mathbb {R}_+ \times \mathbb {R}^d \rightarrow \mathbb {R}\cup \{ + \infty \}$ satisfy Assumption 3.2. We define the continuous energy functional by

$$\begin{aligned}&\mathbb {F}: \mathcal {M}_+( \mathbb {T}^d) \times \mathcal {M}^d(\mathbb {T}^d) \rightarrow \mathbb {R}\cup \{ +\infty \}, \\&\mathbb {F}(\mu , \nu ) := \int _{\mathbb {T}^d} f \big ( \rho (x), j(x) \big ) \, \textrm{d}x + \int _{\mathbb {T}^d} f^\infty \big ( \rho ^\perp (x), j^\perp (x) \big ) \, \textrm{d}\sigma (x). \end{aligned}$$

#### Remark 3.4

By 1-homogeneity of $f^\infty$, this definition does not depend on the choice of the measure $\sigma \in \mathcal {M}_+(\mathbb {T}^d)$.

#### Definition 3.5

(Action functional) For any curve ${\varvec{\mu }}: \mathcal {I}\rightarrow \mathcal {M}_+(\mathbb {T}^d)$ with $\int _\mathcal {I}\mu _t(\mathbb {T}^d) \, \textrm{d}t < \infty$ and any Borel measurable curve ${\varvec{\nu }}: \mathcal {I}\rightarrow \mathcal {M}^d(\mathbb {T}^d)$ we define

$$\begin{aligned} \mathbb {A}^\mathcal {I}({\varvec{\mu }}, {\varvec{\nu }})&:= \int _\mathcal {I}\mathbb {F}( \mu _t, \nu _t ) \, \textrm{d}t. \end{aligned}$$

Furthermore, we set

$$\begin{aligned} \mathbb {A}^\mathcal {I}({\varvec{\mu }})&:= \inf _{{\varvec{\nu }}} \Big \{ \mathbb {A}^\mathcal {I}({\varvec{\mu }}, {\varvec{\nu }}) \ : \ ({\varvec{\mu }}, {\varvec{\nu }}) \in \mathbb{C}\mathbb{E}^\mathcal {I}\Big \}. \end{aligned}$$

#### Remark 3.6

As $f(\rho , j) \ge - C (1 + \rho )$ by (3.2), the assumption $\int _\mathcal {I}\mu _t(\mathbb {T}^d) \, \textrm{d}t < \infty$ ensures that $\mathbb {A}^\mathcal {I}({\varvec{\mu }}, {\varvec{\nu }})$ is well-defined in $\mathbb {R}\cup \{ + \infty \}$.

#### Remark 3.7

(Dependence on time intervals) Remark 2.14 applies in the continuous setting as well. If the time interval is clear from the context, we often simply write $\mathbb{C}\mathbb{E}$ and $\mathbb {A}$.

Under additional assumptions on the function *f*, it is possible to prove compactness for families of solutions to the continuity equation with bounded action; see [13, Corollary 4.10]. However, in our general setting, such a compactness result fails to hold, as the following example shows.

#### Example 3.8

(Lack of compactness) To see this, let $y^\varepsilon (t)$ be the position of a particle of mass *m* that moves from 0 to $\bar{y} \in [0,\frac{1}{2}]^d$ in the time interval $(a_\varepsilon , b_\varepsilon ):= \big (\tfrac{1-\varepsilon }{2}, \tfrac{1+\varepsilon }{2}\big )$ with constant speed $\frac{|\bar{y}|}{\varepsilon }$. At all other times in the time interval $\mathcal {I}= (0,1)$ the particle is at rest:

$$\begin{aligned} y^\varepsilon (t) = {\left\{ \begin{array}{ll} 0, &{} t \in [0, a_\varepsilon ], \\ \big (t - \tfrac{1}{2}(1 - \varepsilon )\big ) \varepsilon ^{-1} \bar{y}, &{} t \in \big (a_\varepsilon , b_\varepsilon ),\\ \bar{y} &{} t \in [b_\varepsilon , 1].\\ \end{array}\right. } \end{aligned}$$

The associated solution $({\varvec{\mu }}^\varepsilon , {\varvec{\nu }}^\varepsilon )$ to the continuity equation $\partial _t {\varvec{\mu }}^\varepsilon + \nabla \cdot {\varvec{\nu }}^\varepsilon = 0$ is given by

$$\begin{aligned} \mu _t^\varepsilon (\textrm{d}x) := m \delta _{y^\varepsilon (t)}(\textrm{d}x) , \qquad \nu _t^\varepsilon (\textrm{d}x) := \frac{m|\bar{y}|}{\varepsilon } \chi _{(a_\varepsilon , b_\varepsilon )}(t) \delta _{y^\varepsilon (t)}(\textrm{d}x). \end{aligned}$$

Let $f(\rho , j) = |j|$ be the total momentum, which satisfies Assumption 3.2. We then have $\mathbb {F}(\mu _t^\varepsilon , \nu _t^\varepsilon ) = \frac{m|\bar{y}|}{\varepsilon } {\textbf{1}}_{(a_\varepsilon , b_\varepsilon )}(t)$, hence $\mathbb {A}^\mathcal {I}({\varvec{\mu }}^\varepsilon , {\varvec{\nu }}^\varepsilon ) = m \bar{y}$, independently of $\varepsilon$.

However, as $\varepsilon \rightarrow 0$, the motion converges to the discontinuous curve given by $\mu _t = \delta _0$ for $t \in [0,\tfrac{1}{2})$ and $\mu _t = \delta _{\bar{y}}$ for $t \in (\tfrac{1}{2}, 1]$. In particular, it does not satisfy the continuity equation in the sense above.

### Generalised continuity equation and action functional

In view of this lack of compactness, we will extend the definition of the continuity equation and the action functional to more general objects.

#### Definition 3.9

(Continuity equation) A pair of measures $({\varvec{\mu }}, {\varvec{\nu }}) \in \mathcal {M}_+\big (\mathcal {I}\times \mathbb {T}^d\big ) \times \mathcal {M}^d\big (\mathcal {I}\times \mathbb {T}^d\big )$ is said to be a solution to the continuity equation

3.4 $$\begin{aligned} \partial _t {\varvec{\mu }}+ \nabla \cdot {\varvec{\nu }}= 0 \end{aligned}$$

if, for all $\varphi \in \mathcal {C}_c^1\big (\mathcal {I}\times \mathbb {T}^d\big )$, we have

$$\begin{aligned} \int _{\mathcal {I}\times \mathbb {T}^d} \partial _t \varphi \, \textrm{d}{\varvec{\mu }}+ \int _{\mathcal {I}\times \mathbb {T}^d} \nabla \varphi \cdot \, \textrm{d}{\varvec{\nu }}= 0. \end{aligned}$$

As above, we use the notation $({\varvec{\mu }}, {\varvec{\nu }}) \in \mathbb{C}\mathbb{E}^\mathcal {I}$.

Clearly, this definition is consistent with Definition 3.5.

Let us now extend the action functional $\mathbb {A}^\mathcal {I}$ as well. For this purpose, let $\mathscr {L}^{d+1}$ denote the Lebesgue measure on $\mathcal {I}\times \mathbb {T}^d$. For ${\varvec{\mu }}\in \mathcal {M}_+\big ( \mathcal {I}\times \mathbb {T}^d\big )$ and ${\varvec{\nu }}\in \mathcal {M}^d\big ( \mathcal {I}\times \mathbb {T}^d\big )$ we consider the Lebesgue decompositions given by

$$\begin{aligned} {\varvec{\mu }}= \rho \mathscr {L}^{d+1} + {\varvec{\mu }}^\perp , \qquad {\varvec{\nu }}= j \mathscr {L}^{d+1} + {\varvec{\nu }}^\perp \end{aligned}$$

for some $\rho \in L_+^1\big ( \mathcal {I}\times \mathbb {T}^d \big )$ and $j \in L^1\big ( \mathcal {I}\times \mathbb {T}^d; \mathbb {R}^d\big )$. As above, it is always possible to introduce a measure ${\varvec{\sigma }}\in \mathcal {M}_+( \mathcal {I}\times \mathbb {T}^d)$ such that

3.5 $$\begin{aligned} {\varvec{\mu }}^\perp = \rho ^\perp {\varvec{\sigma }}, \qquad {\varvec{\nu }}^\perp = j^\perp {\varvec{\sigma }}, \end{aligned}$$

for some $\rho ^\perp \in L_+^1({\varvec{\sigma }})$ and $j^\perp \in L^1({\varvec{\sigma }};\mathbb {R}^d)$.

#### Definition 3.10

(Action functional) We define the action by

$$\begin{aligned}&\mathbb {A}^\mathcal {I}: \mathcal {M}_+\big ( \mathcal {I}\times \mathbb {T}^d\big ) \times \mathcal {M}^d\big ( \mathcal {I}\times \mathbb {T}^d\big ) \rightarrow \mathbb {R}\cup \{ +\infty \}, \\&\mathbb {A}^\mathcal {I}({\varvec{\mu }}, {\varvec{\nu }}) := \int _{\mathcal {I}\times \mathbb {T}^d} f \big ( \rho _t(x), j_t(x) \big ) \, \textrm{d}x \, \textrm{d}t + \int _{ \mathcal {I}\times \mathbb {T}^d} f^\infty \big ( \rho ^\perp _t(x), j^\perp _t(x) \big ) \, \textrm{d}{\varvec{\sigma }}(t,x). \end{aligned}$$

Furthermore, we set

$$\begin{aligned} \mathbb {A}^\mathcal {I}({\varvec{\mu }}) := \inf _{{\varvec{\nu }}} \{ \mathbb {A}^\mathcal {I}({\varvec{\mu }},{\varvec{\nu }}) : ({\varvec{\mu }},{\varvec{\nu }}) \in \mathbb{C}\mathbb{E}^\mathcal {I}\}. \end{aligned}$$

#### Remark 3.11

This definition does not depend on the choice of ${\varvec{\sigma }}$, in view of the 1-homogeneity of $f^\infty$. As $f(\rho ,j) \ge - C(1+\rho )$ and $f_\infty (\rho ,j) \ge - C \rho$ from (3.2) and (3.3), the fact that ${\varvec{\mu }}(\mathcal {I}\times \mathbb {T}^d) <\infty$ ensures that $\mathbb {A}^\mathcal {I}({\varvec{\mu }}, {\varvec{\nu }})$ is well-defined in $\mathbb {R}\cup \{ + \infty \}$.

#### Example 3.12

(Lack of compactness) Continuing Example 3.8, we can now describe the limiting jump process as a solution to the generalised continuity equation. Consider the measures ${\varvec{\mu }}^\varepsilon \in \mathcal {M}_+(\mathcal {I}\times \mathbb {T}^d)$ and ${\varvec{\nu }}^\varepsilon \in \mathcal {M}^d(\mathcal {I}\times \mathbb {T}^d)$ defined by

$$\begin{aligned} {\varvec{\mu }}^\varepsilon (\textrm{d}x, \textrm{d}t) = \mu _t^\varepsilon (\textrm{d}x) \, \textrm{d}t, \qquad {\varvec{\nu }}^\varepsilon (\textrm{d}x, \textrm{d}t) = \nu _t^\varepsilon (\textrm{d}x) \, \textrm{d}t. \end{aligned}$$

Then we have ${\varvec{\mu }}^\varepsilon \rightarrow {\varvec{\mu }}$ and ${\varvec{\nu }}^\varepsilon \rightarrow {\varvec{\nu }}$ weakly, respectively, in $\mathcal {M}_+(\mathcal {I}\times \mathbb {T}^d)$ and $\mathcal {M}^d(\mathcal {I}\times \mathbb {T}^d)$, where ${\varvec{\mu }}$ represents the discontinuous curve

$$\begin{aligned} {\varvec{\mu }}(\textrm{d}x, \textrm{d}t) = \, \textrm{d}\mu _t(x) \, \textrm{d}t, \quad \text {where } \mu _t = {\left\{ \begin{array}{ll} \delta _0, &{} t \in [0,\tfrac{1}{2}), \\ \delta _{\bar{y}}, &{} t \in (\tfrac{1}{2}, 1]. \end{array}\right. } \end{aligned}$$

The measure ${\varvec{\nu }}$ does *not* admit a disintegration with respect to the Lebesgue measure on $\mathcal {I}$; in other words, it is not associated to a curve of measures on $\mathbb {T}^d$. We have

$$\begin{aligned} {\varvec{\nu }}(\textrm{d}x, \textrm{d}t) = m|\bar{y}| \mathscr {H}^1|_{[0,\bar{y}]}(\textrm{d}x) \delta _{1/2}(\textrm{d}t). \end{aligned}$$

Here $\mathscr {H}^1|_{[0,\bar{y}]}$ denotes the 1-dimensional Hausdorff measure on the (shortest) line segment connecting 0 and $\bar{y}$.

Note that $({\varvec{\mu }}, {\varvec{\nu }})$ solves the continuity equation, as $\mathbb{C}\mathbb{E}^\mathcal {I}$ is stable under joint weak-convergence. Furthermore, we have $\mathbb {A}^\mathcal {I}({\varvec{\mu }}, {\varvec{\nu }}) = m \bar{y}$.

The next result shows that any solution to the continuity equation $({\varvec{\mu }}, {\varvec{\nu }}) \in \mathbb{C}\mathbb{E}^\mathcal {I}$ induces a (not necessarily continuous) curve of measures $(\mu _t)_t \in \mathcal {I}$. The measure ${\varvec{\nu }}$ is not always associated to a curve of measures on $\mathcal {I}$; see Example 3.12. We refer to “Appendix 1” for the definition of $\textrm{BV}_{\textrm{KR}}(\mathcal {I}; \mathcal {M}_+(\mathbb {T}^d))$.

#### Lemma 3.13

(Disintegration of solutions to $\mathbb{C}\mathbb{E}^\mathcal {I}$) Let $({\varvec{\mu }}, {\varvec{\nu }}) \in \mathbb{C}\mathbb{E}^{\mathcal {I}}$. Then $\, \textrm{d}{\varvec{\mu }}(t,x) = \, \textrm{d}\mu _t(x) \, \textrm{d}t$ for some measurable curve $t \mapsto \mu _t \in \mathcal {M}_+(\mathbb {T}^d)$ with finite constant mass. If $\mathbb {A}^{\mathcal {I}}({\varvec{\mu }}) < \infty$, then this curve belongs to $\textrm{BV}_{\textrm{KR}}(\mathcal {I}; \mathcal {M}_+(\mathbb {T}^d))$ and

3.6 $$\begin{aligned} \Vert {\varvec{\mu }}\Vert _{\textrm{BV}_{\textrm{KR}}(\mathcal {I}; \mathcal {M}_+(\mathbb {T}^d))} \le |{\varvec{\nu }}| \big ( \mathcal {I}\times \mathbb {T}^d\big ). \end{aligned}$$

#### Proof

Let $\lambda \in \mathcal {M}_+(\mathcal {I})$ be the time-marginal of ${\varvec{\mu }}$, i.e., $\lambda := (e_1)_{\#} {\varvec{\mu }}$ where $e_1: \mathcal {I}\times \mathbb {T}^d\rightarrow \mathcal {I}$, $e_1(t,x) = t$. We claim that $\lambda$ is a constant multiple of the Lebesgue measure on $\mathcal {I}$. By the disintegration theorem (see, e.g., [3, Theorem 5.3.1]), this implies the first part of the result.

To prove the claim, note that the continuity equation $\mathbb{C}\mathbb{E}^{\mathcal {I}}$ yields

3.7 $$\begin{aligned} \int _{\mathcal {I}} \partial _t \varphi (t) \, \textrm{d}\lambda (t) = \int _{\mathcal {I}\times \mathbb {T}^d} \partial _t \varphi (t) \, \textrm{d}{\varvec{\mu }}(t,x) = 0 \end{aligned}$$

for all $$\varphi \in C_c^\infty (\mathcal {I})$$.

Write $\mathcal {I}= (a,b)$, let $\psi \in C_c^\infty (\mathcal {I})$ be arbitrary, and set $\bar{\psi }:= \frac{1}{|\mathcal {I}|}\int _\mathcal {I}\psi (t) \, \textrm{d}t$. We define $\varphi (t) = \int _a^t \psi (s) \, \textrm{d}s - (t-a) \bar{\psi }$. Then $\varphi \in C_c^\infty (\mathcal {I})$ and $\partial _t \varphi = \psi - \bar{\psi }$. Applying (3.7) we obtain $\int _\mathcal {I}(\psi - \bar{\psi })\, \textrm{d}\lambda = 0$, which implies the claim, and hence the first part of the result.

To prove the second part, suppose that ${\varvec{\mu }}\in \mathcal {M}_+(\mathcal {I}\times \mathbb {T}^d)$ has finite action, and let ${\varvec{\nu }}\in \mathcal {M}^d\big ( \mathcal {I}\times \mathbb {T}^d\big )$ be a solution to the continuity equation (3.4). Applying (3.4) to a test function $\varphi \in \mathcal {C}_c^1\big ( \mathcal {I}; \mathcal {C}^1(\mathbb {T}^d) \big ) \subseteq \mathcal {C}_c^1\big ( \mathcal {I}\times \mathbb {T}^d\big )$ such that $\max _{t\in \mathcal {I}}\Vert \varphi _t\Vert _{\mathcal {C}^1(\mathbb {T}^d)} \le 1$, we obtain

3.8 $$\begin{aligned} \int _{\mathcal {I}\times \mathbb {T}^d} \partial _t \varphi _t \, \textrm{d}\mu _t \, \textrm{d}t = - \int _{\mathcal {I}\times \mathbb {T}^d} \nabla \varphi \cdot \, \textrm{d}{\varvec{\nu }}\le |{\varvec{\nu }}| \big ( \mathcal {I}\times \mathbb {T}^d\big ) < \infty , \end{aligned}$$

which implies the desired bound in view of (B.14).

The next lemma deals with regularity properties for curves of measures with finite action and fine properties for the functionals $\mathbb {A}$ defined in Definition 3.10 with $f=f_\textrm{hom}$.

#### Lemma 3.14

(Properties of $\mathbb {A}^\mathcal {I}$) Let $\mathcal {I}\subset \mathbb {R}$ be a bounded open interval. The following statements hold:

(i) The functionals $({\varvec{\mu }}, {\varvec{\nu }}) \mapsto \mathbb {A}^\mathcal {I}({\varvec{\mu }}, {\varvec{\nu }})$ and ${\varvec{\mu }}\mapsto \mathbb {A}^\mathcal {I}({\varvec{\mu }})$ are convex.
(ii) Let ${\varvec{\mu }}\in \mathcal {M}_+(\mathcal {I}\times \mathbb {T}^d)$. Let $\{\mathcal {I}_n\}_n$ be a sequence of bounded open intervals such that $\mathcal {I}_n \subseteq \mathcal {I}$ and $|\mathcal {I}\setminus \mathcal {I}_n| \rightarrow 0$ as $n \rightarrow \infty$. Let ${\varvec{\mu }}^n \in \mathcal {M}_+(\mathcal {I}_n \times \mathbb {T}^d)$ be such that^2^
  $$\begin{aligned} {\varvec{\mu }}^n \rightarrow {\varvec{\mu }}\ \text {vaguely in} \ \mathcal {M}_+(\mathcal {I}\times \mathbb {T}^d) \ \text { and } \ {\varvec{\mu }}^n(\mathcal {I}_n \times \mathbb {T}^d) \rightarrow {\varvec{\mu }}(\mathcal {I}\times \mathbb {T}^d). \end{aligned}$$
  as $$n \rightarrow \infty $$. Then:
  3.9 $$\begin{aligned} \liminf _{n \rightarrow \infty } \mathbb {A}^{\mathcal {I}_n}({\varvec{\mu }}^n) \ge \mathbb {A}^{\mathcal {I}}({\varvec{\mu }}). \end{aligned}$$
  If, additionally, ${\varvec{\nu }}\in \mathcal {M}^d(\mathcal {I}\times \mathbb {T}^d)$ and ${\varvec{\nu }}^n \in \mathcal {M}^d(\mathcal {I}_n \times \mathbb {T}^d)$ satisfy ${\varvec{\nu }}^n \rightarrow {\varvec{\nu }}$ vaguely in $\mathcal {M}^d(\mathcal {I}\times \mathbb {T}^d)$, then we have
  3.10 $$\begin{aligned} \liminf _{n \rightarrow \infty } \mathbb {A}^{\mathcal {I}_n}({\varvec{\mu }}^n, {\varvec{\nu }}^n) \ge \mathbb {A}^{\mathcal {I}}({\varvec{\mu }}, {\varvec{\nu }}). \end{aligned}$$
  In particular, the functionals $({\varvec{\mu }}, {\varvec{\nu }}) \mapsto \mathbb {A}^\mathcal {I}({\varvec{\mu }}, {\varvec{\nu }})$ and ${\varvec{\mu }}\mapsto \mathbb {A}^\mathcal {I}({\varvec{\mu }})$ are lower semicontinuous with respect to (joint) vague convergence.

#### Proof

*(i)*: Convexity of $\mathbb {A}^\mathcal {I}$ follows from convexity of *f*, $f^\infty$, and the linearity of the constraint (3.4).

*(ii)*: First we show (3.10). Consider the convex energy density $g(\rho ,j):= f(\rho ,j) + C(\rho + 1)$, which is nonnegative by (2.1). Let $\mathbb {A}_g$ be the corresponding action functional defined using *g* instead of *f*. Using the nonnegativity of *g*, the fact that $|\mathcal {I}{\setminus } \mathcal {I}_n| \rightarrow 0$, and the lower semicontinuity result from [2, Theorem 2.34], we obtain

$$\begin{aligned} \liminf _{n\rightarrow \infty } \mathbb {A}_g^{\mathcal {I}_n}({\varvec{\mu }}^n,{\varvec{\nu }}^n ) \ge \liminf _{n\rightarrow \infty } \mathbb {A}_g^{\widetilde{\mathcal {I}}}({\varvec{\mu }}^n,{\varvec{\nu }}^n ) \ge \mathbb {A}_g^{\widetilde{\mathcal {I}}}({\varvec{\mu }}, {\varvec{\nu }}). \end{aligned}$$

for every open interval $\widetilde{\mathcal {I}} \Subset \mathcal {I}$. Taking the supremum over $\widetilde{\mathcal {I}}$, we obtain

3.11 $$\begin{aligned} \liminf _{n\rightarrow \infty } \mathbb {A}_g^{\mathcal {I}_n}({\varvec{\mu }}^n,{\varvec{\nu }}^n ) \ge \mathbb {A}_g^{\mathcal {I}}({\varvec{\mu }}, {\varvec{\nu }}). \end{aligned}$$

Since we have ${\varvec{\mu }}^n\big ( \mathcal {I}_n \times \mathbb {T}^d\big ) \rightarrow {\varvec{\mu }}\big ( \mathcal {I}\times \mathbb {T}^d\big )$ by assumption, the desired result (3.10) follows from (3.11) and the identity

$$\begin{aligned} \begin{aligned} \mathbb {A}_g^{\mathcal {I}_n}({\varvec{\mu }}^n, {\varvec{\nu }}^n) = \mathbb {A}^{\mathcal {I}_n}({\varvec{\mu }}^n, {\varvec{\nu }}^n) + C \Big ( {\varvec{\mu }}^n(\mathcal {I}_n\times \mathbb {T}^d) + 1 \Big ). \end{aligned} \end{aligned}$$

Let us now show (3.9). Let $\{{\varvec{\mu }}^n\}_n \subseteq \mathcal {M}_+\big ( \mathcal {I}_n \times \mathbb {T}^d\big )$ be such that $\sup _n \mathbb {A}^{\mathcal {I}_n}({\varvec{\mu }}^n) < \infty$ and ${\varvec{\mu }}^n \rightarrow {\varvec{\mu }}$ vaguely in $\mathcal {M}_+(\mathcal {I}\times \mathbb {T}^d)$. Let ${\varvec{\nu }}^n \in \mathcal {M}^d(\mathcal {I}_n \times \mathbb {T}^d)$ be such that $({\varvec{\mu }}^n, {\varvec{\nu }}^n) \in \mathbb{C}\mathbb{E}^{\mathcal {I}_n}$ and

$$\begin{aligned} \mathbb {A}^{\mathcal {I}_n}({\varvec{\mu }}^n, {\varvec{\nu }}^n) \le \mathbb {A}^{\mathcal {I}_n}({\varvec{\mu }}^n) + \frac{1}{n}. \end{aligned}$$

From Lemma 3.13, we infer that $\, \textrm{d}{\varvec{\mu }}^n(t,x) = \, \textrm{d}\mu _t^n(x) \, \textrm{d}t$ where $(\mu _t^n)_{t \in \mathcal {I}_n}$ is a curve of constant total mass $c_n:= \mu _t^n(\mathbb {T}^d)$. Moreover, $M:= \sup _n c_n <+\infty$, since ${\varvec{\mu }}^n \rightarrow {\varvec{\mu }}$ vaguely. The growth condition (3.2) implies that

$$\begin{aligned} \sup _n |{\varvec{\nu }}^n|\big ( \mathcal {I}^n \times \mathbb {T}^d\big ) \le \frac{1}{c} \sup _n \mathbb {A}^{\mathcal {I}_n}({\varvec{\mu }}^n) + \frac{C|\mathcal {I}|}{c} \big ( M + 1 \big ) < \infty . \end{aligned}$$

Hence, by the Banach–Alaoglu theorem, there exists a subsequence of $\{{\varvec{\nu }}^n\}_n$ (still indexed by *n*) such that ${\varvec{\nu }}^n \rightarrow {\varvec{\nu }}$ vaguely in $\mathcal {M}^d(\mathcal {I}\times \mathbb {T}^d)$ and $({\varvec{\mu }}, {\varvec{\nu }}) \in \mathbb{C}\mathbb{E}^{\mathcal {I}}$. Another application of Lemma 3.13 ensures that $\, \textrm{d}{\varvec{\mu }}(t,x) = \, \textrm{d}\mu _t(x) \, \textrm{d}t$ where $(\mu _t)_{t \in \mathcal {I}}$ is of constant mass $c:= \mu _t(\mathbb {T}^d) = \lim _{n \rightarrow \infty } c_n$.

We can thus apply the first part of *(ii)* to obtain

$$\begin{aligned} \mathbb {A}^{\mathcal {I}}({\varvec{\mu }}) \le \mathbb {A}^{\mathcal {I}}({\varvec{\mu }}, {\varvec{\nu }}) \le \liminf _{n \rightarrow \infty } \mathbb {A}^{\mathcal {I}_n}( {\varvec{\mu }}^n, {\varvec{\nu }}^n ) = \liminf _{n \rightarrow \infty } \mathbb {A}^{\mathcal {I}_n}( {\varvec{\mu }}^n ), \end{aligned}$$

which ends the proof.

## The homogenised transport problem

Throughout this section we assume that $(\mathcal {X},\mathcal {E})$ safisfies Assumption 2.1 and *F* safisfies Assumption 2.3.

### Discrete representation of continuous measures and vector fields

To define $f_\textrm{hom}$, the following definition turns out to be natural.

#### Definition 4.1

(Representation)

(i) We say that $m\in \mathbb {R}_+^{\mathcal {X}}$ represents $\rho \in \mathbb {R}_+$ if *m* is $\mathbb {Z}^d$-periodic and
  $$\begin{aligned} \sum _{x \in \mathcal {X}^Q} m(x) = \rho . \end{aligned}$$
(ii) We say that $J\in \mathbb {R}_a^{\mathcal {E}}$ represents a vector $j \in \mathbb {R}^d$ if
  - *J* is $$\mathbb {Z}^d$$-periodic;
  - *J* is divergence-free (i.e., ${{\,\mathrm{\text {\textsf{div}}}\,}}J(x) = 0$ for all $x \in \mathcal {X}$);
  - The effective flux of *J* equals *j*; i.e., ${{\,\mathrm{\textsf{Eff}}\,}}(J) = j$, where
    4.1 $$\begin{aligned} {{\,\mathrm{\textsf{Eff}}\,}}(J) := \frac{1}{2} \sum _{(x,y) \in \mathcal {E}^Q} J(x,y) \big ( y_\textsf{z}- x_\textsf{z}\big ). \end{aligned}$$

We use the (slightly abusive) notation $m \in {{\,\mathrm{{\textsf{Rep}}}\,}}(\rho )$ and $J \in {{\,\mathrm{{\textsf{Rep}}}\,}}(j)$. We will also write ${{\,\mathrm{{\textsf{Rep}}}\,}}(\rho ,j) = {{\,\mathrm{{\textsf{Rep}}}\,}}(\rho ) \times {{\,\mathrm{{\textsf{Rep}}}\,}}(j)$.

#### Remark 4.2

Let us remark that $x_\textsf{z}= 0$ in the formula for ${{\,\mathrm{\textsf{Eff}}\,}}(J)$, since $x_\textsf{z}\in \mathcal {X}^Q$.

#### Remark 4.3

The definition of the effective flux ${{\,\mathrm{\textsf{Eff}}\,}}(J)$ is natural in view of Lemmas 4.9 and 4.11 below. These results show that a solution to the continuous continuity equation can be constructed starting from a solution to the discrete continuity equation, with a vector field of the form (4.1).

Clearly, ${{\,\mathrm{{\textsf{Rep}}}\,}}(\rho ) \ne \emptyset$ for every $\rho \in \mathbb {R}_+$. It is also true, though less obvious, that ${{\,\mathrm{{\textsf{Rep}}}\,}}(j) \ne \emptyset$ for every $j \in \mathbb {R}^d$. We will show this in Lemma 4.5 using the $\mathbb {Z}^d$-periodicity and the connectivity of $(\mathcal {X}, \mathcal {E})$.

To prove the result, we will first introduce a natural vector field associated to each simple directed path *P* on $(\mathcal {X},\mathcal {E})$, For an edge $e =(x,y) \in \mathcal {E}$, the corresponding reversed edge will be denoted by $\overline{e} = (y,x) \in \mathcal {E}$.

#### Definition 4.4

**Fig. 4:**
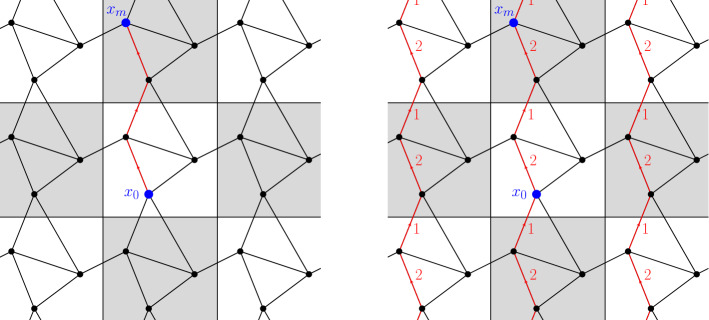
In the first figure, in red, the (directed) path *P* from $x_0$ to $x_m$, support of the vector field $J_P$. In the second one, in red, the support of the vector field $\widetilde{J}_P$ and its values (color figure online)

(Unit flux through a path; see Fig. 4) Let $P:= \{x_i\}_{i=0}^m$ be a simple path in $(\mathcal {X}, \mathcal {E})$, thus $e_i = (x_{i-1}, x_i) \in \mathcal {E}$ for $i = 1, \ldots , m$, and $x_i \ne x_k$ for $i \ne k$. The *unit flux through P* is the discrete field $J_{P} \in \mathbb {R}^{\mathcal {E}}_a$ given by

4.2 $$\begin{aligned} J_P(e) = {\left\{ \begin{array}{ll} 1 &{} \text {if } e = e_i \text { for some } i, \\ -1 &{} \text {if } e = \overline{e}_i \text { for some } i,\\ 0 &{} \text {otherwise} \end{array}\right. } \end{aligned}$$

The *periodic unit flux through P* is the vector field $\widetilde{J}_P \in \mathbb {R}^{\mathcal {E}}_a$ defined by

4.3 $$\begin{aligned} \widetilde{J}_P(e) = \sum _{z \in \mathbb {Z}^d} J_P(T_z e) \quad \text {for } e \in \mathcal {E}. \end{aligned}$$

In the next lemma we collect some key properties of these vector fields. Recall the definition of the discrete divergence in (B.15).

#### Lemma 4.5

(Properties of $J_P$) Let $P:= \{x_i\}_{i=0}^m$ be a simple path in $(\mathcal {X}, \mathcal {E})$.

(i) The discrete divergence of the associated unit flux $J_P: \mathcal {E}\rightarrow \mathbb {R}$ is given by
  4.4 $$\begin{aligned} {{\,\mathrm{\text {\textsf{div}}}\,}}J_P = {\textbf{1}}_{\{x_0\}} - {\textbf{1}}_{\{x_m\}}. \end{aligned}$$
(ii) The discrete divergence of the periodic unit flux $\widetilde{J}_P: \mathcal {E}\rightarrow \mathbb {R}$ is given by
  4.5 $$\begin{aligned} {{\,\mathrm{\text {\textsf{div}}}\,}}\widetilde{J}_P(x) = {\textbf{1}}_{\{(x_0)_\textsf{v}\}}(x_\textsf{v}) - {\textbf{1}}_{\{(x_m)_\textsf{v}\}}(x_\textsf{v}), \quad x \in \mathcal {X}. \end{aligned}$$
  In particular, ${{\,\mathrm{\text {\textsf{div}}}\,}}\widetilde{J}_P \equiv 0$ iff $(x_0)_\textsf{v}= (x_m)_\textsf{v}$.
(iii) The periodic unit flux $\widetilde{J}_P: \mathcal {E}\rightarrow \mathbb {R}$ satisfies ${{\,\mathrm{\textsf{Eff}}\,}}(\widetilde{J}_P) = (x_m)_\textsf{z}- (x_0)_\textsf{z}$.
(iv) For every $j \in \mathbb {R}^d$ we have ${{\,\mathrm{{\textsf{Rep}}}\,}}(j) \ne \emptyset$.

#### Proof

*(i)* is straightforward to check, and *(ii)* is a direct consequence.

To prove *(iii)*, we use the definition of $\widetilde{J}_P$ to obtain

$$\begin{aligned} \sum _{ (x,y) \in \mathcal {E}^Q} \widetilde{J}_P(x,y) \big ( y_\textsf{z}- x_\textsf{z}\big )&= \sum _{ (x,y) \in \mathcal {E}^Q} \sum _{z \in \mathbb {Z}^d} J_P( T_z x, T_z y ) \big (y_\textsf{z}- x_\textsf{z}\big ) \\ {}&= \sum _{ (x,y) \in \mathcal {E}} J_P(x,y) \big (y_\textsf{z}- x_\textsf{z}\big ). \end{aligned}$$

By construction, we have

$$\begin{aligned} \frac{1}{2} \sum _{(x,y) \in \mathcal {E}} J_P(x,y) \big (y_\textsf{z}- x_\textsf{z}\big ) = \sum _{j=1}^m (x_j)_\textsf{z}- (x_{j-1})_\textsf{z}= (x_m)_\textsf{z}- (x_0)_\textsf{z}, \end{aligned}$$

which yields the result.

For *(iv)*, taking $j = e_i$, we use the connectivity and nonemptyness of $(\mathcal {X},\mathcal {E})$ to find a simple path connecting some $(v,z) \in \mathcal {X}$ to $(v,z+e_i) \in \mathcal {X}$. The resulting $\widetilde{J}_P \in \mathbb {R}_a^\mathcal {E}$ is divergence-free by *(ii)* and ${{\,\mathrm{\textsf{Eff}}\,}}(\widetilde{J}_P)= e_i$ by *(iii)*, so that $\widetilde{J}_P \in {{\,\mathrm{{\textsf{Rep}}}\,}}(e_i)$. For a general $j=\sum _{i=1}^d j_i e_i$ we have ${{\,\mathrm{{\textsf{Rep}}}\,}}(j) \supseteq \sum _{i=1}^d j_i {{\,\mathrm{{\textsf{Rep}}}\,}}(e_i) \ne \emptyset$.

### The homogenised action

We are now in a position to define the homogenised energy density.

#### Definition 4.6

(Homogenised energy density) The *homogenised energy density* $f_{\textrm{hom}}: \mathbb {R}_+ \times \mathbb {R}^d \rightarrow \mathbb {R}\cup \{ +\infty \}$ is defined by the cell formula

4.6 $$\begin{aligned} \begin{aligned} f_{\textrm{hom}}(\rho ,j)&:= \inf \big \{ F(m,J) \ : \ (m,J) \in {{\,\mathrm{{\textsf{Rep}}}\,}}(\rho ,j) \big \}. \end{aligned} \end{aligned}$$

For $(\rho , j)\in \mathbb {R}_+ \times \mathbb {R}^d$, we say that $(m,J) \in {{\,\mathrm{{\textsf{Rep}}}\,}}(\rho ,j)$ is an *optimal representative* if $F(m,J) = f_{\textrm{hom}}(\rho ,j)$. The set of optimal representatives is denoted by

$$\begin{aligned} {{\,\mathrm{{\textsf{Rep}}}\,}}_o(\rho , j). \end{aligned}$$

In view of Lemma 4.5, the set of representatives ${{\,\mathrm{{\textsf{Rep}}}\,}}(\rho , j)$ is nonempty for every $(\rho , j)\in \mathbb {R}_+ \times \mathbb {R}^d$. The next result shows that ${{\,\mathrm{{\textsf{Rep}}}\,}}_o(\rho , j)$ is nonempty as well.

#### Lemma 4.7

(Properties of the cell formula) Let $(\rho , j)\in \mathbb {R}_+ \times \mathbb {R}^d$. If $f_\textrm{hom}(\rho ,j) < + \infty$, then the set of optimal representatives ${{\,\mathrm{{\textsf{Rep}}}\,}}_o(\rho , j)$ is nonempty, closed, and convex.

#### Proof

This follows from the coercivity of *F* and the direct method of the calculus of variations.

#### Lemma 4.8

(Properties of $f_\textrm{hom}$ and $f_\textrm{hom}^\infty$) The following properties hold:

(i) The functions $f_\textrm{hom}$ and $f_\textrm{hom}^\infty$ are lower semicontinuous and convex.
(ii) There exist constants $c > 0$ and $C < \infty$ such that, for all $\rho \ge 0$ and $j \in \mathbb {R}^d$,
  4.7 $$\begin{aligned} f_\textrm{hom}(\rho ,j) \ge c|j| - C (\rho + 1), \qquad f_\textrm{hom}^\infty (\rho , j) \ge c|j| - C \rho . \end{aligned}$$
(iii) The domain ${{\,\mathrm{\textsf{D}}\,}}(f_\textrm{hom}) \subseteq \mathbb {R}_+ \times \mathbb {R}^d$ has nonempty interior. In particular, for any pair $(m^\circ , J^\circ )$ satisfying (2.2), the element $(\rho ^\circ , j^\circ ) \in (0,\infty ) \times \mathbb {R}^d$ defined by
  4.8 $$\begin{aligned} (\rho ^\circ , j^\circ ) := \bigg ( \sum _{x \in \mathcal {X}^Q} m^\circ (x) , \frac{1}{2}\sum _{(x,y) \in \mathcal {E}^Q} J^\circ (x,y) \big ( y_\textsf{z}- x_\textsf{z}\big ) \bigg ) \end{aligned}$$
  belongs to $${{\,\mathrm{\textsf{D}}\,}}(f_\textrm{hom})^\circ $$.

#### Proof

*(i)*: The convexity of $f_\textrm{hom}$ follows from the convexity of *F* and the affinity of the constraints. Let us now prove lower semicontinuity of $f_\textrm{hom}$.

Take $(\rho , j) \in \mathbb {R}_+ \times \mathbb {R}^d$ and sequences $\{\rho _n\}_n \subseteq \mathbb {R}_+$ and $\{j_n\}_n \subseteq \mathbb {R}^d$ converging to $\rho$ and *j* respectively. Without loss of generality we may assume that $L:= \sup _n f_\textrm{hom}(\rho _n, j_n) < \infty$. By definition of $f_\textrm{hom}$, there exist $(m_n, J_n) \in {{\,\mathrm{{\textsf{Rep}}}\,}}(\rho _n, j_n)$ such that $F(m_n, J_n) \le f_\textrm{hom}(\rho _n, j_n) + \frac{1}{n}$. From the growth condition (2.1) we deduce that, for some $C < \infty$,

$$\begin{aligned} \sup _n \sum _{ x \in \mathcal {X}^Q} m_n(x) = \sup _n \rho _n< \infty \quad \text { and }\quad \sup _n \sum _{(x,y) \in \mathcal {E}^Q} |J_n(x,y)| \le C\big ( 1 + L + \sup _n r_n\big ) < \infty . \end{aligned}$$

From the Bolzano–Weierstrass theorem we infer subsequential convergence of $\{(m_n, J_n)\}_n$ to some $\mathbb {Z}^d$-periodic pair $(m, J) \in \mathbb {R}_+^\mathcal {X}\times \mathbb {R}^\mathcal {E}$. Therefore, by lower semicontinuity of *F*, it follows that

4.9 $$\begin{aligned} F(m, J) \le \liminf _{n \rightarrow \infty } F(m_n, J_n) \le \liminf _{n \rightarrow \infty } f_\textrm{hom}(\rho _n, j_n) \end{aligned}$$

Since $(m,J) \in {{\,\mathrm{{\textsf{Rep}}}\,}}(\rho ,j)$, we have $f_\textrm{hom}(\rho , j) \le F(m, J)$, which yields the desired result. Convexity and lower semicontinuity of $f_\textrm{hom}^\infty$ follow from the definition, see [2, Section 2.6].

*(ii)* Take $\rho \in \mathbb {R}_+$ and $j \in \mathbb {R}^d$. If $f_\textrm{hom}(\rho , j) = + \infty$, the assertion is trivial, so we assume that $f_\textrm{hom}(\rho , j) < + \infty$. Then there exists a competitor $(m, J) \in {{\,\mathrm{{\textsf{Rep}}}\,}}(\rho ,j)$ such that $F(m,J) \le f_\textrm{hom}(\rho ,j) + 1$. The growth condition (2.1) asserts that

$$\begin{aligned} F(m,J)&\ge c \sum _{(x,y) \in \mathcal {E}^Q} |J(x,y)| - C \sum _{x \in \mathcal {X}^Q} m(x) - C \end{aligned}$$

Therefore, the claim follows from the fact that

$$\begin{aligned} R_0 \sum _{(x,y) \in \mathcal {E}^Q} |J(x,y)| \ge |j| \quad \text { and }\quad \sum _{x \in \mathcal {X}^Q} m(x) = r, \end{aligned}$$

where $$R_0 = \max _{(x,y) \in \mathcal {E}} | x_\textsf{z}- y_\textsf{z}|_{\ell _\infty ^d} $$.

*(iii)*: Let $( m^\circ , J^\circ ) \in {{\,\mathrm{\textsf{D}}\,}}(F)^\circ$ satisfy Assumption 2.3, and define $(\rho ^\circ , j^\circ ) \in (0,\infty ) \times \mathbb {R}^d$ by (4.8). For $i = 1, \ldots , d$, let $e_i$ be the coordinate unit vector. Using Lemma 4.5*(*iv) we take $J^i \in {{\,\mathrm{{\textsf{Rep}}}\,}}(e_i)$. For $\alpha \in \mathbb {R}$ with $|\alpha |$ sufficiently small, and $\beta = \sum _{i=1}^d \beta _i e_i \in \mathbb {R}^d$ we define

$$\begin{aligned} m_\alpha (x)&:= m^\circ (x) + \frac{\alpha }{\#(\mathcal {X}^Q)}{} & {} x \in \mathcal {X}, \\ J_\beta (x,y)&:= J^\circ (x,y) + \sum _{i=1}^d \beta _i J^i(x,y){} & {} (x,y) \in \mathcal {E}. \end{aligned}$$

It follows that $(m_\alpha , J_\beta ) \in {{\,\mathrm{{\textsf{Rep}}}\,}}(\rho ^\circ + \alpha , j^\circ + \beta )$, and therefore, $f_\textrm{hom}(\rho ^\circ + \alpha , j^\circ + \beta ) \le F(m_\alpha , J_\beta )$. By Assumption 2.3, the right-hand side is finite for $|\alpha | + |\beta |$ sufficiently small. This yields the result.

The homogenised action $\mathbb {A}^\mathcal {I}_\textrm{hom}$ can now be defined by taking $f = f_\textrm{hom}$ in Definition 3.10.

### Embedding of solutions to the discrete continuity equation

For $\varepsilon > 0$ and $z \in \mathbb {Z}^d$ (or more generally, for $z \in \mathbb {R}^d$) let $Q_\varepsilon ^z:= \varepsilon z + [0, \varepsilon )^d \subseteq \mathbb {T}^d$ denote the cube of side-length $\varepsilon$ based at $\varepsilon z$. For $m \in \mathbb {R}_+^{\mathcal {X}_\varepsilon }$ and $J \in \mathbb {R}_a^{\mathcal {E}_\varepsilon }$ we define $\iota _\varepsilon m \in \mathcal {M}_+(\mathbb {T}^d)$ and $\iota _\varepsilon J \in \mathcal {M}^d(\mathbb {T}^d)$ by

4.10a $$\begin{aligned} \iota _\varepsilon m&:= \varepsilon ^{-d} \sum _{x \in \mathcal {X}_\varepsilon } m(x) \mathscr {L}^d|_{Q_\varepsilon ^{x_\textsf{z}}}, \end{aligned}$$

4.10b $$\begin{aligned} \iota _\varepsilon J&:= \varepsilon ^{-d+1} \sum _{(x,y) \in \mathcal {E}_\varepsilon } \frac{J(x,y)}{2} \bigg (\int _0^1 \mathscr {L}^d|_{ Q_\varepsilon ^{(1-s)x_\textsf{z}+ s y_\textsf{z}}} \, \textrm{d}s\bigg ) (y_\textsf{z}- x_\textsf{z}), \end{aligned}$$

The embeddings (4.10) are chosen to ensure that solutions to the discrete continuity equation are mapped to solutions to the continuous continuity equation, as the following result shows.

#### Lemma 4.9

Let $({\pmb {m}}, \pmb {J}) \in \mathcal{C}\mathcal{E}_\varepsilon ^\mathcal {I}$ solve the discrete continuity equation and define $\mu _t = \iota _\varepsilon m_t$ and $\nu _t = \iota _\varepsilon J_t$. Then $({\varvec{\mu }}, {\varvec{\nu }})$ solves the continuity equation (i.e., $({\varvec{\mu }}, {\varvec{\nu }}) \in \mathbb{C}\mathbb{E}^\mathcal {I}$).

#### Proof

Let $\varphi : \mathcal {I}\times \mathbb {T}^d \rightarrow \mathbb {R}$ be smooth with compact support. Then:

$$\begin{aligned}&\int _\mathcal {I}\int _{\mathbb {T}^d} \nabla \varphi \cdot \, \textrm{d}\nu _t \, \textrm{d}t\\&\quad = \frac{1}{2 \varepsilon ^d} \sum _{(x,y) \in \mathcal {E}_\varepsilon } \int _\mathcal {I}J_t(x,y) \int _0^1 \int _{Q_\varepsilon ^{(1-s) x_\textsf{z}+ s y_\textsf{z}}} \nabla \varphi (t,x) \cdot \varepsilon (y_\textsf{z}- x_\textsf{z}) \, \textrm{d}\mathscr {L}^d \, \textrm{d}s \, \textrm{d}t\\&\quad = \frac{1}{2 \varepsilon ^d} \sum _{(x,y) \in \mathcal {E}_\varepsilon } \int _\mathcal {I}J_t(x,y) \int _0^1 \partial _s \bigg ( \int _{Q_\varepsilon ^{(1-s) x_\textsf{z}+ s y_\textsf{z}}} \varphi \, \textrm{d}\mathscr {L}^d \bigg ) \, \textrm{d}s \, \textrm{d}t\\&\quad = \frac{1}{2 \varepsilon ^d} \sum _{(x,y) \in \mathcal {E}_\varepsilon } \int _\mathcal {I}J_t(x,y) \bigg ( \int _{Q_\varepsilon ^{y_\textsf{z}}} \varphi \, \textrm{d}\mathscr {L}^d - \int _{Q_\varepsilon ^{x_\textsf{z}}} \varphi \, \textrm{d}\mathscr {L}^d \bigg ) \, \textrm{d}t. \end{aligned}$$

On the other hand, the discrete continuity equation yields

$$\begin{aligned} \int _\mathcal {I}\int _{\mathbb {T}^d} \partial _t \varphi \, \textrm{d}\mu _t \, \textrm{d}t&= \frac{1}{\varepsilon ^{d}} \sum _{x \in \mathcal {X}_\varepsilon } \int _\mathcal {I}m_t(x) \partial _t \bigg (\int _{Q_\varepsilon ^{x_\textsf{z}}} \varphi \, \textrm{d}\mathscr {L}^d \bigg ) \, \textrm{d}t \\ {}&= \frac{1}{2\varepsilon ^d} \sum _{(x,y) \in \mathcal {E}_\varepsilon } \int _\mathcal {I}J_t(x,y) \bigg (\int _{Q_\varepsilon ^{x_\textsf{z}}} \varphi \, \textrm{d}\mathscr {L}^d - \int _{Q_\varepsilon ^{y_\textsf{z}}} \varphi \, \textrm{d}\mathscr {L}^d \bigg ) \, \textrm{d}t. \end{aligned}$$

Comparing both expressions, we obtain the desired identity $\partial _t {\varvec{\mu }}+ \nabla \cdot {\varvec{\nu }}= 0$ in the sense of distributions.

The following result provides a useful bound for the norm of the embedded flux.

#### Lemma 4.10

For $J \in \mathbb {R}_a^{\mathcal {E}_\varepsilon }$ we have

$$\begin{aligned} |\iota _\varepsilon J|(\mathbb {T}^d) \le \frac{\varepsilon R_0 \sqrt{d}}{2} \sum _{(x,y) \in \mathcal {E}_\varepsilon } |J(x,y)|. \end{aligned}$$

#### Proof

This follows immediately from (4.11), since $\mathscr {L}^d \big (Q_\varepsilon ^{(1-s)x_\textsf{z}+ s y_\textsf{z}}\big ) = \varepsilon ^d$ and $|y_\textsf{z}- x_\textsf{z}| \le R_0 \sqrt{d}$ for $(x,y) \in \mathcal {E}_\varepsilon$.

Note that both measures in (4.10) are absolutely continuous with respect to the Lebesgue measure. The next result provides an explicit expression for the density of the momentum field. Recall the definition of the shifting operators $\sigma _\varepsilon ^{\bar{z}}$ in (2.5).

#### Lemma 4.11

(Density of the embedded flux) Fix $\varepsilon <\frac{1}{2R_0}$. For $J \in \mathbb {R}_a^{\mathcal {E}_\varepsilon }$ we have $\iota _\varepsilon J = j_\varepsilon \mathscr {L}^d$ where $j_\varepsilon : \mathbb {T}^d\rightarrow \mathbb {R}^d$ is given by

4.11 $$\begin{aligned} j_\varepsilon (u)&= \varepsilon ^{-d+1} \sum _{z \in \mathbb {Z}_\varepsilon ^d} \chi _{Q_\varepsilon ^z}(u) \Bigg (\frac{1}{2} \sum _{ \begin{array}{c} (x, y) \in \mathcal {E}_\varepsilon \\ x_\textsf{z}= z \end{array} } J_u(x, y) \big ( y_\textsf{z}- x_\textsf{z}\big ) \Bigg ) \quad \text {for } u \in \mathbb {T}^d. \end{aligned}$$

Here, $J_u(x, y)$ is a convex combination of $\big \{ \sigma _\varepsilon ^{\bar{z}} J (x,y )\big \}_{{\bar{z}}\in \mathbb {Z}_\varepsilon ^d}$, i.e.,

$$\begin{aligned} J_u(x, y)&= \sum _{\bar{z} \in \mathbb {Z}_\varepsilon ^d} \lambda _u^{\varepsilon ,\bar{z}}(x,y) \sigma _\varepsilon ^{\bar{z}} J (x,y ), \end{aligned}$$

where $\lambda _u^{\varepsilon , {\bar{z}}}(x,y) \ge 0$ and $\sum _{{\bar{z}} \in \mathbb {Z}_\varepsilon ^d} \lambda _u^{\varepsilon ,{\bar{z}}}(x,y) = 1$. Moreover,

4.12 $$\begin{aligned} \lambda _u^{\varepsilon , {\bar{z}}}(x,y) = 0 \quad \text {whenever} \ u \in Q_\varepsilon ^{x_\textsf{z}}, \, |{\bar{z}}|_\infty > R_0 + 1. \end{aligned}$$

#### Proof

Fix $\varepsilon < \frac{1}{2R_0}$, let $z \in \mathbb {Z}_\varepsilon ^d$ and $u \in Q_\varepsilon ^z$. We have

$$\begin{aligned} j_\varepsilon (u)&= \varepsilon ^{-d+1} \sum _{(x,y) \in \mathcal {E}_\varepsilon } \frac{J(x,y)}{2} \bigg (\int _0^1 \chi _{Q_\varepsilon ^{(1-s)x_\textsf{z}+ s y_\textsf{z}}}(u) \, \textrm{d}s\bigg ) (y_\textsf{z}- x_\textsf{z}) \\ {}&= \varepsilon ^{-d+1} \sum _{ \begin{array}{c} (x, y) \in \mathcal {E}_\varepsilon \\ x_\textsf{z}= z \end{array} } \sum _{\bar{z} \in \mathbb {Z}_\varepsilon ^d} \frac{\sigma _\varepsilon ^{\bar{z}} J (x,y)}{2} \bigg (\int _0^1 \chi _{Q_\varepsilon ^{\bar{z} + (1-s)x_\textsf{z}+ s y_\textsf{z}}}(u) \, \textrm{d}s\bigg ) (y_\textsf{z}- x_\textsf{z}), \end{aligned}$$

which is the desired form (4.11) with

$$\begin{aligned} \lambda _u^{\varepsilon , {\bar{z}}}(x,y) = \bigg (\int _0^1 \chi _{Q_\varepsilon ^{\bar{z} + (1-s)x_\textsf{z}+ s y_\textsf{z}}}(u) \, \textrm{d}s\bigg ) \end{aligned}$$

for $(x, y) \in \mathcal {E}_\varepsilon$ with $x_\textsf{z}= z$. Since the family of cubes $\big \{ Q_\varepsilon ^{\bar{z} + sy_\textsf{z}+(1-s)x_\textsf{z}} \big \}_{\bar{z} \in \mathbb {Z}_\varepsilon ^d}$ is a partition of $\mathbb {T}^d$, it follows that $\sum _{{\bar{z}} \in \mathbb {Z}_\varepsilon ^d} \lambda _u^{\varepsilon ,{\bar{z}}}(x,y) = 1$.

To prove the final claim, let $(x, y) \in \mathcal {E}_\varepsilon$ with $x_\textsf{z}= z$ as above and take $\bar{z} \in \mathbb {Z}_\varepsilon ^d$ with $|{\bar{z}}|_\infty > R_0 + 1$. Since $|x_\textsf{z}- y_\textsf{z}| \le R_0$, the triangle inequality yields

$$\begin{aligned} \big \Vert \big ( \bar{z} + s y_\textsf{z}+ (1-s) x_\textsf{z}\big ) - x_\textsf{z}\big \Vert _\infty \ge \Vert \bar{z} \Vert _\infty - (1-s) \Vert y_\textsf{z}- x_\textsf{z}\Vert _\infty > 1, \end{aligned}$$

for $s \in [0,1]$. Therefore, $u \in Q_\varepsilon ^z$ implies $\chi _{Q_\varepsilon ^{\bar{z} + (1-s)x_\textsf{z}+ s y_\textsf{z}}}(u) = 0$, hence $\lambda _u^{\varepsilon , {\bar{z}}}(x,y) = 0$ as desired.

## Main results

In this section we present the main result of this paper, which asserts that the discrete action functionals $\mathcal {A}_\varepsilon$ converge to a continuous action functional $\mathbb {A}= \mathbb {A}_\textrm{hom}$ with the nontrivial homogenised action density function $f = f_\textrm{hom}$ defined in Sect. 4.

### Main convergence result

We are now ready to state our main result. We use the embedding $\iota _\varepsilon : \mathbb {R}_+^{\mathcal {X}_\varepsilon }\rightarrow \mathcal {M}_+(\mathbb {T}^d)$ defined in (4.10a). The proof of this result is given in Sects. 7 and 8.

#### Theorem 5.1

($\Gamma$-convergence) Let $(\mathcal {X}, \mathcal {E})$ be a locally finite and $\mathbb {Z}^d$-periodic connected graph of bounded degree (see Assumption 2.1). Let $F: \mathbb {R}_+^\mathcal {X}\times \mathbb {R}_a^\mathcal {E}\rightarrow \mathbb {R}\cup \{+\infty \}$ be a cost function satisfying Assumption 2.3. Then the functionals $\mathcal {A}^\mathcal {I}_\varepsilon$ $\Gamma$-converge to $\mathbb {A}^\mathcal {I}_{\textrm{hom}}$ as $\varepsilon \rightarrow 0$ with respect to the weak (and vague) topology. More precisely:

(i) (**liminf inequality**) Let ${\varvec{\mu }}\in \mathcal {M}_+(\mathcal {I}\times \mathbb {T}^d)$. For any sequence of curves $\{{\pmb {m}}^\varepsilon \}_{\varepsilon }$ with ${\pmb {m}}^\varepsilon = (m_t^\varepsilon )_{t \in \mathcal {I}} \subseteq \mathbb {R}_+^{\mathcal {X}_\varepsilon }$ such that $\iota _\varepsilon {\pmb {m}}^\varepsilon \rightarrow {\varvec{\mu }}$ vaguely in $\mathcal {M}_+(\mathcal {I}\times \mathbb {T}^d)$ as $\varepsilon \rightarrow 0$, we have the lower bound
  5.1 $$\begin{aligned} \liminf _{\varepsilon \rightarrow 0} \mathcal {A}^\mathcal {I}_\varepsilon ({\pmb {m}}^\varepsilon ) \ge \mathbb {A}^\mathcal {I}_{\textrm{hom}}({\varvec{\mu }}). \end{aligned}$$
(ii) (**limsup inequality**) For any ${\varvec{\mu }}\in \mathcal {M}_+(\mathcal {I}\times \mathbb {T}^d)$ there exists a sequence of curves $\{{\pmb {m}}^\varepsilon \}_{\varepsilon }$ with ${\pmb {m}}^\varepsilon = (m_t^\varepsilon )_{t \in \mathcal {I}} \subseteq \mathbb {R}_+^{\mathcal {X}_\varepsilon }$ such that $\iota _\varepsilon {\pmb {m}}^\varepsilon \rightarrow {\varvec{\mu }}$ weakly in $\mathcal {M}_+(\mathcal {I}\times \mathbb {T}^d)$ as $\varepsilon \rightarrow 0$, and we have the upper bound
  5.2 $$\begin{aligned} \limsup _{\varepsilon \rightarrow 0} \mathcal {A}^\mathcal {I}_\varepsilon ({\pmb {m}}^\varepsilon ) \le \mathbb {A}^\mathcal {I}_{\textrm{hom}}({\varvec{\mu }}). \end{aligned}$$

#### Remark 5.2

(Necessity of the interior domain condition) Assumption 2.3 is crucial in order to obtain the $\Gamma$-convergence of the discrete energies. Too see this, let us consider the one-dimensional graph $\mathcal {X}=\mathbb {Z}$ and the edge-based cost associated with

$$\begin{aligned} F_{xy}(m(x),m(y),J(x,y)) := {\left\{ \begin{array}{ll} \frac{J(x,y)^2}{m(x)} &{} \text {if } m(x)=m(y) \ne 0 ,\\ 0 &{} \text {if } J(x,y)=m(x)=m(y)=0 ,\\ \infty &{} \text {otherwise}. \end{array}\right. } \end{aligned}$$

Clearly *F* satisfies conditions $(a)-(c)$ from Assumption 2.3, but (*d*) fails to hold. The constraint $m(x)=m(y)$ on neighbouring $x,y \in \mathcal {X}$ forces every ${\pmb {m}}: \mathcal {I}\rightarrow \mathbb {R}_+^{\mathcal {X}_\varepsilon }$ with $\mathcal {A}_\varepsilon ({\pmb {m}})<\infty$ to be constant in space (and hence in time, by mass preservation). Therefore, the $\Gamma$-limit of the $\mathcal {A}_\varepsilon$ is finite only on constant measures ${\varvec{\mu }}= \alpha \mathscr {L}^{d+1}$, with $\alpha \in \mathbb {R}_+$. On the other hand, we have^3^ that $f_\textrm{hom}(\rho ,j)=\frac{|j|^2}{\rho }$, which corresponds to the $\mathbb {W}_2$ action on the line.

It is interesting to note that if the constraint “$m(x) = m(y)$” is replaced by a weaker one of the form “$|m(x)-m(y)|\le \delta$ ” for some $\delta >0$, then all the assumptions are satisfied and our theorem can be applied. Intuitively speaking, the constraint which forces admissible curves to be constant is replaced by a constraint that merely forces admissible curves to be Lipschitz; in this case the limit coincides with the $\mathbb {W}_2$ action.

See also Sect. 9.2 for a general treatment of the cell formula on the integer lattice $\mathcal {X}=\mathbb {Z}^d$.

### Scaling limits of Wasserstein transport problems

For $1 \le p < \infty$, recall that the energy density associated to the Wasserstein metric $\mathbb {W}_p$ on $\mathbb {R}^d$ is given by $f(\rho , j) = \frac{|j|^p}{\rho ^{p-1}}$. This function satisfies the scaling relations $f(\lambda \rho , \lambda j) = \lambda f(\rho , j)$ and $f(\rho , \lambda j) = |\lambda |^p f(\rho , j)$ for $\lambda \in \mathbb {R}$.

In discrete approximations of $\mathbb {W}_p$ on a periodic graph $(\mathcal {X},\mathcal {E})$, it is reasonable to assume analogous scaling relations for the function *F*, namely $F(\lambda m, \lambda J) = \lambda F(m, J)$ and $F(m, \lambda J) = |\lambda |^p F(m, J)$. The next result shows that if such scaling relations are imposed, we always obtain convergence to $\mathbb {W}_p$ with respect to some norm on $\mathbb {R}^d$. This norm does not have to be Hilbertian (even in the case $p=2$) and is characterised by the cell problem (4.6).

#### Corollary 5.3

Let $1 \le p < \infty$, and suppose that *F* has the following scaling properties for $m \in \mathbb {R}_+^\mathcal {X}$ and $j \in \mathbb {R}_a^\mathcal {E}$:

(i) $$F(\lambda m, \lambda J) = \lambda F(m, J)$$ for all $$\lambda \ge 0$$;
(ii) $F(m, \lambda J) = |\lambda |^p F(m, J)$ for all $\lambda \in \mathbb {R}$.

Then $f_\textrm{hom}(\rho , j) = \frac{\Vert j \Vert ^p}{\rho ^{p-1}}$ for some norm $\Vert \cdot \Vert$ on $\mathbb {R}^d$.

#### Proof

Fix $\rho > 0$ and $j \in \mathbb {R}^d$. The scaling assumptions imply that

5.3 $$\begin{aligned} f_\textrm{hom}(\lambda \rho , \lambda j) = \lambda f_\textrm{hom}(\rho , j) \quad \text { and }\quad f_\textrm{hom}(\rho , \lambda j) = |\lambda |^p f_\textrm{hom}(\rho , j). \end{aligned}$$

Consequently,

$$\begin{aligned} f_\textrm{hom}(\rho , j) = \rho f_\textrm{hom}(1, j/\rho ) = \frac{f_\textrm{hom}(1, j)}{\rho ^{p-1}}. \end{aligned}$$

We claim that $f_\textrm{hom}(1, j) > 0$ whenever $j \ne 0$. Indeed, it follows from (4.7) that $f_\textrm{hom}(1, j) > 0$ whenever |*j*| is sufficiently large. By homogeneity (5.3), the same holds for every $j \ne 0$. It also follows from (5.3) that $f_\textrm{hom}(1, 0) = 0$.

We can thus define $\Vert j\Vert := f_\textrm{hom}(1,j)^{1/p} \in [0,\infty )$. In view of the previous comments, we have $\Vert 0\Vert = 0$ and $\Vert j\Vert > 0$ for all $j \in \mathbb {R}^d \setminus \{0\}$. The homogeneity (5.3) implies that $\Vert \lambda j\Vert = |\lambda |\, \Vert j \Vert$ for $j \in \mathbb {R}^d$ and $\lambda \in \mathbb {R}$.

It remains to show the triangle inequality $\Vert j_1 + j_2 \Vert \le \Vert j_1\Vert + \Vert j_2\Vert$ for $j_1, j_2 \in \mathbb {R}^d$. Without loss of generality we assume that $\Vert j_1\Vert + \Vert j_2\Vert > 0$. For $\lambda \in (0,1)$, the convexity of $f_\textrm{hom}$ (see Lemma 4.8) and the homogeneity (5.3) yield

$$\begin{aligned} f_\textrm{hom}(1,j_1 + j_2) \le (1-\lambda ) f_\textrm{hom}\Big (1, \frac{j_1}{1-\lambda } \Big ) + \lambda f_\textrm{hom}\Big (1, \frac{j_2}{\lambda } \Big ) = \frac{f_\textrm{hom}(1, j_1)}{(1 - \lambda )^{p-1}} + \frac{f_\textrm{hom}(1, j_2)}{\lambda ^{p-1}}. \end{aligned}$$

Substitution of $\lambda = \frac{\Vert j_2\Vert }{\Vert j_1\Vert + \Vert j_2\Vert }$ yields the triangle inequality.

### Compactness results

As we frequently need to compare measures with unequal mass in this paper, it is natural to work with the the *Kantorovich–Rubinstein norm*. This metric is closely related to the transport distance $\mathbb {W}_1$; see “Appendix 1”.

The following compactness result holds for solutions to the continuity equation with bounded action. As usual, we use the notation ${\varvec{\mu }}(\textrm{d}x, \textrm{d}t) = \mu _t(\textrm{d}x) \, \textrm{d}t$.

#### Theorem 5.4

(Compactness under linear growth) Let ${\pmb {m}}^\varepsilon :\mathcal {I}\rightarrow \mathbb {R}_+^{\mathcal {X}_\varepsilon }$ be such that

$$\begin{aligned} \sup _{\varepsilon> 0} \mathcal {A}^\mathcal {I}_\varepsilon ({\pmb {m}}^\varepsilon )< \infty \quad \text { and }\quad \sup _{\varepsilon > 0} {\pmb {m}}^\varepsilon (\mathcal {I}\times \mathcal {X}_\varepsilon ) < \infty . \end{aligned}$$

Then there exists a curve $(\mu _t)_{t \in \mathcal {I}} \in \textrm{BV}_{\textrm{KR}}(\mathcal {I}; \mathcal {M}_+(\mathbb {T}^d))$ such that, up to extracting a subsequence,

(i) $$\iota _\varepsilon {\pmb {m}}^\varepsilon \rightarrow {\varvec{\mu }}$$ weakly in $$\mathcal {M}_+(\mathcal {I}\times \mathbb {T}^d)$$;
(ii) $\iota _\varepsilon m_t^\varepsilon \rightarrow \mu _t$ weakly in $\mathcal {M}_+(\mathbb {T}^d)$ for almost every $t\in \mathcal {I}$;
(iii) $t \mapsto \mu _t(\mathbb {T}^d)$ is constant.

The proof of this result is given in Sect. 6.

Under a superlinear growth condition on the cost function *F*, the following stronger compactness result holds.

#### Assumption 5.5

(Superlinear growth) We say that *F* is of *superlinear growth* if there exists a function $\theta :[0,\infty ) \rightarrow [0,\infty )$ with $\lim _{t\rightarrow \infty } \frac{\theta (t)}{t} = \infty$ and a constant $C \in \mathbb {R}$ such that

5.4 $$\begin{aligned} F(m,J) \ge (m_0 + 1) \theta \bigg ( \frac{J_0}{m_0+1} \bigg ) - C (m_0 + 1) \end{aligned}$$

for all $m\in \mathbb {R}_+^\mathcal {X}$ and all $J\in \mathbb {R}^\mathcal {E}_a$, where

5.5 $$\begin{aligned} m_0 = \sum _{\begin{array}{c} x \in \mathcal {X}\\ |x|_{\ell _\infty ^d} \le R \end{array}} m(x) \quad \text { and }\quad J_0 = \sum _{(x,y) \in \mathcal {E}^Q} |J(x,y)|, \end{aligned}$$

with $R = \max \{R_0, R_1\}$ as in Assumption 2.3.

#### Remark 5.6

The superlinear growth condition (5.4) implies the linear growth condition (2.1). To see this, suppose that *F* has superlinear growth. Let $v_0 > 0$ be such that $\theta (v) \ge v$ for $v\ge v_0$. If $\frac{J_0}{m_0 + 1} \ge v_0$, we have

5.6 $$\begin{aligned} F(m,J) \ge (m_0 + 1) \theta \bigg ( \frac{J_0}{m_0+1} \bigg ) - C (m_0 + 1) \ge J_0 - C (m_0 + 1). \end{aligned}$$

On the other hand, if $\frac{J_0}{m_0+1} < v_0$, the nonnegativity of $\theta$ implies that

5.7 $$\begin{aligned} F(m,J) \ge - C (m_0 + 1) \ge \frac{C}{v_0} J_0 - 2C (m_0 + 1). \end{aligned}$$

Combining (5.6) and (5.7), we have

$$\begin{aligned} F(m,J) \ge \min \bigg \{ 1, \frac{C}{v_0} \bigg \} J_0 - 2C (m_0 + 1), \end{aligned}$$

which is of the desired form (2.1).

#### Example 5.7

The edge-based costs

$$\begin{aligned} F(m,J) = \frac{1}{2}\sum _{(x,y)\in \mathcal {E}^Q}| J(x,y)|^p \end{aligned}$$

have superlinear growth if and only if $1<p<\infty$ (with $\theta (t) = c t^p$ and $c = |\mathcal {E}^Q|^{1-p}$). Indeed,

$$\begin{aligned} 2F(m,J) = \sum _{(x,y) \in \mathcal {E}^Q} |J(x,y)|^p \ge c J_0^p \ge c \frac{J_0^p}{(m_0+1)^{p-1}} = c (m_0+1) \theta \left( \frac{J_0}{m_0+1}\right) . \end{aligned}$$

#### Example 5.8

The functions (2.3) arising in discretisation of *p*-Wasserstein distances have superlinear growth if and only if $p > 1$ (with $\theta (t) = t^p$).

To see this, consider the function $G(\alpha ,\beta ,\gamma ):= \frac{1}{2} \frac{|\gamma |^p}{\Lambda (\alpha ,\beta )^{p-1}}$. Since *G* is convex, non increasing in $(\alpha ,\beta )$, and positively one-homogeneous, we obtain

$$\begin{aligned} F(m,J)&= \sum _{(x,y) \in \mathcal {E}^Q} G\big (q_{xy}m(x), q_{yx}m(y), J(x,y)\big ) \\ {}&\ge G\left( \sum _{(x,y)\in \mathcal {E}^Q} q_{xy}m(x), \sum _{(x,y)\in \mathcal {E}^Q} q_{yx}m(y), \sum _{(x,y)\in \mathcal {E}^Q} |J(x,y)| \right) \\ {}&\ge c G(m_0,m_0,J_0) \ge \frac{c}{2} \frac{J_0^p}{(m_0+1)^{p-1}} = \frac{c}{2} (m_0+1) \theta \left( \frac{J_0}{m_0+1}\right) , \end{aligned}$$

where $c>0$ depends on *R*, the maximum degree and the weights $q_{xy}$.

#### Theorem 5.9

(Compactness under superlinear growth) Suppose that Assumption 5.5 holds. Let ${\pmb {m}}^\varepsilon : \mathcal {I}\rightarrow \mathbb {R}_+^{\mathcal {X}_\varepsilon }$ be such that

$$\begin{aligned} \sup _{\varepsilon> 0} \mathcal {A}^\mathcal {I}_\varepsilon ({\pmb {m}}^\varepsilon )< \infty \quad \text { and }\quad \sup _{\varepsilon > 0} {\pmb {m}}^\varepsilon (\mathcal {I}\times \mathcal {X}_\varepsilon ) < \infty . \end{aligned}$$

Then there exists a curve $(\mu _t)_{t \in \mathcal {I}} \in W_\textrm{KR}^{1,1}(\mathcal {I}; \mathcal {M}_+(\mathbb {T}^d))$ such that, up to extracting a subsequence,

(i) $$\iota _\varepsilon {\pmb {m}}^\varepsilon \rightarrow {\varvec{\mu }}$$ weakly in $$\mathcal {M}_+(\mathcal {I}\times \mathbb {T}^d)$$;
(ii) $\Vert \iota _\varepsilon m_t^\varepsilon - \mu _t\Vert _{\textrm{KR}(\mathbb {T}^d)} \rightarrow 0$ uniformly for $t \in \mathcal {I}$;
(iii) $t \mapsto \mu _t(\mathbb {T}^d)$ is constant.

This is proven in Sect. 6.2.

Note that curve $t \mapsto \mu _t \in W_\textrm{KR}^{1,1}(\mathcal {I}; \mathcal {M}_+(\mathbb {T}^d))$ can be continuously extended to $\overline{\mathcal {I}}$. Therefore, it is meaningful to assign boundary values to these curves.

### Result with boundary conditions

Under Assumption 5.5, we are able to obtain the following result on the convergence of dynamical optimal transport problems. Fix $\mathcal {I}=(a,b)\subset \mathbb {R}$ an open interval. Define for $m^a,m^b\in \mathbb {R}_+^{\mathcal {X}_\varepsilon }$ with $m^a(\mathcal {X}_\varepsilon ) = m^b(\mathcal {X}_\varepsilon )$ the minimal action as

5.8 $$\begin{aligned} \mathcal{M}\mathcal{A}_\varepsilon ^\mathcal {I}(m^a,m^b) := \inf \left\{ \mathcal {A}_\varepsilon ^\mathcal {I}(m)\,:\,m_a = m^a, m_b = m^b) \right\} . \end{aligned}$$

Similarly, define the minimal homogenised action for $\mu ^a,\mu ^b\in \mathcal {M}_+(\mathbb {T}^d)$ with $\mu ^a(\mathbb {T}^d) = \mu ^b(\mathbb {T}^d)$ as

5.9 $$\begin{aligned} \mathbb{M}\mathbb{A}_\textrm{hom}^\mathcal {I}(\mu ^a,\mu ^b) := \inf \left\{ \mathbb {A}_\textrm{hom}^\mathcal {I}(\mu )\,:\,\mu _a = \mu ^a, \mu _b = \mu ^b)\right\} . \end{aligned}$$

Note that in general, both $\mathbb{M}\mathbb{A}_\textrm{hom}^\mathcal {I}$ and $\mathcal{M}\mathcal{A}_\varepsilon ^\mathcal {I}$ may be infinite even if the two measures have equal mass. Here, the values $\mu _a$ and $\mu _b$ are well-defined under Assumption 5.5 by Theorem 5.9. Under linear growth, $\mu _a$ and $\mu _b$ can still be defined using the trace theorem in $\textrm{BV}$, but we cannot prove the following statement in that case (see also Remark 6.2). We prove this in Sect. 6.3.

#### Theorem 5.10

($\Gamma$-convergence of the minimal actions) Assume that Assumption 5.5 holds. Then the minimal actions $\mathcal{M}\mathcal{A}_\varepsilon ^\mathcal {I}$ $\Gamma$-converge to $\mathbb{M}\mathbb{A}_{\textrm{hom}}^\mathcal {I}$ in the weak topology of $\mathcal {M}_+(\mathbb {T}^d) \times \mathcal {M}_+(\mathbb {T}^d)$. Precisely:

(i) For any sequences $m_\varepsilon ^a$, $m_\varepsilon ^b \in \mathbb {R}_+^{\mathcal {X}_\varepsilon }$ such that $\iota _\varepsilon m_\varepsilon ^i \rightarrow \mu ^i$ weakly in $\mathcal {M}_+(\mathbb {T}^d)$ as $\varepsilon \rightarrow 0$ for $i=a,b$, we have
  5.10 $$\begin{aligned} \liminf _{\varepsilon \rightarrow 0} \mathcal{M}\mathcal{A}_\varepsilon ^\mathcal {I}(m^a_\varepsilon , m^b_\varepsilon ) \ge \mathbb{M}\mathbb{A}_{\textrm{hom}}^\mathcal {I}(\mu ^a,\mu ^b). \end{aligned}$$
(ii) For any $(\mu ^a,\mu ^b) \in \mathcal {M}_+(\mathbb {T}^d) \times \mathcal {M}_+(\mathbb {T}^d)$, there exist two sequences $m_\varepsilon ^a, m_\varepsilon ^b \in \mathbb {R}_+^{\mathcal {X}_\varepsilon }$ such that $\iota _\varepsilon m_\varepsilon ^i \rightarrow \mu ^i$ weakly in $\mathcal {M}_+(\mathbb {T}^d)$ as $\varepsilon \rightarrow 0$ for $i=a,b$ and
  5.11 $$\begin{aligned} \limsup _{\varepsilon \rightarrow 0} \mathcal{M}\mathcal{A}_\varepsilon ^\mathcal {I}(m^a_\varepsilon , m^b_\varepsilon ) \le \mathbb{M}\mathbb{A}_{\textrm{hom}}^\mathcal {I}(\mu ^a,\mu ^b). \end{aligned}$$

## Proof of compactness and convergence of minimal actions

This section is divided into three sub-parts: in the first one, we prove the general compactness result Theorem 5.4, which is valid under the linear growth assumption 2.3.

In the second and third part, we assume the stronger superlinear growth condition 5.5 and prove the improved compactness result Theorem 5.9 and the convergence results for the problems with boundary data, i.e. Theorem 5.10.

### Compactness under linear growth

The only assumption here is the linear growth condition 2.3.

#### Proof of Theorem 5.4

For $\varepsilon > 0$, let ${\pmb {m}}^\varepsilon : \mathcal {I}\rightarrow \mathbb {R}_+^{\mathcal {X}_\varepsilon }$ be a curve such that

6.1 $$\begin{aligned} \sup _{\varepsilon> 0} \mathcal {A}^\mathcal {I}_\varepsilon ({\pmb {m}}^\varepsilon )< \infty \quad \text { and }\quad \sup _{\varepsilon > 0} {\pmb {m}}^\varepsilon (\mathcal {I}\times \mathcal {X}_\varepsilon ) < \infty . \end{aligned}$$

We can find a solution to the discrete continuity equation $({\pmb {m}}^\varepsilon , \pmb {J}^\varepsilon ) \in \mathcal{C}\mathcal{E}_\varepsilon ^\mathcal {I}$, such that

$$\begin{aligned} \sup _{\varepsilon > 0} \mathcal {A}_\varepsilon ^\mathcal {I}({\pmb {m}}^\varepsilon , \pmb {J}^\varepsilon ) < \infty . \end{aligned}$$

Set $(\mu _t^\varepsilon , \nu _t^\varepsilon ):= (\iota _\varepsilon m_t^\varepsilon , \iota _\varepsilon J_t^\varepsilon )$, where $\iota _\varepsilon$ is defined in (4.10). Lemma 4.9 implies that $({\varvec{\mu }}^\varepsilon , {\varvec{\nu }}^\varepsilon ) \in \mathbb{C}\mathbb{E}^\mathcal {I}$ for every $\varepsilon >0$.

Using Lemma 4.10, the growth condition (2.1), and the bounds (6.1) on the masses and the action, we infer that

6.2 $$\begin{aligned} \sup _{\varepsilon> 0 } | {\varvec{\nu }}^\varepsilon | \big ( \mathcal {I}\times \mathbb {T}^d\big ) \le \frac{R_0 \sqrt{d}}{2} \sup _{\varepsilon > 0} \varepsilon \int _\mathcal {I}\sum _{(x,y)\in \mathcal {E}_\varepsilon } |J_t^\varepsilon (x,y)| \, \textrm{d}t < \infty . \end{aligned}$$

Up to extraction of a subsequence, the Banach–Alaoglu Theorem yields existence of a measure $\bar{{\varvec{\nu }}} \in \mathcal {M}^d(\overline{\mathcal {I}}\times \mathbb {T}^d)$ such that ${\varvec{\nu }}^\varepsilon \rightarrow \bar{{\varvec{\nu }}}$ weakly in $\mathcal {M}^d(\overline{\mathcal {I}}\times \mathbb {T}^d)$. It also follows that $|\bar{{\varvec{\nu }}}|(\overline{\mathcal {I}}\times \mathbb {T}^d) \le \liminf _{\varepsilon \rightarrow 0} |{\varvec{\nu }}^\varepsilon |(\mathcal {I}\times \mathbb {T}^d) < \infty$; see, e.g., [8, Theorem 8.4.7].

Furthermore, (6.1) and (6.2) imply that the $\textrm{BV}$-seminorms of ${\varvec{\mu }}^\varepsilon$ are bounded:

6.3 $$\begin{aligned} \sup _{\varepsilon> 0} \Vert {\varvec{\mu }}^\varepsilon \Vert _{\textrm{BV}_{\textrm{KR}}(\mathcal {I}; \mathcal {M}_+(\mathbb {T}^d))} \le \sup _{\varepsilon > 0} |{\varvec{\nu }}^\varepsilon |(\mathcal {I}\times \mathbb {T}^d) < \infty , \end{aligned}$$

In particular, $\sup _{\varepsilon > 0} {\varvec{\mu }}^\varepsilon (\mathcal {I}\times \mathbb {T}^d) < \infty$. Thus, by another application of the Banach–Alaoglu Theorem, there exists a measure ${\varvec{\mu }}\in \mathcal {M}_+(\overline{\mathcal {I}}\times \mathbb {T}^d)$ and a subsequence (not relabeled) such that ${\varvec{\mu }}^\varepsilon \rightarrow {\varvec{\mu }}$ weakly in $\mathcal {M}_+(\overline{\mathcal {I}}\times \mathbb {T}^d)$.

We claim that ${\varvec{\mu }}$ does not charge the boundary $(\overline{\mathcal {I}}{\setminus } \mathcal {I}) \times \mathbb {T}^d$ and that ${\varvec{\mu }}(\, \textrm{d}x, \, \textrm{d}t) = \mu _t(\textrm{d}x) \, \textrm{d}t$ for a curve $(\mu _t)_{t \in \mathcal {I}}$ of constant total mass in time. To prove the claim, write $e_1(t,x):= t$, and note that each curve $t \mapsto \mu _t^\varepsilon$ is of constant mass. Therefore, the time-marginals $(e_1)_{\#} {\varvec{\mu }}^\varepsilon \in \mathcal {M}_+(\mathcal {I})$ are constant multiples of the Lebesgue measure. Since these measures are weakly-convergent to the time-marginal $(e_1)_{\#} {\varvec{\mu }}$, it follows that the latter is also a constant multiple of the Lebesgue measure, which implies the claim. See also the proof of Lemma 3.13 for a similar discussion.

By what we just proved, ${\varvec{\mu }}$ can be identified with a measure on the open set $\mathcal {M}_+(\mathcal {I}\times \mathbb {T}^d)$. Let ${\varvec{\nu }}$ be the restriction of $\bar{{\varvec{\nu }}}$ to $\mathcal {I}\times \mathbb {T}^d$. Since ${\varvec{\mu }}^\varepsilon$ (resp. ${\varvec{\nu }}^\varepsilon$) converges vaguely to ${\varvec{\mu }}$ (resp. ${\varvec{\nu }}$), it follows that $({\varvec{\mu }}, {\varvec{\nu }})$ belongs to $\mathbb{C}\mathbb{E}^\mathcal {I}$.

In view of (6.3), we can apply the $\textrm{BV}$-compactness theorem (see, e.g., [34, Theorem B.5.10]) to obtain a further subsequence such that $\Vert \mu _t^\varepsilon - \mu _t\Vert _{\textrm{KR}(\mathbb {T}^d)} \rightarrow 0$ for almost every $t \in \mathcal {I}$, and the limiting curve ${\varvec{\mu }}$ belongs to $\textrm{BV}_{\textrm{KR}}(\mathcal {I}; \mathcal {M}_+(\mathbb {T}^d))$. Proposition A.5 yields $\mu _t^\varepsilon \rightarrow \mu _t$ weakly in $\mathcal {M}_+(\mathbb {T}^d)$ for almost every $t \in \mathcal {I}$.$\square$

### Uniform compactness under superlinear growth

In the last two sections, we shall work with the stronger growth condition from Assumption 5.5.

#### Remark 6.1

(Property of $f_\textrm{hom}$, superlinear case) Let us first observe that under Assumption 5.5, one has superlinear growth of $f_\textrm{hom}$:

$$\begin{aligned} f_\textrm{hom}(\rho , j) \ge \theta \Big ( \frac{|j|}{\rho +1} \Big )(\rho +1) - C (\rho + 1) , \quad \forall \rho \ge 0, \; j \in \mathbb {R}^d, \end{aligned}$$

where we recall $\theta :[0,\infty ) \rightarrow [0,\infty )$ is such that $\lim _{t \rightarrow \infty } \frac{\theta (t)}{t} = +\infty$.

In addition for all $j\ne 0$ we have

6.4 $$\begin{aligned} f_\textrm{hom}^\infty (0,j) = \lim _{t\rightarrow \infty } \frac{1}{t} f_\textrm{hom}(\rho _0, j_0 + tj) \ge \lim _{t\rightarrow \infty } \frac{\theta \left( \frac{|j_0 + t j|}{\rho _0+1} \right) (\rho _0+1)}{t} = \infty . \end{aligned}$$

In particular, if $\mathbb {A}_\textrm{hom}^\mathcal {I}({\varvec{\mu }}, {\varvec{\nu }})<\infty$, then ${\varvec{\nu }}\ll {\varvec{\mu }}+ \mathscr {L}^{d+1}$. Indeed, fix ${\varvec{\sigma }}\in \mathcal {M}_+(\mathcal {I}\times \mathbb {T}^d)$ as in (3.5) and suppose that $({\varvec{\mu }}+ \mathscr {L}^{d+1})(A) = 0$ for some $A \subset \mathcal {I}\times \mathbb {T}^d$. By positivity of the measures, this implies that ${\varvec{\mu }}(A) = \mathscr {L}^{d+1}(A) =0$, thus by construction

$$\begin{aligned} {\varvec{\mu }}^\perp (A) = 0 \quad \text { and }\quad {\varvec{\nu }}(A) = {\varvec{\nu }}^\perp (A). \end{aligned}$$

From the first condition and ${\varvec{\mu }}^\perp = \rho ^\perp {\varvec{\sigma }}$, we deduce that $\rho ^\perp (t,x) = 0$ for ${\varvec{\sigma }}$-a.e. $(t,x) \in A$. From the assumption of finite energy and (6.4), writing ${\varvec{\nu }}^\perp = j^\perp {\varvec{\sigma }}$, we infer that $j^\perp (t,x) = 0$ for ${\varvec{\sigma }}$-a.e. $(t,x) \in A$ as well. It follows that ${\varvec{\nu }}(A) = {\varvec{\nu }}^\perp (A) = 0$, which proves the claim.

We are ready to prove Theorem 5.9.

#### Proof of Theorem 5.4

(Proof of Theorem 5.9) Let $\{{\pmb {m}}^\varepsilon \}_\varepsilon$ be a sequence of measures such that

6.5 $$\begin{aligned} M := \sup _\varepsilon {\pmb {m}}^\varepsilon (\mathcal {I}\times \mathcal {X}_\varepsilon ) +1< \infty \quad \text { and }\quad A := \sup _\varepsilon \mathcal {A}_\varepsilon ^\mathcal {I}({\pmb {m}}^\varepsilon ) < \infty . \end{aligned}$$

Thanks to Remark 6.1, we have that ${\varvec{\nu }}\ll {\varvec{\mu }}+ \mathscr {L}^{d+1}$ for all solutions $({\varvec{\mu }},{\varvec{\nu }})\in \mathbb{C}\mathbb{E}^\mathcal {I}$ with $\mathbb {A}_{\textrm{hom}}^\mathcal {I}({\varvec{\mu }})<\infty$. Applying Lemma 3.13 we can write ${\varvec{\mu }}= \, \textrm{d}t\otimes \mu _t$ and because $\mathscr {L}^{d+1} = \, \textrm{d}t\otimes \mathscr {L}^d$, we also have disintegration ${\varvec{\nu }}= \, \textrm{d}t\otimes \nu _t$ with $\nu _t \ll \mu _t + \mathscr {L}^d$ for almost every $t\in \mathcal {I}$.

Moreover, it follows from the definition of $\mathbb{C}\mathbb{E}^\mathcal {I}$ that, for any test function $\varphi \in \mathcal {C}_c^1 (\mathcal {I}; \mathcal {C}^1(\mathbb {T}^d))$ we have

$$\begin{aligned} \langle {\varvec{\mu }}, \partial _t \varphi \rangle = -\langle {\varvec{\nu }}, \nabla \varphi \rangle = -\int _\mathcal {I}\Big \langle \frac{\, \textrm{d}\nu _t}{\, \textrm{d}( \mu _t + \mathscr {L}^{d})} (\mu _t + \mathscr {L}^{d} ), \nabla \varphi \Big \rangle \, \textrm{d}t. \end{aligned}$$

This shows that $\, \textrm{d}t \otimes \mu _t \in W_\textrm{KR}^{1,1}(\mathcal {I}; \mathcal {M}_+(\mathbb {T}^d))$, with weak derivative

$$\begin{aligned} \partial _t\mu _t = \nabla \cdot \Big ( \frac{\, \textrm{d}\nu _t}{\, \textrm{d}( \mu _t + \mathscr {L}^{d})} (\mu _t + \mathscr {L}^{d} ) \Big ) \in \textrm{KR}(\mathbb {T}^d) \quad \text {for a.e.} \; t \in \mathcal {I}. \end{aligned}$$

We are left with showing uniform convergence of $\iota _\varepsilon m^\varepsilon _t \rightarrow \mu _t$ in $\textrm{KR}(\mathbb {T}^d)$. We claim that the curves $\{ t \mapsto \iota _\varepsilon m_t^\varepsilon \}_\varepsilon$ are equicontinuous with respect to the Kantorovich–Rubinstein norm $\Vert \cdot \Vert _{\textrm{KR}(\mathbb {T}^d)}$.

To show the claimed equicontinuity, take $\varphi \in \mathcal {C}^1(\mathbb {T}^d)$ and $s, t \in \mathcal {I}$ with $s<t$. Since $(\iota _\varepsilon m_t^\varepsilon , \iota _\varepsilon J_t^\varepsilon ) \in \mathbb{C}\mathbb{E}^\mathcal {I}$ we obtain using Lemma 4.10,

6.6 $$\begin{aligned} \begin{aligned} \bigg | \int _{\mathbb {T}^d} \varphi \, \textrm{d}(\iota _\varepsilon m^\varepsilon _t) - \int _{\mathbb {T}^d} \varphi \, \textrm{d}(\iota _\varepsilon m^\varepsilon _s) \bigg |&= \bigg | \int _s^t \int _{\mathbb {T}^d} \nabla \varphi \cdot \, \textrm{d}(\iota _\varepsilon J^\varepsilon _r) \, \textrm{d}r \bigg | \\ {}&\le \Vert \nabla \varphi \Vert _{\mathcal {C}(\mathbb {T}^d)} \int _s^t |\iota _\varepsilon J_r^\varepsilon |(\mathbb {T}^d) \, \textrm{d}r \\ {}&\le \frac{R_0 \sqrt{d}}{2} \Vert \nabla \varphi \Vert _{\mathcal {C}(\mathbb {T}^d)} \int _s^t \sum _{(x,y)\in \mathcal {E}_\varepsilon } \varepsilon |J^\varepsilon _r(x,y)|\, \textrm{d}r, \end{aligned} \end{aligned}$$

To estimate the latter integral, we consider for $z\in \mathbb {Z}_\varepsilon ^d$ the quantities

$$\begin{aligned} \textsf{m}^\varepsilon _r(z) := \sum _{\begin{array}{c} x\in \mathcal {X}_\varepsilon \\ |x_\textsf{z}- z|_{\ell _\infty ^d} \le R \end{array} } m^\varepsilon _r(x) \quad \text { and }\quad \textsf{J}^\varepsilon _r(z) := \sum _{\begin{array}{c} (x,y)\in \mathcal {E}_\varepsilon \\ x_z = z \end{array} } |J^\varepsilon _r(x,y)|. \end{aligned}$$

We fix a “velocity threshold” $v_0 > 0$, and split $\mathbb {Z}_\varepsilon ^d$ into the low velocity region $\mathcal {Z}_-:= \{ z \in \mathbb {Z}_\varepsilon ^d \, \ \frac{\varepsilon |\textsf{J}^\varepsilon _r(z)|}{\textsf{m}^\varepsilon _r(z) + \varepsilon ^d} \le v_0 \}$ and its complement $\mathcal {Z}_+:= \mathbb {Z}_\varepsilon ^d \setminus \mathcal {Z}_-$. Then:

6.7 $$\begin{aligned} \sum _{z\in \mathcal {Z}_-} \varepsilon \textsf{J}^\varepsilon _r(z) \le v_0 \sum _{z \in \mathcal {Z}_-} \big ( \textsf{m}^\varepsilon _r(z) + \varepsilon ^d \big ) \le C_R \big ( m^\varepsilon _r(\mathcal {X}_\varepsilon ) + 1 \big ) v_0, \end{aligned}$$

where $C_R:= (2R + 1)^d$. For $z \in \mathcal {Z}_+$ we use the growth condition (5.4) to estimate

$$\begin{aligned} \begin{aligned} \varepsilon \textsf{J}^\varepsilon _r(z)&\le \big ( \textsf{m}^\varepsilon _r(z) + \varepsilon ^d \big ) \theta \Big ( \frac{\varepsilon \textsf{J}^\varepsilon _r(z)}{\textsf{m}^\varepsilon _r(z) + \varepsilon ^d} \Big ) \sup _{v> v_0} \frac{v}{\theta (v)} \\&\le \varepsilon ^d \bigg ( F\bigg (\frac{\tau _\varepsilon ^z m}{\varepsilon ^d}, \frac{\tau _\varepsilon ^zJ}{\varepsilon ^{d-1}} \bigg ) + C \Big (\frac{\textsf{m}^\varepsilon _r(z)}{\varepsilon ^d} + 1 \Big ) \bigg ) \sup _{v > v_0} \frac{v}{\theta (v)}. \end{aligned} \end{aligned}$$

Since (5.4) implies non-negativity of the term in brackets, we obtain

6.8 $$\begin{aligned} \begin{aligned} \sum _{z\in \mathcal {Z}_+} \varepsilon \textsf{J}^\varepsilon _r(z)&\le \sum _{z\in \mathbb {T}^d} \varepsilon ^d \bigg ( F\bigg (\frac{\tau _\varepsilon ^z m}{\varepsilon ^d}, \frac{\tau _\varepsilon ^z J}{\varepsilon ^{d-1}} \bigg ) + C \Big (\frac{\textsf{m}^\varepsilon _r(z)}{\varepsilon ^d} + 1 \Big ) \bigg ) \sup _{v> v_0} \frac{v}{\theta (v)} \\ {}&\le \mathcal {F}_\varepsilon (m^\varepsilon _r, J^\varepsilon _r) + C \big ( m^\varepsilon _r(\mathcal {X}_\varepsilon ) + 1 \big ) \sup _{v > v_0} \frac{v}{\theta (v)}. \end{aligned} \end{aligned}$$

Integrating in time, we combine (6.7) and (6.8) with (6.5) to obtain

6.9 $$\begin{aligned} \begin{aligned}&\int _s^t \sum _{(x,y)\in \mathcal {E}_\varepsilon } \varepsilon |J^\varepsilon _r(x,y)| \, \textrm{d}r = \int _s^t \sum _{z \in \mathbb {Z}_\varepsilon ^d} \varepsilon \textsf{J}^\varepsilon _r(z) \, \textrm{d}r \le g(t-s), \\ {}&\text {where} \quad g(r) := \inf _{v_0> 0} \bigg \{ r C_R M v_0 + \Big ( A + C ( M + |\mathcal {I}| ) \Big ) \sup _{v > v_0} \frac{v}{\theta (v)} \bigg \}. \end{aligned}\end{aligned}$$

Combining (6.6) and (6.9) we conclude that

$$\begin{aligned} \sup _{\varepsilon> 0} \Vert \iota _\varepsilon m^\varepsilon _t - \iota _\varepsilon m^\varepsilon _s\Vert _{\textrm{KR}(\mathbb {T}^d)}&\le \sup _{\varepsilon >0} \sup _{\Vert \varphi \Vert _{\mathcal {C}^1(\mathbb {T}^d)}\le 1} \bigg | \int _{\mathbb {T}^d} \varphi \, \textrm{d}(\iota _\varepsilon m^\varepsilon _t) - \int _{\mathbb {T}^d} \varphi \, \textrm{d}(\iota _\varepsilon m^\varepsilon _s) \bigg | \\ {}&\le \frac{R_0 \sqrt{d}}{2} g(t-s). \end{aligned}$$

To prove the claimed equicontinuity, it suffices to show that $g(r) \rightarrow 0$ as $r \rightarrow 0$. But this follows from the growth properties of $\theta$ by picking, e.g., $v_0:= r^{-1/2}$.

Of course the masses are uniformly bounded in $\varepsilon$ and *t*. The Arzela-Ascoli theorem implies that every subsequence has a subsequence converging uniformly in $\big (\mathcal {M}_+(\mathbb {T}^d), \Vert \cdot \Vert _\textrm{KR}\big )$.$\square$

### The boundary value problems under superlinear growth

The last part of this section is devoted to the proof of the convergence of the minimal actions, under the assumption of superlinear growth, i.e. Theorem 5.10. The proof is a straightforward consequence of the stronger compactness result Theorem 5.9 (and the general convergence result Theorem 5.1) proved in the previous section, which ensures the stability of the boundary conditions as well. We fix $\mathcal {I}= (a,b)$.

#### Proof of Theorem 5.10

We shall prove the upper and the lower bound.

*Liminf inequality*. Pick any $\iota _\varepsilon m_a^\varepsilon \rightarrow \mu ^a$, $\iota _\varepsilon m_b^\varepsilon \rightarrow \mu ^b$ weakly in $\mathcal {M}_+(\mathbb {T}^d)$, and let $({\pmb {m}}^\varepsilon , \pmb {J}^\varepsilon ) \in \mathcal{C}\mathcal{E}_\varepsilon ^\mathcal {I}$ with the same boundary data such that

$$\begin{aligned} \lim _{\varepsilon \rightarrow 0} \mathcal {A}_\varepsilon ^\mathcal {I}(\textbf{m}^\varepsilon , \textbf{J}^\varepsilon ) = \lim _{\varepsilon \rightarrow 0} \mathcal{M}\mathcal{A}_\varepsilon ^\mathcal {I}(m_a^\varepsilon , m_b^\varepsilon ) < \infty . \end{aligned}$$

By Theorem 5.9, there exists a (non-relabeled) subsequence of ${\pmb {m}}^\varepsilon$ such that $\Vert \iota _\varepsilon m_t^\varepsilon - \mu _t \Vert _\textrm{KR}\rightarrow 0$, uniformly for $t\in \overline{\mathcal {I}}$. In particular, $\mu _a = \mu ^a$, $\mu _b = \mu ^b$. We can then apply the lower bound of Theorem 5.1, and conclude

$$\begin{aligned} \mathbb{M}\mathbb{A}_{\textrm{hom}}^\mathcal {I}(\mu ^a,\mu ^b) \le \mathbb {A}_{\textrm{hom}}^\mathcal {I}({\varvec{\mu }}) \le \liminf _{\varepsilon \rightarrow \infty } \mathcal{M}\mathcal{A}_\varepsilon ^\mathcal {I}(m_a^\varepsilon , m_b^\varepsilon ). \end{aligned}$$

*Limsup inequality*. Fix $\mu ^a, \mu ^b \in \mathcal {M}_+(\mathbb {T}^d)$ such that $\mathbb{M}\mathbb{A}_{\textrm{hom}}^\mathcal {I}(\mu ^a,\mu ^b)<\infty$. By the definition of $\mathbb{M}\mathbb{A}_{\textrm{hom}}^\mathcal {I}$ and the lower semicontinuity of $\mathbb {A}_\textrm{hom}$ (Lemma 3.14), there exists ${\varvec{\mu }}\in \mathcal {M}_+(\mathcal {I}\times \mathbb {T}^d)$ with $\mathbb {A}_\textrm{hom}^\mathcal {I}(\varvec{\mu }) = \mathbb{M}\mathbb{A}_{\textrm{hom}}^\mathcal {I}(\mu ^a,\mu ^b)$ and $\mu _a = \mu ^a, \mu _b= \mu ^b$.

We can then apply Theorem 5.1 and find a recovery sequence $(\textbf{m}^\varepsilon , \textbf{J}^\varepsilon ) \in \mathcal{C}\mathcal{E}_\varepsilon ^\mathcal {I}$ such that $\iota _\varepsilon {\pmb {m}}^\varepsilon \rightarrow {\varvec{\mu }}$ weakly and

$$\begin{aligned} \limsup _{\varepsilon \rightarrow 0} \mathcal {A}_\varepsilon ^\mathcal {I}(\textbf{m}^\varepsilon , \textbf{J}^\varepsilon ) \le \mathbb {A}_\textrm{hom}^\mathcal {I}({\varvec{\mu }}) = \mathbb{M}\mathbb{A}_{\textrm{hom}}^\mathcal {I}(\mu ^a,\mu ^b). \end{aligned}$$

By the improved compactness result Theorem 5.9, $\iota _\varepsilon m^\varepsilon _t \rightarrow \mu _t$ in $\textrm{KR}(\mathbb {T}^d)$ for every $t\in \overline{\mathcal {I}}$, in particular for $t=a,b$. This allows us to conclude

$$\begin{aligned} \limsup _{\varepsilon \rightarrow 0} \mathcal{M}\mathcal{A}_\varepsilon ^\mathcal {I}(m_a^\varepsilon , m_b^\varepsilon ) \le \mathbb{M}\mathbb{A}_{\textrm{hom}}^\mathcal {I}(\mu ^a,\mu ^b) , \quad \text { and }\quad \iota _\varepsilon m_i^\varepsilon \rightarrow \mu ^i \, \text {weakly} \end{aligned}$$

for $i=a,b$, which is the sought recovery sequence for $\mathbb{M}\mathbb{A}_{\textrm{hom}}^\mathcal {I}(\mu ^a,\mu ^b)$.

#### Remark 6.2

It is instructive to see that under the simple linear growth condition 2.3, the above written proof cannot be carried out. Indeed, by the lack of compactness in $W^{1,1}(\mathcal {I}; \mathcal {M}_+(\mathbb {T}^d))$ (but rather only in $\textrm{BV}$ by Theorem 5.4), we are not able to ensure stability at the level of the initial data, i.e. in general, $\mu _a \ne \mu ^a$ (and similarly for $t =b$).

## Proof of the lower bound

In this section we present the proof of the lower bound in our main result, Theorem 5.1. The proof relies on two key ingredients. The first one is a partial regularisation result for discrete measures of bounded action, which is stated in Proposition 7.1 and proved in Sect. 7.1 below. The second ingredient is a lower bound of the energy under partial regularity conditions on the involved measures (Proposition 7.4). The proof of the lower bound in Theorem 5.1, which combines both ingredients, is given right before Sect. 7.1.

First we state the regularisation result. Recall the Kantorovich–Rubinstein norm $\Vert \cdot \Vert _{\textrm{KR}}$ (see “Appendix 1”).

### Proposition 7.1

(Discrete Regularisation) Fix $\varepsilon < \frac{1}{2R_0}$ and let $({\pmb {m}}, \pmb {J}) \in \mathcal{C}\mathcal{E}_\varepsilon ^\mathcal {I}$ be a solution to the discrete continuity equation satisfying

$$\begin{aligned} M := m_0(\mathcal {X}_\varepsilon )< \infty \quad \text { and }\quad A := \mathcal {A}_\varepsilon ^\mathcal {I}({\pmb {m}}, \pmb {J}) < \infty . \end{aligned}$$

Then, for any $\eta > 0$ there exists an interval $\mathcal {I}^\eta \subset \mathcal {I}:=(0,T)$ with $|\mathcal {I}{\setminus } \mathcal {I}^\eta | \le \eta$ and a solution $(\widetilde{{\pmb {m}}}, \widetilde{\pmb {J}}) \in \mathcal{C}\mathcal{E}_\varepsilon ^{\mathcal {I}^\eta }$ such that:

(i) the following approximation properties hold:
  7.1a $$\begin{aligned}&\text {(measure approximation)}&\Vert \iota _\varepsilon (\widetilde{{\pmb {m}}} - {\pmb {m}}) \Vert _{\textrm{KR}(\overline{\mathcal {I}^\eta } \times \mathbb {T}^d)}&\le \eta , \end{aligned}$$
  7.1b $$\begin{aligned}&\text {(action approximation)}&\mathcal {A}_\varepsilon ^{\mathcal {I}^\eta }(\widetilde{{\pmb {m}}}, \widetilde{\pmb {J}}) \le \mathcal {A}_\varepsilon ^\mathcal {I}({\pmb {m}}, \pmb {J})&+ \eta . \end{aligned}$$
(ii) the following regularity properties hold, uniformly for any $t\in \mathcal {I}^\eta$ and any $z \in \mathbb {T}_\varepsilon ^d$:
  7.2a $$\begin{aligned}&\text {(boundedness)}&\big \Vert \widetilde{m}_t \big \Vert _{\ell ^\infty (\mathcal {X}_\varepsilon )} + \varepsilon \big \Vert \widetilde{J}_t \big \Vert _{\ell ^\infty (\mathcal {E}_\varepsilon )}&\le C_B \varepsilon ^{d}, \end{aligned}$$
  7.2b $$\begin{aligned}&\text {(time-reg.)}&\big \Vert {{\,\mathrm{\text {\textsf{div}}}\,}}\widetilde{J}_t \big \Vert _{\ell ^\infty (\mathcal {X}_\varepsilon )}&\le C_T \varepsilon ^{d}, \end{aligned}$$
  7.2c $$\begin{aligned}&\text {(space-reg.)}&\big \Vert \sigma _\varepsilon ^z \widetilde{m}_t - \widetilde{m}_t \big \Vert _{\ell ^\infty (\mathcal {X}_\varepsilon )} + \varepsilon \big \Vert \sigma _\varepsilon ^z \widetilde{J}_t - \widetilde{J}_t \big \Vert _{\ell ^\infty (\mathcal {E}_\varepsilon )}&\le C_S |z| \varepsilon ^{d+1}, \end{aligned}$$
  7.2d $$\begin{aligned}&\text {(domain reg.)}&\bigg (\frac{\tau _\varepsilon ^z \widetilde{m}_t}{\varepsilon ^d} , \frac{\tau _\varepsilon ^z \widetilde{J}_t}{\varepsilon ^{d-1}} \bigg )&\in K. \end{aligned}$$
  The constants $C_B, C_T, C_S < \infty$ and the compact set $K \subseteq {{\,\mathrm{\textsf{D}}\,}}(F)^\circ$ depend on $\eta$, *M* and *A*, but not on $\varepsilon$.

### Remark 7.2

The $\ell ^\infty$-bounds in (7.2a) are explicitly stated for the sake of clarity, although they are implied by the compactness of the set *K* in (7.2d).

Since $(\widetilde{{\pmb {m}}}, \widetilde{\pmb {J}})\in \mathcal{C}\mathcal{E}_\varepsilon ^{\mathcal {I}^\eta }$, inequality (7.2b) in effect bounds $\big \Vert \partial _t \widetilde{m}_t \big \Vert _{\ell ^\infty (\mathcal {X}_\varepsilon )} \le C_T \varepsilon ^{d}$.

In the next result, we start by showing how to construct $\mathbb {Z}^d$-periodic solutions to the static continuity equation by superposition of unit fluxes. Additionally, we can build these solutions with vanishing effective flux and ensure good $\ell ^\infty$-bounds.

### Lemma 7.3

[Periodic solutions to the divergence equation] Let $g: \mathcal {X}\rightarrow \mathbb {R}$ be a $\mathbb {Z}^d$-periodic function with $\sum _{x \in \mathcal {X}^Q} g(x) = 0$. There exists a $\mathbb {Z}^d$-periodic discrete vector field $J: \mathcal {E}\rightarrow \mathbb {R}$ satisfying

$$\begin{aligned} {{\,\mathrm{\text {\textsf{div}}}\,}}J = g, \quad {{\,\mathrm{\textsf{Eff}}\,}}(J) = 0, \quad \text { and }\quad \Vert J \Vert _{\ell _\infty (\mathcal {E}^Q)} \le \tfrac{1}{2} \Vert g \Vert _{\ell _1(\mathcal {X}^Q)}. \end{aligned}$$

### Proof

For any $v, w \in V$, fix a simple path $P^{vw}$ in $(\mathcal {X}, \mathcal {E})$ connecting (0, *v*) and (0, *w*). Let $\widetilde{J}_{vw}:= \widetilde{J}_{P^{vw}}$ be the associated periodic unit flux defined in (4.3). Since $\sum _{v \in V} g(0,v) = 0$, we can pick a coupling $\Gamma$ between the negative part and the positive part of *g*. More precisely, we may pick a function $\Gamma : V\times V\rightarrow \mathbb {R}_+$ with $\sum _{v,w \in V} \Gamma (v,w) = \frac{1}{2}\Vert g\Vert _{\ell _1(\mathcal {X}^Q)}$ such that

$$\begin{aligned} \sum _{w \in V} \Gamma _{vw} = g_-(0,v) \quad \text {for } v \in V, \quad \text { and }\quad \sum _{v \in V} \Gamma _{vw} = g_+(0,w) \quad \text {for } w \in V. \end{aligned}$$

We then define

$$\begin{aligned} J := \sum _{v,w \in V} \Gamma _{vw} \widetilde{J}_{vw}. \end{aligned}$$

It is straightforward to verify using Lemma 4.5 that *J* has the three desired properties.

The following result states the desired relation between the functionals $\mathcal {F}_\varepsilon$ and $\mathbb {F}_{\textrm{hom}}$ under suitable regularity conditions for the measures involved. These regularity conditions are consistent with the regularity properties obtained in Proposition 7.1.

### Proposition 7.4

(Energy lower bound for regular measures) Let $C_B, C_T, C_S < \infty$ and let $K \subseteq {{\,\mathrm{\textsf{D}}\,}}(F)^\circ$ be a compact set. There exists a threshold $\varepsilon _0 > 0$ and a constant $C < \infty$ such that the following implication holds for any $\varepsilon < \varepsilon _0$: if $m \in \mathbb {R}_+^{\mathcal {X}_\varepsilon }$ and $J \in \mathbb {R}_a^{\mathcal {E}_\varepsilon }$ satisfy the regularity properties (7.2a)–(7.2d), then we have the energy bound

$$\begin{aligned} \mathbb {F}_\textrm{hom}(\iota _\varepsilon m, \iota _\varepsilon J) \le \mathcal {F}_\varepsilon (m, J) + C \varepsilon . \end{aligned}$$

### Proof

Recall from (4.11) that $\iota _\varepsilon m = \rho \mathscr {L}^d$ and $\iota _\varepsilon J = j \mathscr {L}^d$, where, for $\bar{z} \in \mathbb {Z}_\varepsilon ^d$ and $u \in Q_\varepsilon ^{\bar{z}}$,

$$\begin{aligned} \rho (u) := \varepsilon ^{-d} \sum _{\begin{array}{c} x \in \mathcal {X}_\varepsilon \\ x_\textsf{z}= \bar{z} \end{array}} m(x) \quad \text { and }\quad j(u) := \frac{1}{2\varepsilon ^{d-1}} \sum _{\begin{array}{c} (x,y) \in \mathcal {E}_\varepsilon \\ x_\textsf{z}= \bar{z} \end{array}} J_u(x,y) \big ( y_\textsf{z}- x_\textsf{z}\big ), \end{aligned}$$

where $J_u(x, y)$ is a convex combination of $\big \{ J\big ( T_\varepsilon ^z x, T_\varepsilon ^z y \big ) \big \}_{z \in \mathbb {Z}_\varepsilon ^d}$, i.e.,

$$\begin{aligned} J_u(x, y)&= \sum _{z \in \mathbb {Z}_\varepsilon ^d} \lambda _u^{\varepsilon ,z}(x,y) J\big ( T_\varepsilon ^z x, T_\varepsilon ^z y \big ), \end{aligned}$$

where $\lambda _u^{\varepsilon , {\bar{z}}}(x,y) \ge 0$, $\sum _{z \in \mathbb {Z}_\varepsilon ^d} \lambda _u^{\varepsilon ,z}(x,y) = 1$, and $\lambda _u^{\varepsilon , z}(x,y) = 0$ whenever $|z| > R_0$.

*Step 1. Construction of a representative.* Fix $\bar{z} \in \mathbb {Z}_\varepsilon ^d$ and $u \in Q_\varepsilon ^{\bar{z}}$. Our first goal is to construct a representative

$$\begin{aligned} \bigg ( \frac{\widehat{m}_u}{\varepsilon ^d}, \frac{\widehat{J}_u}{\varepsilon ^{d-1}} \bigg ) \in {{\,\mathrm{{\textsf{Rep}}}\,}}\big ( \rho (u), j(u) \big ). \end{aligned}$$

For this purpose we define candidates $\widehat{m}_u \in \mathbb {R}_+^{\mathcal {X}}$ and $\widetilde{J}_u \in \mathbb {R}_a^{\mathcal {E}}$ as follows. We take the values of *m* and $J_u$ in the $\varepsilon$-cube at $\bar{z}$, and insert these values at every cube in $(\mathcal {X}, \mathcal {E})$, so that the result is $\mathbb {Z}^d$-periodic. In formulae:

$$\begin{aligned} \widehat{m}_u(z,v)&:= m(\varepsilon \bar{z}, v){} & {} \text {for } (z, v) \in \mathcal {X}\\ \widetilde{J}_u\Big ( (z,v), (z',v') \Big )&:= J_u\Big ( (\varepsilon \bar{z},v), (\varepsilon (\bar{z}+ z' - z),v') \Big ){} & {} \text {for } \Big ( (z,v), (z',v') \Big ) \in \mathcal {E}. \end{aligned}$$

see Fig. 5.

**Fig. 5:**
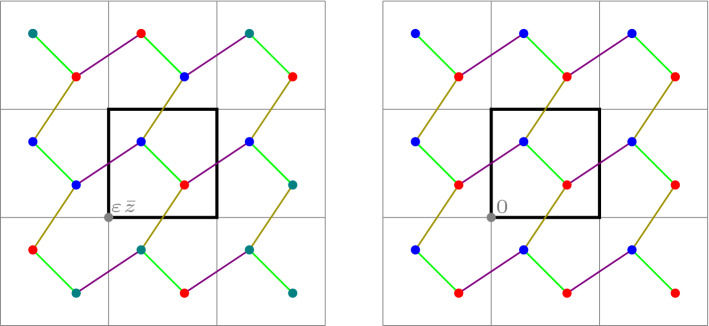
On the left, using different colors for different values, the measures *m* and $J_u$. On the right, the corresponding $\widehat{m}_u$ and $\widetilde{J}_u$, for $u \in Q_\varepsilon ^{\bar{z}}$ (color figure online)

We emphasise that the right-hand side does not depend on *z*, hence $m_u$ and $\widetilde{J}_u$ are $\mathbb {Z}^d$-periodic. Our construction also ensures that

$$\begin{aligned} \varepsilon ^{-d} \sum _{x \in \mathcal {X}^Q} \widehat{m}_u(x) = \rho (u), \end{aligned}$$

hence $\varepsilon ^{-d} \widehat{m}_u \in {{\,\mathrm{{\textsf{Rep}}}\,}}\big ( \rho (u) \big )$. However, the vector field $\varepsilon ^{-(d-1)}\widetilde{J}_u$ does (in general) not belong to ${{\,\mathrm{{\textsf{Rep}}}\,}}\big (j(u)\big )$: indeed, while $\widetilde{J}_u$ has the desired effective flux (i.e., ${{\,\mathrm{\textsf{Eff}}\,}}\big (\varepsilon ^{-(d-1)}\widetilde{J}_u\big ) = j(u)$), $\widetilde{J}_u$ is not (in general) divergence-free.

To remedy this issue, we introduce a *corrector field* $\bar{J}_u$, i.e., an anti-symmetric and $\mathbb {Z}^d$-periodic function $\bar{J}_u: \mathcal {E}\rightarrow \mathbb {R}$ satisfying

7.3 $$\begin{aligned} {{\,\mathrm{\text {\textsf{div}}}\,}}\bar{J}_u = -{{\,\mathrm{\text {\textsf{div}}}\,}}\widetilde{J}_u, \quad {{\,\mathrm{\textsf{Eff}}\,}}(\bar{J}_u ) = 0, \quad \text { and }\quad \big \Vert \bar{J}_u \big \Vert _{\ell ^\infty (\mathcal {E}^Q)} \le \tfrac{1}{2} \big \Vert {{\,\mathrm{\text {\textsf{div}}}\,}}\widetilde{J}_u \big \Vert _{\ell ^1(\mathcal {X}^Q)}. \end{aligned}$$

The existence of such a vector field is guaranteed by Lemma 7.3. It immediately follows that $\widehat{J}_u:= \widetilde{J}_u + \bar{J}_u$ satisfies ${{\,\mathrm{\text {\textsf{div}}}\,}}\widehat{J}_u = 0$ and ${{\,\mathrm{\textsf{Eff}}\,}}\big (\varepsilon ^{-(d-1)}\widehat{J}_u\big ) = j(u)$, thus

$$\begin{aligned} \frac{\widehat{J}_u}{\varepsilon ^{d-1}} := \frac{\widetilde{J}_u + \bar{J}_u}{{\varepsilon ^{d-1}}} \in {{\,\mathrm{{\textsf{Rep}}}\,}}\big (j_u \big ). \end{aligned}$$

*Step 2. Density comparison.* We will now use the regularity assumptions (7.2a)-(7.2d) to show that the representative $(\widehat{m}_u, \widehat{J}_u)$ is not too different from the shifted density $(\tau _{\bar{z}} m, \tau _{\bar{z}} J)$. Indeed, for $x = (z, v) \in \mathcal {X}$ with $|z| \le R_1$ we obtain using (7.2c),

7.4 $$\begin{aligned} | \tau _\varepsilon ^{\bar{z}} m(x) - \widehat{m}_u(x) | = \big | m\big (\varepsilon (\bar{z} + z), v \big ) - m\big (\varepsilon \bar{z}, v \big ) \big | \le C_S \varepsilon ^{d+1} |z|. \end{aligned}$$

Let us now turn to the momentum field. For $(x, y) = \big ( (z,v), (z',v') \big ) \in \mathcal {E}$ with $|z|, |z'| \le R_1$, we have, using (7.2c),

$$\begin{aligned}&\big | \tau _\varepsilon ^{\bar{z}} J(x,y) - \widetilde{J}_u(x,y) \big | \\ {}&= \Big | J\Big ( \big (\varepsilon (\bar{z} + z),v\big ), \big (\varepsilon (\bar{z} + z'),v'\big ) \Big ) - J_u\Big ( \big (\varepsilon \bar{z},v\big ), \big (\varepsilon (\bar{z} + z' - z),v'\big ) \Big ) \Big | \\ {}&= \bigg | \sum _{\widetilde{z} \in \mathbb {Z}_\varepsilon ^d} \lambda _u^{\varepsilon ,\widetilde{z}}(x,y) \bigg \{ J\Big ( \big (\varepsilon (\bar{z} + z) , v \big ), \big (\varepsilon (\bar{z} + z'), v' \big ) \Big ) \\ {}&\qquad \qquad \qquad \qquad - J\Big ( \big ( \varepsilon (\bar{z} + \widetilde{z} ), v \big ), \big ( \varepsilon (\bar{z} + \widetilde{z} + z' - z), v' \big ) \Big ) \bigg \} \bigg | \\ {}&\le C_S \varepsilon ^{d} |z - \widetilde{z}| \le R_1 C_S \varepsilon ^{d} . \end{aligned}$$

Moreover, using (7.3), (7.2c), and (7.2b), we obtain

$$\begin{aligned} |\bar{J}_u(x,y) |&\le \tfrac{1}{2} \Vert {{\,\mathrm{\text {\textsf{div}}}\,}}\widetilde{J}_u \Vert _{\ell ^1(\mathcal {X}^Q)} \le C_T \Big ( \Vert {{\,\mathrm{\text {\textsf{div}}}\,}}J \Vert _{\ell ^\infty (\mathcal {E}_\varepsilon )} + \varepsilon ^d \Big ) \le C \varepsilon ^d, \end{aligned}$$

for some $C < \infty$ not depending on $\varepsilon$. Combining these bounds we obtain

7.5 $$\begin{aligned} | \tau _\varepsilon ^{\bar{z}} J(x,y) - \widehat{J}_u(x,y) |&\le | \tau _\varepsilon ^{\bar{z}} J(x,y) - \widetilde{J}_u(x,y) | + |\bar{J}_u(x,y) | \le C \varepsilon ^d. \end{aligned}$$

*Step 3. Energy comparison.* Since $\displaystyle \bigg (\frac{\tau _\varepsilon ^{\bar{z}} m}{\varepsilon ^d}, \frac{\tau _\varepsilon ^{\bar{z}} J}{\varepsilon ^{d-1}} \bigg ) \in K$ by assumption, it follows from (7.4) and (7.5) that $\displaystyle \bigg (\frac{\widehat{m}_u}{\varepsilon ^d}, \frac{\widehat{J}_u}{\varepsilon ^{d-1}} \bigg ) \in K'$ for $\varepsilon > 0$ sufficiently small. Here *K* is a compact set, possibly slightly larger than *K*, contained in ${{\,\mathrm{\textsf{D}}\,}}(\mathcal {F})^\circ$.

Since *F* is convex, it is Lipschitz continuous on compact subsets in the interior of its domain. In particular, it is Lipschitz continuous on $K'$. Therefore, there exists a constant $C_L < \infty$ depending on $\mathcal {F}$ and $K'$ such that

$$\begin{aligned} \mathcal {F}\bigg (\frac{\tau _\varepsilon ^{\bar{z}} m}{\varepsilon ^d} , \frac{\tau _\varepsilon ^{\bar{z}} J}{\varepsilon ^{d-1}} \bigg )&\ge \mathcal {F}\bigg (\frac{\widehat{m}_u}{\varepsilon ^d} , \frac{\widehat{J}_u}{\varepsilon ^{d-1}} \bigg ) - C_L \bigg ( \Big \Vert \frac{\tau _\varepsilon ^{\bar{z}} m - \widehat{m}_u}{\varepsilon ^d} \Big \Vert _{\ell ^\infty _{R_1}(\mathcal {X})} + \Big \Vert \frac{\tau _\varepsilon ^{\bar{z}} J - \widehat{J}_u }{\varepsilon ^{d-1}}\Big \Vert _{\ell ^\infty _{R_1}(\mathcal {E})} \bigg ) \\ {}&\ge \mathcal {F}\bigg (\frac{\widehat{m}_u}{\varepsilon ^d} , \frac{\widehat{J}_u}{\varepsilon ^{d-1}} \bigg ) - C \varepsilon \\ {}&\ge f_\textrm{hom}\big ( \rho (u), j(u) \big ) - C \varepsilon , \end{aligned}$$

with $C < \infty$ depending on $C_L$, $C_S$, $C_T$, and $R_1$, but not on $\varepsilon$. Here, the subscript $R_1$ in $\ell ^\infty _{R_1}(\mathcal {E})$ and $\ell ^\infty _{R_1}(\mathcal {X})$ indicates that only elements with $|x_\textsf{z}| \le R_1$ are considered.

Integration over $Q_\varepsilon ^{\bar{z}}$ followed by summation over $\bar{z} \in \mathbb {Z}_\varepsilon ^d$ yields

$$\begin{aligned} \mathcal {F}_\varepsilon (m, J)&= \varepsilon ^d \sum _{\bar{z} \in \mathbb {Z}_\varepsilon ^d} \mathcal {F}\bigg (\frac{\tau _\varepsilon ^{\bar{z}} m}{\varepsilon ^d} , \frac{\tau _\varepsilon ^{\bar{z}} J}{\varepsilon ^{d-1}} \bigg ) \ge \sum _{\bar{z} \in \mathbb {Z}_\varepsilon ^d} \int _{Q_\varepsilon ^{\bar{z}}} \Big ( f_\textrm{hom}\big ( \rho (u), j(u) \big ) - C \varepsilon \Big ) \, \textrm{d}u \\ {}&= \int _{\mathbb {T}^d} f_\textrm{hom}\big ( \rho (u), j(u) \big ) \, \textrm{d}u - C \varepsilon = \mathbb {F}_\textrm{hom}(\iota _\varepsilon m, \iota _\varepsilon J) - C \varepsilon , \end{aligned}$$

which is the desired result.

We are now ready to give the proof of the lower bound in our main result, Theorem 5.1. We use the notation $A \lesssim B$ to denote the inequality $A \le CB$ for some constant $C < \infty$ that only depends on the geometry of the graph $(\mathcal {X}, \mathcal {E})$, on the function *F* (see Assumption 2.3), and on the length of the time interval $\mathcal {I}$.

### Proof of Theorem 5.1 (lower bound)

Let ${\varvec{\mu }}\in \mathcal {M}_+\big (\mathcal {I}\times \mathbb {T}^d\big )$ and let $(m_t^\varepsilon )_{t \in \mathcal {I}} \subseteq \mathbb {R}_+^{\mathcal {X}_\varepsilon }$ be such that the induced measures ${\pmb {m}}^\varepsilon \in \mathcal {M}_+\big (\mathcal {I}\times \mathcal {X}_\varepsilon \big )$ defined by $\textrm{d}{\pmb {m}}^\varepsilon (t,x) = \textrm{d}m^\varepsilon _t(x) \, \textrm{d}t$ satisfy $\iota _\varepsilon {\pmb {m}}^\varepsilon \rightarrow {\varvec{\mu }}$ vaguely in $\mathcal {M}_+(\mathcal {I}\times \mathbb {T}^d)$ as $\varepsilon \rightarrow 0$. Observe that

$$\begin{aligned} M := \sup _{\varepsilon > 0} {\pmb {m}}^\varepsilon \big ( \mathcal {I}\times \mathcal {X}_\varepsilon \big ) < \infty . \end{aligned}$$

Without loss of generality, we may assume that

$$\begin{aligned} A := \sup _{\varepsilon > 0} \mathcal {A}_\varepsilon ({\pmb {m}}^\varepsilon ) < \infty . \end{aligned}$$

*Step 1 (Regularisation)*: Fix $\eta > 0$. Let $(J_t^\varepsilon )_{t \in \mathcal {I}} \subseteq \mathbb {R}_a^{\mathcal {E}_\varepsilon }$ be an approximately optimal discrete vector field, i.e.,

7.6 $$\begin{aligned} ({\pmb {m}}^\varepsilon , \pmb {J}^\varepsilon ) \in \mathcal{C}\mathcal{E}_\varepsilon ^\mathcal {I}\quad \text { and }\quad \mathcal {A}_\varepsilon ({\pmb {m}}^\varepsilon , \pmb {J}^\varepsilon ) \le \mathcal {A}_\varepsilon ({\pmb {m}}^\varepsilon ) + \eta . \end{aligned}$$

Using Proposition 7.1 we take an interval $\mathcal {I}^\eta \subset \mathcal {I}:=(0,T)$, $|\mathcal {I}{\setminus } \mathcal {I}^\eta |\le \eta$ and an approximating pair $(\widetilde{{\pmb {m}}{}}^\varepsilon , \widetilde{\pmb {J}{}}^\varepsilon ) \in \mathcal{C}\mathcal{E}_\varepsilon ^{I_\eta }$ satisfying

7.7 $$\begin{aligned} \Vert \iota _\varepsilon (\widetilde{{\pmb {m}}{}}^\varepsilon - {\pmb {m}}^\varepsilon ) \Vert _{\textrm{KR}(\overline{\mathcal {I}^\eta } \times \mathbb {T}^d)} \le \eta \quad \text { and }\quad \mathcal {A}_\varepsilon ^{\mathcal {I}^\eta }(\widetilde{{\pmb {m}}{}}^\varepsilon , \widetilde{\pmb {J}{}}^\varepsilon ) \le \mathcal {A}_\varepsilon ({\pmb {m}}^\varepsilon , \pmb {J}^\varepsilon ) + \eta , \end{aligned}$$

together with the regularity properties (7.2) for some constants $C_B, C_T, C_S < \infty$ and a compact set $K \subseteq {{\,\mathrm{\textsf{D}}\,}}(F)^\circ$ depending on $\eta$, but not on $\varepsilon$. By virtue of these regularity properties, we may apply Proposition 7.4 to $(\widetilde{{\pmb {m}}{}}^\varepsilon , \widetilde{\pmb {J}{}}^\varepsilon )$. This yields

7.8 $$\begin{aligned} \mathbb {A}_\textrm{hom}^{\mathcal {I}^\eta }(\iota _\varepsilon \widetilde{{\pmb {m}}{}}^\varepsilon , \iota _\varepsilon \widetilde{\pmb {J}{}}^\varepsilon ) = \int _{\mathcal {I}^\eta } \mathbb {F}_\textrm{hom}(\iota _\varepsilon \widetilde{m}_t^\varepsilon , \iota _\varepsilon \widetilde{J}_t^\varepsilon ) \, \textrm{d}t \le \int _{\mathcal {I}^\eta } \mathcal {F}_\varepsilon (\widetilde{m}_t^\varepsilon , \widetilde{J}_t^\varepsilon ) \, \textrm{d}t + C \varepsilon , \end{aligned}$$

with $C < \infty$ depending on $\eta$, but not on $\varepsilon$.

*Step 2 (Limit passage* $\varepsilon \rightarrow 0$): It follows by definition of the Kantorovich–Rubinstein norm that

$$\begin{aligned} \sup _\varepsilon \iota _\varepsilon \widetilde{\pmb {m}}^\varepsilon \big ( \overline{\mathcal {I}^\eta } \times \mathbb {T}^d \big )&\le \sup _\varepsilon \bigg ( \iota _\varepsilon {\pmb {m}}^\varepsilon \big ( \mathcal {I}\times \mathbb {T}^d \big ) + \Vert \iota _\varepsilon (\widetilde{{\pmb {m}}{}}^\varepsilon - {\pmb {m}}^\varepsilon ) \Vert _{\textrm{KR}(\overline{\mathcal {I}^\eta } \times \mathbb {T}^d)} \bigg ) \\&\le M + \eta . \end{aligned}$$

It follows from the growth condition (2.1) and (7.7) that

7.9 $$\begin{aligned} \begin{aligned} \sup _\varepsilon \big |\iota _\varepsilon \widetilde{\pmb {J}{}}^\varepsilon \big | \big ( \overline{\mathcal {I}^\eta } \times \mathbb {T}^d \big )&\lesssim \sup _\varepsilon \int _{\mathcal {I}^\eta } \varepsilon \Vert \widetilde{J}_t^\varepsilon \Vert _{\ell ^1(\mathcal {E}_\varepsilon )} \, \textrm{d}t \\ {}&\lesssim \sup _\varepsilon \int _{\mathcal {I}^\eta } \bigg ( 1 + \Vert \widetilde{m}_t^\varepsilon \Vert _{\ell ^1(\mathcal {X}_\varepsilon )} + \mathcal {F}_\varepsilon (\widetilde{m}_t^\varepsilon , \widetilde{J}_t^\varepsilon ) \bigg ) \, \textrm{d}t \\ {}&\le \sup _\varepsilon \bigg ( T + \iota _\varepsilon \widetilde{\pmb {m}}^\varepsilon \big ( \mathcal {I}^\eta \times \mathbb {T}^d \big ) + \mathcal {A}_\varepsilon ^{\mathcal {I}^\eta }(\widetilde{{\pmb {m}}{}}^\varepsilon , \widetilde{\pmb {J}{}}^\varepsilon ) \bigg ) \\ {}&\le T + (M + \eta ) + (A + 2 \eta ). \end{aligned} \end{aligned}$$

Therefore, there exist measures ${\varvec{\mu }}_\eta \in \mathcal {M}_+\big (\overline{\mathcal {I}^\eta } \times \mathbb {T}^d\big )$ and ${\varvec{\nu }}_\eta \in \mathcal {M}^d\big (\overline{\mathcal {I}^\eta } \times \mathbb {T}^d\big )$ and convergent subsequences satisfying

7.10 $$\begin{aligned} \iota _\varepsilon \widetilde{{\pmb {m}}{}}^\varepsilon \rightarrow {\varvec{\mu }}_\eta \ \text {and} \ \iota _\varepsilon \widetilde{\pmb {J}{}}^\varepsilon \rightarrow {\varvec{\nu }}_\eta \ \text {weakly in } \mathcal {M}_+(\overline{\mathcal {I}^\eta } \times \mathbb {T}^d) \ \text {and} \ \mathcal {M}^d(\overline{\mathcal {I}^\eta } \times \mathbb {T}^d) \ \text {as } \varepsilon \rightarrow 0. \end{aligned}$$

The vague lower semicontinuity of the limiting functional (see Lemma 3.14), combined with (7.6), (7.7), and (7.8) thus yields

7.11 $$\begin{aligned} \mathbb {A}_\textrm{hom}^{\mathcal {I}^\eta } ({\varvec{\mu }}_\eta , {\varvec{\nu }}_\eta )&\le \liminf _{\varepsilon \rightarrow 0} \mathbb {A}_\textrm{hom}^{\mathcal {I}^\eta }(\iota _\varepsilon \widetilde{{\pmb {m}}{}}^\varepsilon , \iota _\varepsilon \widetilde{\pmb {J}{}}^\varepsilon ) \le \liminf _{\varepsilon \rightarrow 0} \mathcal {A}_\varepsilon ({\pmb {m}}^\varepsilon ) + 2\eta . \end{aligned}$$

*Step 3* *(Limit passage* $\eta \rightarrow 0$*)*: Let $\varphi \in \textrm{Lip}_1 \big ( \overline{\mathcal {I}^\eta } \times \mathbb {T}^d\big )$, $\Vert \varphi \Vert _\infty \le 1$. For brevity, write $\langle {\varphi ,{\varvec{\mu }}}\rangle = \int _{\mathcal {I}^\eta \times \mathbb {T}^d} \varphi \, \textrm{d}{\varvec{\mu }}$. Since from (7.10) $\iota _\varepsilon {\pmb {m}}^\varepsilon \rightarrow {\varvec{\mu }}$ and $\iota _\varepsilon \widetilde{{\pmb {m}}{}}^\varepsilon \rightarrow {\varvec{\mu }}_\eta$ weakly, and $\Vert \iota _\varepsilon (\widetilde{{\pmb {m}}{}}^\varepsilon - {\pmb {m}}^\varepsilon ) \Vert _{\textrm{KR}(\overline{\mathcal {I}^\eta } \times \mathbb {T}^d)} \le \eta$ we obtain

$$\begin{aligned} \langle { \varphi , {\varvec{\mu }}_\eta - {\varvec{\mu }}}\rangle&\le \limsup _{\varepsilon \rightarrow 0} \Big ( \big |\big \langle { \varphi , {\varvec{\mu }}_\eta - \iota _\varepsilon \widetilde{{\pmb {m}}{}}^\varepsilon }\big \rangle \big | + \big |\big \langle { \varphi , \iota _\varepsilon (\widetilde{{\pmb {m}}{}}^\varepsilon -{\pmb {m}}^\varepsilon ) }\big \rangle \big | + \big |\big \langle { \varphi , \iota _\varepsilon {\pmb {m}}^\varepsilon - {\varvec{\mu }}}\big \rangle \big | \Big ) \\ {}&\le 0 + \eta + 0. \end{aligned}$$

It follows that $\Vert {\varvec{\mu }}_\eta - {\varvec{\mu }}\Vert _{\textrm{KR}(\overline{\mathcal {I}^\eta } \times \mathbb {T}^d)} \le 2\eta$, which together with $|\mathcal {I}{\setminus } \mathcal {I}^\eta |\le \eta$ implies ${\varvec{\mu }}_\eta \rightarrow {\varvec{\mu }}\in \mathcal {M}_+(\mathcal {I}\times \mathbb {T}^d)$ vaguely as $\eta \rightarrow 0$.

Furthermore, (7.9) implies that $\sup _{\eta }\big |{\varvec{\nu }}^\eta \big | \big ( \mathcal {I}^\eta \times \mathbb {T}^d \big ) < \infty$. Therefore, we may extract a subsequence so that ${\varvec{\nu }}_\eta \rightarrow {\varvec{\nu }}$ vaguely in $\mathcal {M}^d(\mathcal {I}\times \mathbb {T}^d)$ as $\eta \rightarrow 0$. It thus follows from (7.11) and the joint vague-lower semicontinuity of $\mathbb {A}_\textrm{hom}$ (see Lemma 3.14) that

$$\begin{aligned} \mathbb {A}_\textrm{hom}({\varvec{\mu }}, {\varvec{\nu }}) \le \liminf _{\varepsilon \rightarrow 0} \mathcal {A}_\varepsilon ({\pmb {m}}^\varepsilon ). \end{aligned}$$

To conclude the desired estimate $\mathbb {A}_\textrm{hom}({\varvec{\mu }}) \le \liminf _{\varepsilon \rightarrow 0} \mathcal {A}_\varepsilon ({\pmb {m}}^\varepsilon )$, it remains to show that $({\varvec{\mu }}, {\varvec{\nu }})$ solves the continuity equation. To show this, we first note that $(\iota _\varepsilon \widetilde{{\pmb {m}}{}}^\varepsilon ,\iota _\varepsilon \widetilde{\pmb {J}{}}^\varepsilon ) \in \mathbb{C}\mathbb{E}^{\mathcal {I}^\eta }$ in view of Lemma 4.9. It then follows from the weak convergence in (7.10) that $({\varvec{\mu }}_\eta , {\varvec{\nu }}_\eta ) \in \mathbb{C}\mathbb{E}^{\mathcal {I}^\eta }$. Since ${\varvec{\mu }}_\eta \rightarrow {\varvec{\mu }}$, ${\varvec{\nu }}_\eta \rightarrow {\varvec{\nu }}$ vaguely, and $|\mathcal {I}-\mathcal {I}^\eta |\le \eta$ it holds $({\varvec{\mu }}, {\varvec{\nu }}) \in \mathbb{C}\mathbb{E}^\mathcal {I}$, which completes the proof.

### Proof of the discrete regularisation result

This section is devoted to the proof of main discrete regularisation result, Proposition 7.1.

The regularised approximations are constructed by a three-fold regularisation: in time, space, and energy. Let us now describe the relevant operators. Recall the definition of $m^\circ$ and $J^\circ$ as given in Assumption 2.3.

#### Energy regularisation

First we embed $m^\circ$ and $J^\circ$ into the graph $(\mathcal {X}_\varepsilon , \mathcal {E}_\varepsilon )$. We thus define $m^\circ _\varepsilon \in \mathbb {R}_+^{\mathcal {X}_\varepsilon }$ and $J^\circ _\varepsilon \in \mathbb {R}_a^{\mathcal {E}_\varepsilon }$ by

$$\begin{aligned} m^\circ _\varepsilon (\varepsilon z, v) := \varepsilon ^d m^\circ (0,v) \qquad J^\circ _\varepsilon (\varepsilon z, v) := \varepsilon ^{d-1} J^\circ (0,v). \end{aligned}$$

It follows that $(m^\circ _\varepsilon , J^\circ _\varepsilon ) \in {{\,\mathrm{\textsf{D}}\,}}(\mathcal {F}_\varepsilon )^\circ$ (by continuity of $\tau _\varepsilon ^z$, $z \in \mathbb {Z}_\varepsilon ^d$) and

$$\begin{aligned} \mathcal {F}_\varepsilon (m^\circ _\varepsilon , J^\circ _\varepsilon ) = F(m^\circ , J^\circ ). \end{aligned}$$

We then consider the energy regularisation operators defined by

$$\begin{aligned} R_\delta : \mathbb {R}_+^{\mathcal {X}_\varepsilon }&\rightarrow \mathbb {R}_+^{\mathcal {X}_\varepsilon },&R_\delta m&:= (1-\delta ) m + \delta m_\varepsilon ^0, \\ R_\delta : \mathbb {R}_a^{\mathcal {E}_\varepsilon }&\rightarrow \mathbb {R}_a^{\mathcal {E}_\varepsilon },&R_\delta J&:= (1-\delta ) J + \delta J_\varepsilon ^0. \end{aligned}$$

##### Lemma 7.5

(Energy regularisation) Let $\delta \in (0,1)$. The following inequalities hold for any $\varepsilon < \frac{1}{2R_0}$, $m \in \mathbb {R}_+^{\mathcal {X}_\varepsilon }$, and $J \in \mathbb {R}_a^{\mathcal {E}_\varepsilon }$:

$$\begin{aligned} \mathcal {F}_\varepsilon ( R_\delta m, R_\delta J)&\le (1 - \delta ) \mathcal {F}_\varepsilon (m, J) + \delta \mathcal {F}_\varepsilon (m_\varepsilon ^\circ , J_\varepsilon ^\circ ),\\ \Vert R_\delta m \Vert _{\ell ^\infty (\mathcal {X}_\varepsilon )}&\le (1-\delta ) \Vert m \Vert _{\ell ^\infty (\mathcal {X}_\varepsilon )} + \delta \varepsilon ^d \Vert m^\circ \Vert _{\ell ^\infty (\mathcal {X})},\\ \Vert R_\delta J \Vert _{\ell ^\infty (\mathcal {E}_\varepsilon )}&\le (1-\delta ) \Vert J \Vert _{\ell ^\infty (\mathcal {E}_\varepsilon )} + \delta \varepsilon ^{d-1} \Vert J^\circ \Vert _{\ell ^\infty (\mathcal {E})}. \end{aligned}$$

##### Proof

The proof is straightforward consequence of the convexity of *F* and the periodicity of $m^\circ$ and $J^\circ$.

#### Space regularisation

Our space regularisation is a convolution in the *z*-variable with the discretised heat kernel. It is of crucial importance that the regularisation is performed in the $\textsf{z}$-variable only. Smoothness in the $\textsf{v}$-variable is not expected.

For $\lambda > 0$ and $x \in \mathbb {T}^d$, let $h_\lambda (x)$ be the heat kernel on $\mathbb {T}^d$. We consider the discrete version

$$\begin{aligned} H_\lambda ^\varepsilon : \mathbb {Z}_\varepsilon ^d \rightarrow \mathbb {R}, \qquad H_\lambda ^\varepsilon \big ([z]\big ) := \int _{Q_\varepsilon ^z} h_\lambda (x) \, \textrm{d}x, \end{aligned}$$

where the integration ranges over the cube $Q_\varepsilon ^z:= \varepsilon z + [0,\varepsilon )^d \subseteq \mathbb {T}^d$. Using the boundedness and Lipschitz properties of $h_\delta$, we infer that for $z \in \mathbb {Z}_\varepsilon ^d$,

7.12 $$\begin{aligned} \inf _{\mathbb {Z}_\varepsilon ^d} H_\lambda ^\varepsilon&\ge c_\lambda \varepsilon ^d,&\Vert H_\lambda ^\varepsilon \Vert _{\ell ^\infty (\mathbb {Z}_\varepsilon ^d)}&\le C_\lambda \varepsilon ^d, \end{aligned}$$

7.13 $$\begin{aligned} \Vert H_\lambda ^\varepsilon \Vert _{\ell ^1(\mathbb {Z}_\varepsilon ^d)}&= 1,&\big \Vert H_\lambda ^\varepsilon (\cdot + \varepsilon z) - H_\lambda ^\varepsilon \big \Vert _{\ell ^\infty (\mathbb {Z}_\varepsilon ^d)}&\le C_\lambda \varepsilon ^{d+1} |z| \end{aligned}$$

for some non-negative constant $C_\lambda < \infty$ depending only on $\lambda > 0$. We then define

$$\begin{aligned} S_\lambda : \mathbb {R}_+^{\mathcal {X}_\varepsilon }&\rightarrow \mathbb {R}_+^{\mathcal {X}_\varepsilon },&S_\lambda m&:= \sum _{z \in \mathbb {Z}^d_\varepsilon } H_\lambda ^\varepsilon (z) \sigma _\varepsilon ^z m, \\ S_\lambda : \mathbb {R}_a^{\mathcal {E}_\varepsilon }&\rightarrow \mathbb {R}_a^{\mathcal {E}_\varepsilon },&S_\lambda J&:= \sum _{z \in \mathbb {Z}^d_\varepsilon } H_\lambda ^\varepsilon (z) \sigma _\varepsilon ^z J, \end{aligned}$$

where $\sigma _\varepsilon ^z$ is defined in (2.5).

##### Lemma 7.6

(Regularisation in space) Let $\lambda > 0$. There exist constants $c_\lambda > 0$ and $C_\lambda < \infty$ such that the following estimates hold, for any $\varepsilon < \frac{1}{2R_0}$, $m \in \mathbb {R}_+^{\mathcal {X}_\varepsilon }$, $J \in \mathcal {M}^d(\mathcal {E}_\varepsilon )$, and $z \in \mathbb {Z}_\varepsilon ^d$:

(i) Energy bound: $$\displaystyle \mathcal {F}_\varepsilon ( S_\lambda m, S_\lambda J) \le \mathcal {F}_\varepsilon (m, J).$$
(ii) Gain of integrability:
  $$\begin{aligned} \Vert S_\lambda m \Vert _{\ell ^\infty (\mathcal {X}_\varepsilon )} \le C_\lambda \varepsilon ^d \Vert m \Vert _{\ell ^1(\mathcal {X}_\varepsilon )} \quad \text { and }\quad \Vert S_\lambda J \Vert _{\ell ^\infty (\mathcal {E}_\varepsilon )} \le C_\lambda \varepsilon ^d \Vert J \Vert _{\ell ^1(\mathcal {E}_\varepsilon )}. \end{aligned}$$
(iii) Density lower bound: $\displaystyle \inf _{x \in \mathcal {X}_\varepsilon } S_\lambda m(x) \ge c_\lambda \varepsilon ^d \Vert m\Vert _{\ell ^1(\mathcal {X})}.$
(iv) Spatial regularisation:
  $$\begin{aligned} \big \Vert \tau _\varepsilon ^z S_\lambda m - S_\lambda m \big \Vert _{\ell ^\infty (\mathcal {X}_\varepsilon )}&\le C_\lambda \varepsilon ^{d+1} |z| \Vert m \Vert _{\ell ^1(\mathcal {X}_\varepsilon )} \quad \text { and }\quad \\ \big \Vert \tau _\varepsilon ^z S_\lambda J - S_\lambda J \big \Vert _{\ell ^\infty (\mathcal {E}_\varepsilon )}&\le C_\lambda \varepsilon ^{d+1} |z| \Vert J \Vert _{\ell ^1(\mathcal {E}_\varepsilon )}. \end{aligned}$$

##### Proof

Using the convexity of *F* and the identity $\sum _z H_\lambda ^\varepsilon (z) = 1$ we obtain

$$\begin{aligned} \mathcal {F}_\varepsilon ( S_\lambda m, S_\lambda J)&= \sum _{z \in \mathbb {Z}_\varepsilon ^d} \varepsilon ^d F\bigg ( \frac{\tau _\varepsilon ^z S_\lambda m}{\varepsilon ^d}, \frac{\tau _\varepsilon ^z S_\lambda J}{\varepsilon ^{d-1}} \bigg ) \\&\le \sum _{z \in \mathbb {Z}_\varepsilon ^d} \sum _{z' \in \mathbb {Z}_\varepsilon ^d} \varepsilon ^dH_\lambda ^\varepsilon (z') F\bigg ( \frac{\tau _\varepsilon ^{z+z'} m}{\varepsilon ^d}, \frac{\tau _\varepsilon ^{z+z'} J}{\varepsilon ^{d-1}} \bigg ) \\&= \sum _{z \in \mathbb {Z}_\varepsilon ^d} \Big ( \sum _{z' \in \mathbb {Z}_\varepsilon ^d} H_\lambda ^\varepsilon (z-z') \Big ) \varepsilon ^d F\bigg ( \frac{\tau _\varepsilon ^{z} m}{\varepsilon ^d}, \frac{\tau _\varepsilon ^{z} J}{\varepsilon ^{d-1}} \bigg ) = F(M,J), \end{aligned}$$

where in the last equality we used (7.13). This shows (*i*). Properties (*ii*), (*iii*), and (*iv*) are straightforward consequence of the uniform bounds (7.12), (7.13) for the discrete kernels $H_\lambda ^\varepsilon$.

#### Time regularisation

Fix an interval $\mathcal {I}=(a,b) \subset \mathbb {R}$ and a regularisation parameter $\tau >0$. For $({\pmb {m}},\pmb {J}) \in \mathcal{C}\mathcal{E}_\varepsilon ^\mathcal {I}$, we define for $t \in \mathcal {I}_\tau := ( a+\tau , b-\tau )$Note that, thanks to the linearity of the continuity equation we get $(T_\tau {\pmb {m}}, T_\tau \pmb {J}) \in \mathcal{C}\mathcal{E}_\varepsilon ^{\mathcal {I}_\tau }$.

We have the following regularisation properties for the operator $T_\tau$.

##### Lemma 7.7

(Regularisation in time) Let $\tau \in (0, \frac{b-a}{2})$. The following estimates hold for all $\varepsilon < \frac{1}{2R_0}$ and all Borel curves ${\pmb {m}}= (m_t)_{t \in \mathcal {I}} \subseteq \mathbb {R}_+^{\mathcal {X}_\varepsilon }$ and $\pmb {J}= (J_t)_{t \in \mathcal {I}} \subseteq \mathcal {M}^d(\mathcal {E}_\varepsilon )$:

(i) Energy estimate: for some $0 \le C<\infty$ depending only on (2.1) we have
  $$\begin{aligned} \mathcal {A}_\varepsilon ^{\mathcal {I}_\tau }( T_\tau {\pmb {m}}, T_\tau \pmb {J}) \le \mathcal {A}_\varepsilon ({\pmb {m}}, \pmb {J}) + C \tau \big ( {\pmb {m}}(\mathcal {I}\times \mathcal {X}_\varepsilon ) + 1 \big ). \end{aligned}$$
(ii) Mass estimate: $\displaystyle \sup _{t \in \mathcal {I}_\tau } \Vert (T_\tau m)_t \Vert _{\ell ^p(\mathcal {X}_\varepsilon )} \le \sup _{t \in \mathcal {I}} \Vert m_t \Vert _{\ell ^p(\mathcal {X}_\varepsilon )}$.
(iii) Momentum estimate: $\displaystyle \sup _{t \in \mathcal {I}_\tau } \Vert (T_\tau J)_t \Vert _{\ell ^p(\mathcal {X}_\varepsilon )} \le \frac{1}{\tau } \int _\mathcal {I}\Vert J_t \Vert _{\ell ^p(\mathcal {X}_\varepsilon )} \, \textrm{d}t.$
(iv) Time regularity: $\displaystyle \sup _{t \in \mathcal {I}_\tau } \big \Vert \partial _t (T_\tau m)_t \big \Vert _{\ell ^p(\mathcal {X}_\varepsilon )} \le \frac{1}{\tau } \sup _{t \in \mathcal {I}} \Vert m_t \Vert _{\ell ^p(\mathcal {X}_\varepsilon )}$.

##### Proof

Set $w_\tau (s):= (2\tau )^{-1} \big | [(s-\tau ) \vee a,(s+\tau )\wedge b] \big |$ for $s \in \mathcal {I}$. Then we have7.14as a consequence of Jensen’s inequality and Fubini’s theorem. Using that $0 \le w_\tau \le 1$, $\int _{\mathcal {I}} (1-w_\tau (s)) \, \textrm{d}s = 2\tau$, and the growth condition (2.1) we infer

$$\begin{aligned} \int _\mathcal {I}(1-w_\tau (s)) \mathcal {F}_\varepsilon (m_s,J_s) \, \textrm{d}s \ge -C \tau \big ( {\pmb {m}}(\mathcal {I}\times \mathcal {X}_\varepsilon ) + 1 \big ), \end{aligned}$$

which together with (7.14) shows (*i*).

Properties (*ii*), (*iii*) follow directly from the convexity of the $\ell _p$-norms and the subadditivity of the integral.

Finally, (*iv*) follows from the direct computation $\partial _t (T_\tau m)_t = \frac{1}{2\tau } ( m_{t+\tau } - m_{t-\tau } )$.

#### Effects of the three regularisations

We start with a lemma that shows that the effect of the three regularising operators is small if the parameters are small.

Recall the definition of the Kantorovich-Rubinstein norm as given in “Appendix 1”.

##### Lemma 7.8

(Bounds in $\textrm{KR}$-norm) Let $\mathcal {I}\subset \mathbb {R}$ an interval and $(m_t)_{t \in \mathcal {I}} \subseteq \mathbb {R}_+^{\mathcal {X}_\varepsilon }$ be a Borel measurable curve of constant total mass (i.e., $t \mapsto m_t(\mathcal {X}_\varepsilon )$ is constant), and let ${\pmb {m}}\in \mathcal {M}_+(\mathcal {I}\times \mathcal {X}_\varepsilon )$ be the associated measure on space-time defined by ${\pmb {m}}:= \, \textrm{d}t \otimes m_t$. Then there exists a constant $C < \infty$ depending on $|\mathcal {I}|$ such that:

(i) $$ \displaystyle \Vert \iota _\varepsilon T_\tau {\pmb {m}}- \iota _\varepsilon {\pmb {m}}\Vert _{\textrm{KR}(\overline{\mathcal {I}_\tau } \times \mathbb {T}^d)} \le C \tau \sup _{t \in \mathcal {I}} \big \Vert m_t \big \Vert _{\ell ^1(\mathcal {X}_\varepsilon )} $$ for any $$\tau < |\mathcal {I}|/2$$.
(ii) $\displaystyle \Vert \iota _\varepsilon S_\lambda {\pmb {m}}- \iota _\varepsilon {\pmb {m}}\Vert _{\textrm{KR}(\overline{\mathcal {I}} \times \mathbb {T}^d)} \le C \sqrt{\lambda } \sup _{t \in \mathcal {I}} \big \Vert m_t \big \Vert _{\ell ^1(\mathcal {X}_\varepsilon )}$ for any $\lambda > 0$.
(iii) $\displaystyle \Vert \iota _\varepsilon R_\delta {\pmb {m}}- \iota _\varepsilon {\pmb {m}}\Vert _{\textrm{KR}(\overline{\mathcal {I}} \times \mathbb {T}^d)} \le C \delta \Big ( m^\circ (\mathcal {X}^Q) + \sup _{t \in \mathcal {I}} \big \Vert m_t \big \Vert _{\ell ^1(\mathcal {X}_\varepsilon )} \Big )$ for any $\delta \in (0,1)$.

##### Proof

(*i*): For any ${\varvec{\mu }}\in \mathcal {M}(\mathcal {I}\times \mathbb {T}^d)$ and any Lipschitz function $\varphi : \overline{\mathcal {I}_\tau } \times \mathbb {T}^d \rightarrow \mathbb {R}$ (and, in fact, for any temporally Lipschitz function) we haveSince $\iota _\varepsilon {\pmb {m}}\big (\mathcal {I}\times \mathbb {T}^d\big ) \le |\mathcal {I}| \sup _{t \in \mathcal {I}} \big \Vert m_t \big \Vert _{\ell ^1(\mathcal {X}_\varepsilon )}$ we obtain the result.

(*ii*): In view of mass-preservation, we have

$$\begin{aligned} \Vert \iota _\varepsilon S_\lambda {\pmb {m}}- \iota _\varepsilon {\pmb {m}}\Vert _{\textrm{KR}(\overline{\mathcal {I}} \times \mathbb {T}^d)} \le&\int _{\mathcal {I}} \big \Vert \iota _\varepsilon S_\lambda m_t - \iota _\varepsilon m_t \big \Vert _{\textrm{KR}(\mathbb {T}^d)} \, \textrm{d}t \\ \le&\ \sup _{t\in \mathcal {I}} m_t(\mathcal {X}_{\varepsilon }) \int _{\mathcal {I}} \big \Vert \iota _\varepsilon H_\lambda - \iota _\varepsilon H_0 \big \Vert _{\textrm{KR}(\mathbb {T}^d)} \, \textrm{d}t \\ \le&C \sqrt{\lambda } \sup _{t\in \mathcal {I}} m_t(\mathcal {X}_\varepsilon ). \end{aligned}$$

Here in the last inequality we used scaling law of the heat kernel.

(*iii*): Let us write ${\pmb {m}}_\varepsilon ^\circ := \, \textrm{d}t \otimes m_\varepsilon ^\circ$ for brevity. By linearity, we have

$$\begin{aligned} \Vert \iota _\varepsilon (R_\delta {\pmb {m}}- {\pmb {m}}) \Vert _{\textrm{KR}(\overline{\mathcal {I}} \times \mathbb {T}^d)}&= \delta \Vert \iota _\varepsilon ({\pmb {m}}_\varepsilon ^\circ - {\pmb {m}}) \Vert _{\textrm{KR}(\overline{\mathcal {I}} \times \mathbb {T}^d)} \\&\le \delta (1 + |\mathcal {I}|) \Big ( {\pmb {m}}_\varepsilon ^\circ \big (\mathcal {I}\times \mathbb {T}_\varepsilon ^d\big ) + {\pmb {m}}\big (\mathcal {I}\times \mathbb {T}_\varepsilon ^d\big ) \Big ) \\ {}&\le \delta |\mathcal {I}| (1 + |\mathcal {I}|) \Big ( m^\circ (\mathcal {X}^Q) + \sup _{t \in \mathcal {I}} m_t(\mathcal {X}_\varepsilon ) \Big ). \end{aligned}$$

##### Proof of Proposition 7.1

We define

$$\begin{aligned} \widetilde{{\pmb {m}}} := \Big ( R_\delta \circ S_\lambda \circ T_\tau \Big ) {\pmb {m}}\quad \text { and }\quad \widetilde{\pmb {J}} := \Big ( R_\delta \circ S_\lambda \circ T_\tau \Big ) \pmb {J}. \end{aligned}$$

We will show that the desired inequalities hold if $\delta , \lambda , \tau > 0$ are chosen to be sufficiently small, depending on the desired accuracy $\eta > 0$. Set $\mathcal {I}_\tau :=(\tau ,T-\tau )$.

(*i*): We use the shorthand notation $\textrm{KR}_\tau := \textrm{KR}(\overline{\mathcal {I}}_\tau \times \mathbb {T}^d)$. Using Lemma 7.8 we obtain

7.15 $$\begin{aligned} \begin{aligned} \Vert \iota _\varepsilon {\pmb {m}}- \iota _\varepsilon \widetilde{{\pmb {m}}} \Vert _{\textrm{KR}_\tau }&\le \Vert \iota _\varepsilon {\pmb {m}}- \iota _\varepsilon T_\tau {\pmb {m}}\Vert _{\textrm{KR}_\tau } + \Vert \iota _\varepsilon T_\tau {\pmb {m}}- \iota _\varepsilon (S_\lambda T_\tau ) {\pmb {m}}\Vert _{\textrm{KR}_\tau } \\ {}&\qquad + \Vert \iota _\varepsilon (S_\lambda T_\tau ) {\pmb {m}}- \iota _\varepsilon (R_\delta S_\lambda T_\tau ) {\pmb {m}}\Vert _{\textrm{KR}_\tau } \\ {}&\lesssim M( \tau + \sqrt{\lambda } + \delta ) + m^\circ (\mathcal {X}^Q) \delta . \end{aligned} \end{aligned}$$

Furthermore, using Lemma 7.5, Lemma 7.6(i), and Lemma 7.7(i) we obtain the action bound

7.16 $$\begin{aligned} \begin{aligned} \mathcal {A}_\varepsilon ^{\mathcal {I}_\tau }(\widetilde{{\pmb {m}}}, \widetilde{\pmb {J}})&= \mathcal {E}_\varepsilon \Big ( ( R_\delta \circ S_\lambda \circ T_\tau ) {\pmb {m}}, ( R_\delta \circ S_\lambda \circ T_\tau ) \pmb {J}\Big ) \\ {}&\le (1 - \delta ) \mathcal {A}_\varepsilon \Big ( ( S_\lambda \circ T_\tau ) {\pmb {m}}, ( S_\lambda \circ T_\tau ) \pmb {J}\Big ) + \delta T \mathcal {F}_\varepsilon (m_\varepsilon ^\circ , J_\varepsilon ^\circ ) \\ {}&\le (1 - \delta ) \mathcal {A}_\varepsilon ( {\pmb {m}}, \pmb {J}) + \delta T \mathcal {F}(m^\circ , J^\circ ) + C \tau (M+1). \end{aligned} \end{aligned}$$

The desired inequalities (7.1) follow by choosing $\delta$, $\lambda$, and $\tau$ sufficiently small.

(*ii*): We will show that all the estimates hold with constants depending on $\eta$ through the parameters $\delta$, $\lambda$, and $\tau$.

*Boundedness*: We apply Lemma 7.5, Lemma 7.6(ii), and Lemma 7.7(ii) &(iii) and obtain the uniform bounds on the mass

7.17 $$\begin{aligned} \begin{aligned} \sup _{t \in \mathcal {I}_\tau } \Vert \widetilde{m}_t \Vert _{\ell ^\infty (\mathcal {X}_\varepsilon )}&\le \varepsilon ^d \bigg ( (1-\delta ) C_\lambda \sup _{t \in [0,T]} \Vert m_t \Vert _{\ell ^1(\mathcal {X}_\varepsilon )} + \delta \Vert m^\circ \Vert _{\ell ^\infty (\mathcal {X}_\varepsilon )} \bigg ), \\&\le \varepsilon ^d \bigg ( C_\lambda M + \delta \Vert m^\circ \Vert _{\ell ^\infty (\mathcal {X}^Q)} \bigg ) \end{aligned} \end{aligned}$$

as well as the uniform bounds on the momentum

7.18 $$\begin{aligned} \begin{aligned} \sup _{t \in \mathcal {I}_\tau } \Vert \widetilde{J}_t \Vert _{\ell ^\infty (\mathcal {X}_\varepsilon )}&\le \varepsilon ^{d-1} \bigg ( \frac{1 - \delta }{\tau }C_\lambda \sup _{t \in [0,T]} \int _\mathcal {I}\varepsilon \Vert J_t \Vert _{\ell ^1(\mathcal {X}_\varepsilon )} \, \textrm{d}t + \delta \Vert J^\circ \Vert _{\ell ^\infty (\mathcal {X}_\varepsilon )} \bigg ), \\ {}&\lesssim \varepsilon ^{d-1} \bigg ( \frac{C_\lambda }{\tau } \Big ( T (1 + M) + E \Big ) + \delta \Vert J^\circ \Vert _{\ell ^\infty (\mathcal {E}^Q)} \bigg ). \end{aligned} \end{aligned}$$

*Time-regularity*: From Lemma 7.7(iv), together with Lemma 7.5 and Lemma 7.6(ii), we obtain the uniform bound on the time derivative

7.19 $$\begin{aligned} \begin{aligned} \sup _{t \in \mathcal {I}_\tau } \Vert \partial _t \widetilde{m}_t \Vert _{\ell ^\infty (\mathcal {X}_\varepsilon )}&\le \varepsilon ^d \bigg ( 2 \frac{1-\delta }{\tau } C_\lambda \sup _{t \in [0,T]} \Vert m_t \Vert _{\ell ^1(\mathcal {X}_\varepsilon )} + \delta \Vert m^\circ \Vert _{\ell ^\infty (\mathcal {X}_\varepsilon )} \bigg ), \\ {}&\le \varepsilon ^d \bigg ( 2 \frac{C_\lambda }{\tau } M + \delta \Vert m^\circ \Vert _{\ell ^\infty (\mathcal {X}^Q)} \bigg ). \end{aligned} \end{aligned}$$

*Space-regularity*: For $z,z' \in \mathbb {Z}_\varepsilon ^d$ and $v \in V$, Lemma 7.6(iv) and Lemma 7.7(ii) yield

$$\begin{aligned} | \widetilde{m}_t(z,v) - \widetilde{m}_t(z',v) |&\le (1-\delta ) \big | \big (S_\lambda \circ T_\tau \big )m_t(z,v) - \big (S_\lambda \circ T_\tau \big )m_t(z',v) \big | \\ {}&\le C_\lambda \varepsilon ^{d-1} |z-z'| \big \Vert T_\tau m_t \big \Vert _{\ell ^1(\mathcal {X}_\varepsilon )} \\ {}&\le C_\lambda \varepsilon ^{d+1} |z-z'| \sup _{t \in [0,T]} \big \Vert m_t \big \Vert _{\ell ^1(\mathcal {X}_\varepsilon )}, \end{aligned}$$

which shows that

7.20 $$\begin{aligned} \begin{aligned} \sup _{t\in \mathcal {I}_\tau } \Vert \sigma _\varepsilon ^z \widetilde{m}_t - \widetilde{m}_t \Vert _{\ell ^\infty (\mathcal {X}_\varepsilon )}&\le C_\lambda \varepsilon ^{d+1} |z| \sup _{t \in [0,T]} \big \Vert m_t \big \Vert _{\ell ^1(\mathcal {X}_\varepsilon )} \le C_\lambda \varepsilon ^{d+1} |z| M. \end{aligned} \end{aligned}$$

Similarly, using the growth condition (2.1) we deduce

7.21 $$\begin{aligned} \begin{aligned} \sup _{t\in \mathcal {I}_\tau } \Vert \sigma _\varepsilon ^z \widetilde{J}_t - \widetilde{J}_t \Vert _{\ell ^\infty (\mathcal {E}_\varepsilon )}&\le \frac{C_\lambda }{\tau } \varepsilon ^{d+1} |z| \int _\mathcal {I}\big \Vert J_s \big \Vert _{\ell ^1(\mathcal {E}_\varepsilon )} \, \textrm{d}s \\ {}&\le \frac{C_\lambda }{\tau } \varepsilon ^{d} |z| \Big ( T (1 + M) + E \Big ). \end{aligned} \end{aligned}$$

*Domain-regularity*: For all $t \in \mathcal {I}_\tau$, reasoning as in (7.17) and (7.18), we observe that

$$\begin{aligned} \varepsilon ^{-d} \Vert (S_\lambda T_\tau m)_t \Vert _{\ell ^\infty (\mathcal {X}_\varepsilon )} \le C_\lambda \Vert (T_\tau m)_t \Vert _{\ell ^1(\mathcal {X}_\varepsilon )} \le C_\lambda \sup _{t \in [0,T]} \Vert m_t \Vert _{\ell ^1(\mathcal {X}_\varepsilon )} \le C_\lambda M,\\ \varepsilon ^{-d} \Vert (S_\lambda T_\tau J)_t \Vert _{\ell ^\infty (\mathcal {E}_\varepsilon )} \le C_\lambda \Vert (T_\tau m)_t \Vert _{\ell ^1(\mathcal {E}_\varepsilon )} \le \frac{C_\lambda }{\tau } \int _\mathcal {I}\Vert J_t \Vert _{\ell ^1(\mathcal {E}_\varepsilon )} \, \textrm{d}t \le \frac{C_\lambda }{\tau \varepsilon } \Big ( T (1 + M) + E \Big ). \end{aligned}$$

We infer that

$$\begin{aligned} \bigg \Vert \frac{\tau _\varepsilon ^z (S_\lambda T_\tau m)_t }{\varepsilon ^d} \bigg \Vert _{\ell ^\infty (\mathcal {X})} \le C_\lambda M \quad \text {and} \quad \bigg \Vert \frac{\tau _\varepsilon ^z (S_\lambda T_\tau J)_t }{\varepsilon ^{d-1}} \bigg \Vert _{\ell ^\infty (\mathcal {E})} \le \frac{C_\lambda }{\tau } \Big ( T (1 + M) + E \Big ) \end{aligned}$$

Since

$$\begin{aligned} \bigg (\frac{\tau _\varepsilon ^z \widetilde{m}_t}{\varepsilon ^d} , \frac{\tau _\varepsilon ^z \widetilde{J}_t}{\varepsilon ^{d-1}} \bigg ) = (1-\delta ) \bigg ( \frac{\tau _\varepsilon ^z (S_\lambda T_\tau m)_t }{\varepsilon ^d}, \frac{\tau _\varepsilon ^z (S_\lambda T_\tau J)_t }{\varepsilon ^{d-1}} \bigg ) + \delta (m^\circ , J^\circ ), \end{aligned}$$

the claim follows by an application of Lemma C.1 to the product of balls in ${\ell ^\infty (\mathcal {X})}$ and ${\ell ^\infty (\mathcal {E})}$, taking into account that *F* is defined on a finite-dimensional subspace by the locality assumption.

## Proof of the upper bound

In this section we present the proof of the $\Gamma$-limsup inequality in Theorem 5.1. The first step is to introduce the notion of *optimal microstructures*.

### The optimal discrete microstructures

Let $\mathcal {I}$ be an open interval in $\mathbb {R}$. We will make use of the following canonical discretisation of measures and vector fields on the cartesian grid $\mathbb {Z}_\varepsilon ^d$.

#### Definition 8.1

($\mathbb {Z}_\varepsilon ^d$-discretisation of measures) Let $\mu \in \mathcal {M}_+(\mathbb {T}^d)$ and $\nu \in \mathcal {M}^d(\mathbb {T}^d)$ have continuous densities $\rho$ and *j*, respectively, with respect to the Lebesgue measure. Their $\mathbb {Z}_\varepsilon ^d$-discretisations $\textsf{P}_\varepsilon \mu : \mathbb {Z}_\varepsilon ^d \rightarrow \mathbb {R}_+$ and $\textsf{P}_\varepsilon \nu : \mathbb {Z}_\varepsilon ^d \rightarrow \mathbb {R}^d$ are defined by

$$\begin{aligned} \textsf{P}_\varepsilon \mu (z):=\mu (Q_\varepsilon ^z), \quad \textsf{P}_\varepsilon \nu (z):=\left( \int _{\partial Q_\varepsilon ^z\cap \partial Q_\varepsilon ^{z+ e_i}}j\cdot e_i\, \textrm{d}\mathcal {H}^{d-1}\right) _{i=1}^d. \end{aligned}$$

An important feature of this discretisation is the preservation of the continuity equation, in the following sense.

#### Definition 8.2

(Continuity equation on $\mathbb {Z}_\varepsilon ^d$) Fix $\mathcal {I}\subset \mathbb {R}$ an open interval. We say that $\pmb {r}: \mathcal {I}\times \mathbb {Z}_\varepsilon ^d \rightarrow \mathbb {R}_+$ and $\pmb {u}: \mathcal {I}\times \mathbb {Z}_\varepsilon ^d \rightarrow \mathbb {R}^d$ satisfy the continuity equation on $\mathbb {Z}_\varepsilon ^d$, and write $(\pmb {r}, \pmb {u}) \in \textsf{CE}_{\varepsilon ,d}^\mathcal {I}$, if $\pmb {r}$ is continuous, $\pmb {u}$ is Borel measurable, and the following discrete continuity equation is satisfied in the sense of distributions:

8.1 $$\begin{aligned} \partial _t r_t(z) + \sum _{i=1}^d \big ( u_t(z) - u_t(z - e_i) \big ) \cdot e_i = 0, \quad \text {for } z \in \mathbb {Z}^d_\varepsilon . \end{aligned}$$

#### Lemma 8.3

(Discrete continuity equation on $\mathbb {Z}_\varepsilon ^d$) Let $({\varvec{\mu }},{\varvec{\nu }}) \in \mathbb{C}\mathbb{E}^\mathcal {I}$ have continuous densities with respect to the space-time Lebesgue measure on $\mathcal {I}\times \mathbb {T}^d$. Then $(\textsf{P}_\varepsilon {\varvec{\mu }}, \textsf{P}_\varepsilon {\varvec{\nu }}) \in \textsf{CE}_{\varepsilon ,d}^\mathcal {I}$.

#### Proof

This follows readily from the Gauss divergence theorem.

The key idea of the proof of the upper bound in Theorem 5.1 is to start from a (smooth) solution to the continuous equation $\mathbb{C}\mathbb{E}^\mathcal {I}$, and to consider the optimal discrete microstructure of the mass and the flux in each cube $Q_\varepsilon ^z$. The global candidate is then obtained by gluing together the optimal microstructures *cube by cube*.

We start defining the *gluing operator*. Recall the operator $T_\varepsilon ^0$ defined in (2.4).

#### Definition 8.4

(Gluing operator) Fix $\varepsilon > 0$. For each $z \in \mathbb {Z}_\varepsilon ^d$, let

$$\begin{aligned} m^z \in \mathbb {R}_+^{\mathcal {X}} \quad \text { and }\quad J^z \in \mathbb {R}_a^{\mathcal {E}} \end{aligned}$$

be $\mathbb {Z}^d$-periodic. The *gluings* of $m = (m^z)_{z \in \mathbb {Z}_\varepsilon ^d}$ and $J = (J^z)_{z \in \mathbb {Z}_\varepsilon ^d}$ are the functions $\mathcal {G}_\varepsilon m \in \mathbb {R}_+^{\mathcal {X}_\varepsilon }$ and $\mathcal {G}_\varepsilon J \in \mathbb {R}_a^{\mathcal {E}_\varepsilon }$ defined by

8.2 $$\begin{aligned} \begin{aligned} \mathcal {G}_\varepsilon m \big ( T_\varepsilon ^0(x) \big )&:= m^{ x_\textsf{z}}(x){} & {} \text {for } x \in \mathcal {X}, \\ \mathcal {G}_\varepsilon J \big ( T_\varepsilon ^0(x), T_\varepsilon ^0(y) \big )&:= \frac{1}{2} \Big ( J^{x_\textsf{z}}(x,y) + J^{y_\textsf{z}}(x,y) \Big ){} & {} \text {for } (x,y) \in \mathcal {E}. \end{aligned} \end{aligned}$$

#### Remark 8.5

(Well-posedness) Note that $\mathcal {G}_\varepsilon m$ and $\mathcal {G}_\varepsilon J$ are well-defined thanks to the $\mathbb {Z}_\varepsilon ^d$-periodicity of the functions $m^z$ and $J^z$.

#### Remark 8.6

(Mass preservation and KR-bounds) The gluing operation is locally mass-preserving in the following sense. Let $\mu \in \mathcal {M}_+(\mathbb {T}^d)$ and consider a family of measures $m = (m^z)_{z \in \mathbb {Z}_\varepsilon ^d} \subseteq \mathbb {R}_+^{\mathcal {X}}$ satisfying $m^z \in {{\,\mathrm{{\textsf{Rep}}}\,}}\big ( \textsf{P}_\varepsilon \mu (z) \big )$ for some $z \in \mathbb {Z}_\varepsilon ^d$. Then:

$$\begin{aligned} \mathcal {G}_\varepsilon m \Big ( \mathcal {X}_\varepsilon \cap \{ x_\textsf{z}= z\} \Big ) = \mu (Q_\varepsilon ^z) \end{aligned}$$

for every $\varepsilon > 0$. Consequently,

8.3 $$\begin{aligned} \Vert \iota _\varepsilon \mathcal {G}_\varepsilon {\pmb {m}}- {\varvec{\mu }}\Vert _{{\textrm{KR}}(\overline{\mathcal {I}} \times \mathbb {T}^d)} \le {\varvec{\mu }}\big (\overline{\mathcal {I}}\times \mathbb {T}^d\big ) \sqrt{d} \varepsilon \end{aligned}$$

for all weakly continuous curves ${\varvec{\mu }}= (\mu _t)_{t \in \overline{\mathcal {I}}} \subseteq \mathcal {M}_+(\mathbb {T}^d)$ and all ${\pmb {m}}= (m_t^z)_{t \in \overline{\mathcal {I}}, z \in \mathbb {Z}_\varepsilon ^d}$ such that $m_t^z \in {{\,\mathrm{{\textsf{Rep}}}\,}}\big (\textrm{P}_\varepsilon \mu _t(z)\big )$ for all $t \in \overline{\mathcal {I}}$ and $z \in \mathbb {Z}_\varepsilon ^d$.

#### Energy estimates for Lipschitz microstructures

The next lemma shows that the energy of glued measures can be controlled under suitable regularity assumptions.

##### Lemma 8.7

(Energy estimates under regularity) Fix $\varepsilon > 0$. For each $z \in \mathbb {Z}_\varepsilon ^d$, let $m^z \in \mathbb {R}_+^{\mathcal {X}}$ and $J^z \in \mathbb {R}_a^{\mathcal {E}}$ be $\mathbb {Z}^d$-periodic functions satisfying:

(i) (Lipschitz dependence): For all $z, \widetilde{z} \in \mathbb {Z}_\varepsilon ^d$
  $$\begin{aligned} \big \Vert m^z - m^{\widetilde{z}} \big \Vert _{\ell ^\infty (\mathcal {X})} + \varepsilon \big \Vert J^z - J^{\widetilde{z}} \big \Vert _{\ell ^\infty (\mathcal {E})} \le L |z - \widetilde{z}| \varepsilon ^{d+1}. \end{aligned}$$
(ii) (Domain regularity): There exists a compact and convex set $K\Subset {{\,\mathrm{\textsf{D}}\,}}(F)^\circ$ such that, for all $z \in \mathbb {Z}_\varepsilon ^d$,
  8.4 $$\begin{aligned} \bigg ( \frac{m^z}{\varepsilon ^d}, \frac{J^z}{\varepsilon ^{d-1}} \bigg ) \in K. \end{aligned}$$

Then there exists $\varepsilon _0 > 0$ depending only on *K*, *F* such that for $\varepsilon \le \varepsilon _0$

8.5 $$\begin{aligned} \mathcal {F}_\varepsilon \big ( \mathcal {G}_\varepsilon m, \mathcal {G}_\varepsilon J \big ) \le \sum _{z \in \mathbb {Z}_\varepsilon ^d} \varepsilon ^d F \bigg ( \frac{m^z}{\varepsilon ^d}, \frac{J^z}{\varepsilon ^{d-1}} \bigg ) + c \varepsilon , \end{aligned}$$

where $c < \infty$ depends only on *L*, the (finite) Lipschitz constant $\textrm{Lip}(F;K)$, and the locality radius $R_1$.

##### Proof

Fix $\bar{z} \in \mathbb {Z}_\varepsilon ^d$. As *m* is $\mathbb {Z}^d$-periodic, (*i*) yields for $x = (z,v) \in \mathcal {X}_{R_1}$,

8.6 $$\begin{aligned} | \tau _\varepsilon ^{\bar{z}} \mathcal {G}_\varepsilon m(x) - m^{\bar{z}}(x) |&= | m^{\bar{z} + z}(x) - m^{\bar{z}}(x) | \le L R_1 \varepsilon ^{d + 1}, \end{aligned}$$

Similarly, using the $\mathbb {Z}^d$-periodicity of *J*, (*i*) yields for $(x, y) \in \mathcal {E}$ with $x = (z,v) \in \mathcal {X}_{R_1}$ and $y = (\widetilde{z}, \widetilde{v}) \in \mathcal {X}_{R_1}$,

8.7 $$\begin{aligned} | \tau _\varepsilon ^{\bar{z}} \mathcal {G}_\varepsilon J(x, y) - J^{\bar{z}}(x, y) |&= \Big | \Big ( \tfrac{1}{2} J^{\bar{z} + z} + \tfrac{1}{2} J^{\bar{z} + \widetilde{z}} - J^{\bar{z}} \Big )(x,y) \Big | \le L R_1 \varepsilon ^d. \end{aligned}$$

Hence the domain regularity assumption (*ii*) imply a domain regularity property for the glued measures, namely

$$\begin{aligned} \bigg ( \frac{\tau _\varepsilon ^{\bar{z}} \mathcal {G}_\varepsilon m}{\varepsilon ^d}, \frac{\tau _\varepsilon ^{\bar{z}} \mathcal {G}_\varepsilon J}{\varepsilon ^{d-1}} \bigg ) \in \widetilde{K} \end{aligned}$$

for all ${\bar{z}} \in \mathbb {Z}_\varepsilon ^d$ and $\varepsilon \le \varepsilon _0:=\frac{1}{2}\text {dist}(K, \partial {{\,\mathrm{\textsf{D}}\,}}(F)) \in (0,+\infty )$, where $\widetilde{K} \Subset {{\,\mathrm{\textsf{D}}\,}}(F)^\circ$ is a slightly bigger compact set than *K*.

Consequently, we can use the Lipschitzianity of *F* on the compact set $\widetilde{K}$ and its locality to estimate the energy as

$$\begin{aligned}&\left| F \bigg ( \frac{\tau _\varepsilon ^{\bar{z}} \mathcal {G}_\varepsilon m}{\varepsilon ^d}, \frac{\tau _\varepsilon ^{\bar{z}} \mathcal {G}_\varepsilon J }{\varepsilon ^{d-1}} \bigg ) - F \bigg ( \frac{M^{\bar{z}}}{\varepsilon ^d}, \frac{J^{\bar{z}}}{\varepsilon ^{d-1}} \bigg ) \right| \\ {}&\le \textrm{Lip}(F;\widetilde{K}) \bigg ( \frac{ \Vert \tau _\varepsilon ^{\bar{z}} \mathcal {G}_\varepsilon m - m^{\bar{z}} \Vert _{\ell ^\infty (\mathcal {X}_{R_1})}}{\varepsilon ^d} + \frac{ \Vert \tau _\varepsilon ^{\bar{z}} \mathcal {G}_\varepsilon J - J^{\bar{z}} \Vert _{\ell ^\infty (\mathcal {E}_{R_1})}}{\varepsilon ^{d-1}} \bigg ), \end{aligned}$$

where $$\mathcal {X}_R:= \{x \in \mathcal {X}\, \ |x|_{\ell _\infty ^d} \le R\}$$ and $$\mathcal {E}_R:= \{(x,y) \in \mathcal {E}\, \ |x|_{\ell _\infty ^d}, |y|_{\ell _\infty ^d} \le R\}$$.

Combining the estimate above with (8.6) and (8.7), we conclude that

$$\begin{aligned}&\left| F \bigg ( \frac{\tau _\varepsilon ^{\bar{z}} \mathcal {G}_\varepsilon m}{\varepsilon ^d}, \frac{\tau _\varepsilon ^{\bar{z}} \mathcal {G}_\varepsilon J }{\varepsilon ^{d-1}} \bigg ) - F \bigg ( \frac{M^{\bar{z}}}{\varepsilon ^d}, \frac{J^{\bar{z}}}{\varepsilon ^{d-1}} \bigg ) \right| \le 2 L R_1 \textrm{Lip}(F;\widetilde{K}) \varepsilon . \end{aligned}$$

for $\varepsilon \le \varepsilon _0$. Summation over $\bar{z} \in \mathbb {Z}_\varepsilon ^d$ yields the desired estimate (8.5).

We now introduce the notion of *optimal microstructure* associated with a pair of measures $(\mu ,\nu ) \in \mathcal {M}_+(\mathbb {T}^d) \times \mathcal {M}^d(\mathbb {T}^d)$. First, let us define regular measures.

##### Definition 8.8

(Regular measures) We say that $(\mu ,\nu ) \in \mathcal {M}_+(\mathbb {T}^d) \times \mathcal {M}^d(\mathbb {T}^d)$ is a *regular pair of measures* if the following properties hold:

(i) (Lipschitz regularity): With respect to the Lebesgue measure on $\mathbb {T}^d$, the measures $\mu$ and $\nu$ have Lipschitz continuous densities $\rho$ and *j* respectively.
(ii) (Compact inclusion): There exists a compact set $\widetilde{K} \Subset {{\,\mathrm{\textsf{D}}\,}}(f_\textrm{hom})^\circ$ such that
  $$\begin{aligned} \big (\rho (x),j(x)\big ) \in \widetilde{K} \quad \text { for all } x \in \mathbb {T}^d. \end{aligned}$$

We say that $(\mu _t, \nu _t)_{t \in \mathcal {I}} \subseteq \mathcal {M}_+(\mathbb {T}^d) \times \mathcal {M}^d(\mathbb {T}^d)$ is a *regular curve of measures* if $(\mu _t,\nu _t)$ are regular measures for every $t \in \mathcal {I}$ and $t \mapsto (\rho _t(x), j_t(x))$ is measurable for every $x \in \mathbb {T}^d$.

##### Definition 8.9

(Optimal microstructure) Let $(\mu , \nu ) \in \mathcal {M}_+(\mathbb {T}^d) \times \mathcal {M}^d(\mathbb {T}^d)$ be a regular pair of measures.

(i) We say that $(m^z, J^z)_{z \in \mathbb {Z}_\varepsilon ^d} \subseteq \mathbb {R}_+^\mathcal {X}\times \mathbb {R}_a^\mathcal {E}$ is an *admissible microstructure* for $(\mu , \nu )$ if
  $$\begin{aligned} (m^z, J^z) \in {{\,\mathrm{{\textsf{Rep}}}\,}}\bigg ( \frac{\textsf{P}_\varepsilon \mu (z)}{\varepsilon ^d}, \frac{\textsf{P}_\varepsilon \nu (z)}{\varepsilon ^{d-1}} \bigg ) \end{aligned}$$
  for every $$z \in \mathbb {Z}_\varepsilon ^d$$.
(ii) If, additionally, $(m^z, J^z) \in {{\,\mathrm{{\textsf{Rep}}}\,}}_o \Big ( \frac{\textsf{P}_\varepsilon \mu (z)}{\varepsilon ^d}, \frac{\textsf{P}_\varepsilon \nu (z)}{\varepsilon ^{d-1}} \Big )$ for every $z \in \mathbb {Z}_\varepsilon ^d$, we say that $(m^z, J^z)_{z \in \mathbb {Z}_\varepsilon ^d}$ is an *optimal microstructure* for $(\mu , \nu )$.

##### Remark 8.10

(Measurable dependence) If $t \mapsto (\mu _t,\nu _t)$ is a measurable curve in $\mathcal {M}_+(\mathbb {T}^d) \times \mathcal {M}^d(\mathbb {T}^d)$, it is possible to select a collection of admissible (resp. optimal) microstructures that depend measurably on *t*. This follows from Lemma 4.7; see e.g. [38, Theorem 14.37]. In the sequel, we will always work with measurable selections.

The next proposition shows that each optimal microstructures associated with a regular pair of measures $(\mu , \nu )$ has discrete energy which can be controlled by the homogenised continuous energy $\mathbb {F}_\textrm{hom}(\mu ,\nu )$.

##### Proposition 8.11

(Energy bound for optimal microstructures) Let $(m^z, J^z)_{z \in \mathbb {Z}_\varepsilon ^d} \subseteq \mathbb {R}_+^\mathcal {X}\times \mathbb {R}_a^\mathcal {E}$ be an optimal microstructure for a regular pair of measures $(\mu , \nu ) \in \mathcal {M}_+(\mathbb {T}^d) \times \mathcal {M}^d(\mathbb {T}^d)$. Then:

$$\begin{aligned} \sum _{z \in \mathbb {Z}_\varepsilon ^d} \varepsilon ^d F \left( \frac{ m^z}{\varepsilon ^d}, \frac{ J^z}{\varepsilon ^{d-1}} \right) \le \mathbb {F}_{\textrm{hom}}(\mu , \nu ) + C \varepsilon , \end{aligned}$$

where $C < \infty$ depends only on $\textrm{Lip}(f_\textrm{hom}; \widetilde{K})$ and the modulus of continuity of the densities $\rho$ and *j* of $\mu$ and $\nu$.

##### Proof

Let us denote the densities of $\mu$ and $\nu$ by $\rho$ and *j* respectively. Using the regularity of ${\varvec{\mu }}$ and ${\varvec{\nu }}$, and the fact that $f_\textrm{hom}$ is Lipschitz on $\widetilde{K}$, we obtain

$$\begin{aligned} \sum _{z \in \mathbb {Z}_\varepsilon ^d} \varepsilon ^d F \left( \frac{ m^z}{\varepsilon ^d}, \frac{ J^z}{\varepsilon ^{d-1}} \right)&= \sum _{z \in \mathbb {Z}_\varepsilon ^d} \varepsilon ^d f_\textrm{hom}\bigg ( \frac{\textsf{P}_\varepsilon \mu (z)}{\varepsilon ^d}, \frac{\textsf{P}_\varepsilon \nu (z)}{\varepsilon ^{d-1}} \bigg ) \le \int _{\mathbb {T}^d} f_\textrm{hom}(\rho _t(a), j_t(a)) \, \textrm{d}a + C \varepsilon , \end{aligned}$$

which is the desired estimate.

##### Remark 8.12

(Lack of regularity) Suppose that $\widehat{m}:= \mathcal {G}_\varepsilon m$ and $\widehat{J}:= \mathcal {G}_\varepsilon J$ are constructed by gluing the optimal microstructure $(m,J) = (m^z, J^z)_{z\in \mathbb {Z}_\varepsilon ^d}$ from the previous lemma. It is then tempting to seek for an estimate of the formHowever, (*m*, *J*) does not have the required *a priori* regularity estimates to obtain such a bound. Moreover, the gluing procedure does not necessarily produce solutions to the discrete continuity equation if we start with solutions to the continuous continuity equation.

We conclude the subsection with the following $L^1$ and $L^\infty$ estimates.

##### Lemma 8.13

($L^1$ and $L^\infty$ estimates) Let $(\mu _t,\nu _t)_{t\in \mathcal {I}} \subset \mathcal {M}_+(\mathbb {T}^d) \times \mathcal {M}^d(\mathbb {T}^d)$ be a regular curve of measures satisfying

8.8 $$\begin{aligned} M := \sup _{t \in \mathcal {I}} \mu _t(\mathbb {T}^d)< \infty \quad \text { and }\quad A := \mathbb {A}_\textrm{hom}^\mathcal {I}({\varvec{\mu }},{\varvec{\nu }}) < \infty . \end{aligned}$$

Let $(m_t^z, J_t^z)_{z \in \mathbb {Z}_\varepsilon ^d} \subseteq \mathcal {M}_+(\mathbb {T}^d) \times \mathcal {M}^d(\mathbb {T}^d)$ be corresponding optimal microstructures. Then:

(i) $(\textsf{P}_\varepsilon \mu _, \textsf{P}_\varepsilon \nu )$ satisfies the uniform estimate
  8.9 $$\begin{aligned} \sup _{\varepsilon >0} \sup _{t \in \mathcal {I}} \Vert \textsf{P}_\varepsilon \mu _t \Vert _{\ell ^1(\mathbb {Z}_\varepsilon ^d)} = M. \end{aligned}$$
(ii) $(m_t, J_t)_{t \in \mathcal {I}}$ satisfies the uniform estimate
  8.10 $$\begin{aligned}&\sup _{\varepsilon >0} \sup _{(t,x) \in \mathcal {I}\times \mathcal {X}} \sum _{z \in \mathbb {Z}_\varepsilon ^d} m_t^z(x) \le M \end{aligned}$$
  8.11 $$\begin{aligned}&\sup _{\varepsilon >0} \sup _{(x,y) \in \mathcal {E}} \varepsilon \int _\mathcal {I}\sum _{z \in \mathbb {Z}_\varepsilon ^d} \big | J_t^z(x,y) \big | \, \textrm{d}t \lesssim A + M. \end{aligned}$$

##### Proof

The first claim follows since $\Vert \textsf{P}_\varepsilon \mu _t \Vert _{\ell ^1(\mathbb {Z}_\varepsilon ^d)} = \mu _t(\mathbb {T}^d)$ by construction.

To prove (*ii*), note that

$$\begin{aligned} \sum _{z \in \mathbb {Z}_\varepsilon ^d} \sum _{x \in \mathcal {X}^Q} m_t^z(x) = \sum _{z \in \mathbb {Z}_\varepsilon ^d} \textsf{P}_\varepsilon \mu (z) = \mu _t(\mathbb {T}^d), \end{aligned}$$

which yields (8.10).

To prove (8.11), we use the growth condition on *F*, the periodicity of $J_t^z$, and (*i*) to obtain for $(x,y)\in \mathcal {E}$ and $t \in \mathcal {I}$:

$$\begin{aligned} \varepsilon \sum _{z \in \mathbb {Z}_\varepsilon ^d} \big |J_t^z(x,y) \big | \le \sum _{z \in \mathbb {Z}_\varepsilon ^d} \varepsilon ^d \sum _{(\widetilde{x} , \widetilde{y}) \in \mathcal {E}^Q} \bigg | \frac{J_t^z(\widetilde{x} , \widetilde{y})}{\varepsilon ^{d-1}} \bigg |&\lesssim \sum _{z \in \mathbb {Z}_\varepsilon ^d} \varepsilon ^d F \Big ( \frac{m_t^z}{\varepsilon ^d},\frac{J_t^z}{\varepsilon ^{d-1}} \Big ) + M \\&\lesssim \int _{\mathbb {T}^d} f_\textrm{hom}\Big ( \frac{\, \textrm{d}\mu _t}{\, \textrm{d}x},\frac{\, \textrm{d}j_t}{\, \textrm{d}x}\Big ) \, \textrm{d}x + M, \end{aligned}$$

where in the last inequality we applied Proposition 8.11. Integrating in time and taking the supremum in space and $\varepsilon >0$, we obtain (8.11).

### Approximation result

The goal of this subsection is to show that despite the issues outlined in Remark 8.12, we can find a solution to $\mathcal{C}\mathcal{E}_\varepsilon ^\mathcal {I}$ with almost optimal energy that is $\Vert \cdot \Vert _{\textrm{KR}}$-close to a glued optimal microstructure.

In the following result, $\mathcal {I}_\eta = (a - \eta , b + \eta )$ denotes the $\eta$-extension of the open interval $\mathcal {I}= (a,b)$ for $\eta > 0$.

#### Proposition 8.14

(Approximation of optimal microstructures) Let $({\varvec{\mu }}, {\varvec{\nu }}) \in \mathbb{C}\mathbb{E}^{\mathcal {I}_\eta }$ be a regular curve of measures sastisfying

$$\begin{aligned} M := \mu _0(\mathbb {T}^d)< \infty \quad \text { and }\quad A := \mathbb {A}_{\textrm{hom}}^{\mathcal {I}_\eta }({\varvec{\mu }}, {\varvec{\nu }}) < \infty . \end{aligned}$$

Let $(m_t^z, J_t^z)_{t \in \mathcal {I}, z \in \mathbb {Z}_\varepsilon ^d} \subseteq \mathbb {R}_+^{\mathcal {X}} \times \mathbb {R}_a^{\mathcal {E}}$ be a measurable family of optimal microstructures associated to $(\mu _t, \nu _t)_{t \in \mathcal {I}}$ and consider their gluing $(\widehat{m}_t, \widehat{J}_t)_{t \in \mathcal {I}} \subseteq \mathbb {R}_+^{\mathcal {X}_\varepsilon } \times \mathbb {R}_a^{\mathcal {E}_\varepsilon }$. Then, for every $\eta ' > 0$, there exists $\varepsilon _0 > 0$ such that the following holds for all $0 < \varepsilon \le \varepsilon _0$: there exists a solution $({\pmb {m}}^*, \pmb {J}^*) \in \mathcal{C}\mathcal{E}_\varepsilon ^\mathcal {I}$ satisfying the bounds

8.12a $$\begin{aligned}&\text {(measure approximation)}{} & {} \Vert \iota _\varepsilon ( \widehat{{\pmb {m}}} - {\pmb {m}}^* ) \Vert _{\textrm{KR}( \overline{\mathcal {I}} \times \mathbb {T}^d)} \le \eta ', \end{aligned}$$

8.12b $$\begin{aligned}&\text {(action approximation)}{} & {} \mathcal {A}_\varepsilon ^\mathcal {I}({\pmb {m}}^*, \pmb {J}^*) \le \mathbb {A}_\textrm{hom}^\mathcal {I}({\varvec{\mu }}, {\varvec{\nu }}) +\eta ' + C \varepsilon , \end{aligned}$$

where $C < \infty$ depends on *M*, *A*, $|\mathcal {I}|$, and $\eta '$, but not on $\varepsilon$.

#### Remark 8.15

It is also true that

$$\begin{aligned} \mathcal {A}_\varepsilon ^\mathcal {I}({\pmb {m}}^*, \pmb {J}^*) \le \mathcal {A}_\varepsilon ^\mathcal {I}(\widehat{{\pmb {m}}}, \widehat{\pmb {J}}) + \eta ' + C \varepsilon , \end{aligned}$$

but this information is not “useful”, as we do not expect to be able to control $\mathcal {A}_\varepsilon ^\mathcal {I}(\widehat{{\pmb {m}}}, \widehat{\pmb {J}})$ in terms of $\mathbb {A}_\textrm{hom}^\mathcal {I}({\varvec{\mu }}, {\varvec{\nu }})$; see also Remark 8.12.

The proof consists of four steps: the first one is to consider optimal microstructures associated with $({\varvec{\mu }},{\varvec{\nu }})$ on every scale $\varepsilon >0$ and glue them together to obtain a discrete curve $({\pmb {m}}^*, \pmb {J}^*)$ (we omit the $\varepsilon$-dependence for simplicity). The second step is the space-time regularisation of such measures in the same spirit as done in the proof of Proposition 7.1. Subsequently, we aim at finding suitable correctors in order to obtain a solution to the continuity equation and thus a discrete competitor (in the definition of $\mathcal {A}_\varepsilon$). Finally, the energy estimates conclude the proof of Proposition 8.14.

Let us first discuss the third step, i.e. how to find small correctors for $({\pmb {m}}^*, \pmb {J}^*)$ in order to obtain discrete solutions to $\mathcal{C}\mathcal{E}_\varepsilon ^\mathcal {I}$ which are close to the first ones. Suppose for a moment that $({\pmb {m}}^*, \pmb {J}^*)$ are "regular", as in the outcome of Proposition 7.1. Then the idea is to consider how far they are from solving the continuity equation, i.e. to study the error in the continuity equation

$$\begin{aligned} g_t(x) := \partial _t m_t^*(x) + {{\,\mathrm{\text {\textsf{div}}}\,}}J_t^*(x), \quad x \in \mathcal {X}_\varepsilon , \end{aligned}$$

and find suitable (small) correctors $\varvec{\widetilde{J}}$ to $\pmb {J}^*$ in such a way that $({\pmb {m}}^*, \pmb {J}^* + \widetilde{\pmb {J}}) \in \mathcal{C}\mathcal{E}_\varepsilon ^\mathcal {I}$.

This is based on the next result, which is obtained on the same spirit of Lemma 7.3 in a non-periodic setting. In this case, we are able to ensure good $\ell ^\infty$-bounds and support properties.

#### Lemma 8.16

(Bounds for the divergence equation) Let $g: \mathcal {X}_\varepsilon \rightarrow \mathbb {R}$ with $\sum _{x \in \mathcal {X}_\varepsilon } g(x) = 0$. There exists a vector field $J: \mathcal {E}_\varepsilon \rightarrow \mathbb {R}$ such that

8.13 $$\begin{aligned} {{\,\mathrm{\text {\textsf{div}}}\,}}J = g \quad \text { and }\quad \Vert J \Vert _{\ell ^\infty (\mathcal {E}_\varepsilon )} \le \tfrac{1}{2} \Vert g \Vert _{\ell ^1(\mathcal {X}_\varepsilon )}. \end{aligned}$$

Moreover, ${{\,\textrm{supp}\,}}V \subseteq \textrm{conv}{{\,\textrm{supp}\,}}g+ B_{C\varepsilon }$ with *C* depending only on $\mathcal {X}$.

#### Proof

Let $g_+$ be the positive part of *g*, and let $g_-$ be the negative part. By assumption, these functions have the same $\ell ^1$-norm $N:= \Vert g_-\Vert _{\ell ^1(\mathcal {X}_\varepsilon )} = \Vert g_+\Vert _{\ell ^1(\mathcal {X}_\varepsilon )}$. Let $\Gamma$ be an arbitrary coupling between the discrete probability measures $g_-/N$ and $g_+/N$.

For any $x, y \in {{\,\textrm{supp}\,}}g$: take an arbitrary path $P_{xy}$ connecting these two points. Let $J_xy$ be the unit flux field constructed in Definition 4.4. Then the vector field $J:= \sum _{x, y} \Gamma (x,y) J_{xy}$ has the desired properties.$\square$

#### Remark 8.17

(Measurability) It is clear from the previous proof that one can choose the vector field $J:\mathcal {E}_\varepsilon \rightarrow \mathbb {R}$ in such a way that the function $g \mapsto J$ is a measurable map.

The plan is to apply Lemma 8.16 to a suitable localisation of $g_t$, in each cube $Q_\varepsilon ^z$, for every $z \in \mathbb {Z}_\varepsilon ^d$. Precisely, the goal is to find $g_t(z; \cdot )$ for every $z\in \mathbb {Z}_\varepsilon ^d$ such that

8.14 $$\begin{aligned} \sum _{z\in \mathbb {Z}^d_\varepsilon }g_t(z;x)=g_t(x), \quad \sum _{x\in \mathcal {X}_\varepsilon } g_t(z; x) =0, \end{aligned}$$

which is small on the right scale, meaning

8.15 $$\begin{aligned} {{\,\textrm{supp}\,}}g_t(z;\cdot ) \subset B_\infty (z,R \varepsilon ), \quad \Vert g_t(z;\cdot )\Vert _{\infty }\le C\varepsilon ^d \, . \end{aligned}$$

#### Remark 8.18

Note that $\sum _{x \in \mathcal {X}_\varepsilon } g_t(x) = 0$ for all $t\in \mathcal {I}$, since ${\pmb {m}}^*$ has constant mass in time and $\pmb {J}^*$ is skew-symmetric. However, an application of Lemma 8.16 without localisation would not ensure a uniform bound on the corrector field, as we are not able to control the $\ell ^1$-norm of $g_t$ *a priori*.

#### Remark 8.19

A seemingly natural attempt would be to define $g_t(z;x):= g_t(x) \mathbb {1}_{\{ z \}}(x_\textsf{z})$. However, this choice is not of zero-mass, due to the flow of mass across the boundary of the cubes.

Recall that we use the notation $(\pmb {r}, \pmb {u}) \in \textsf{CE}_{\varepsilon ,d}^\mathcal {I}$ to denote solutions to the continuity equation on $\mathbb {Z}_\varepsilon ^d$ in the sense of Definition 8.2. We shall later apply Lemma 8.22 to the pair $(\pmb {r}, \pmb {u}) = (\textrm{P}_\varepsilon {\varvec{\mu }}, \textrm{P}_\varepsilon {\varvec{\nu }}) \in \textsf{CE}_{\varepsilon ,d}^\mathcal {I}$, thanks to Lemma 8.3.

The notion of *shortest path* in the next definition refers to the $\ell _1$-distance on $\mathbb {Z}_\varepsilon ^d$.

#### Definition 8.20

For all $z',z''\in \mathbb {Z}_\varepsilon ^d$, we choose simultaneously a shortest path $p(z',z''):= (z_0,\ldots ,z_N)$ of nearest neighbors in $\mathbb {Z}_\varepsilon ^d$ connecting $z_0 = z'$ to $z_N = z''$ such that $p(z' + \widetilde{z}, z'' + \widetilde{z}) = p(z',z'') + \widetilde{z}$ for all $\widetilde{z}\in \mathbb {Z}_\varepsilon ^d$. Then define for $z,z',z''\in \mathbb {Z}_\varepsilon ^d$ and $i=1,\ldots ,d$ the signs $\sigma _i^{z;z',z''}\in \{-1,0,1\}$ as

$$\begin{aligned} \sigma _i^{z;z',z''} := {\left\{ \begin{array}{ll} -1 &{} \text {if } (z_{k-1},z_k) = (z,z-e_i)\text { for some }k\text { within }p(z',z''),\\ 1 &{} \text {if } (z_{k-1},z_k) = (z-e_i,z)\text { for some }k\text { within }p(z',z''),\\ 0 &{} \text {otherwise.} \end{array}\right. } \end{aligned}$$

Note that since the paths $p(z',z'')$ are simple, each pair of nearest neighbours appears at most once in any order, so that $\sigma _i^{z; z', z''}$ is well-defined.

It follows readily from Definition 8.20 that

8.16 $$\begin{aligned} \sum _{z\in \mathbb {Z}_\varepsilon ^d} \sigma _i^{z; z', z''} = (z'' - z') \cdot e_i \end{aligned}$$

for all $z', z''\in \mathbb {Z}_\varepsilon ^d$ and $i = 1,\ldots ,d$.

#### Remark 8.21

A canonical choice of the paths $p(z',z'')$ is to interpolate first between $z'_1\in \mathbb {Z}_\varepsilon ^1$ and $z''_1\in \mathbb {Z}_\varepsilon ^1$ one step at a time, then between $z'_2$ and $z''_2$, and so on. The precise choice of path is irrelevant to our analysis as long as paths are short and satisfy $p(z' + \widetilde{z}, z'' + \widetilde{z}) = p(z',z'') + \widetilde{z}$. Since the paths are invariant under translations, so are the signs, i.e.

8.17 $$\begin{aligned} \sigma _i^{z;z' + \widetilde{z}, z'' + \widetilde{z}} = \sigma _i^{z-\widetilde{z}; z', z''} \end{aligned}$$

for all $z,\widetilde{z}, z',z''\in \mathbb {Z}_\varepsilon ^d$, which is used in the prof of Lemma 8.22 below.

Lemma 8.22 shows that if we start from a solution to the continuity equation $({\varvec{\mu }},{\varvec{\nu }}) \in \mathbb{C}\mathbb{E}^\mathcal {I}$ and consider an admissible microstructure $({\pmb {m}}, \pmb {J})=(m_t^z, J_t^z)_{t \in \mathcal {I}, z \in \mathbb {Z}_\varepsilon ^d}$ associated to $(\textrm{P}_\varepsilon {\varvec{\mu }}, \textrm{P}_\varepsilon {\varvec{\nu }})$, then it is possible to localise the error in the continuity equation arising from the gluing $(\mathcal {G}_\varepsilon \pmb {M}, \mathcal {G}_\varepsilon \pmb {U})$ as in (8.14).

#### Lemma 8.22

(Localisation of the error to $\mathcal{C}\mathcal{E}_\varepsilon ^\mathcal {I}$) Let $(\pmb {r}, \pmb {u}) \in \textsf{CE}_{\varepsilon ,d}^\mathcal {I}$ and suppose that $m_t:= (m_t^z)_{z \in \mathbb {Z}_\varepsilon ^d} \subseteq \mathbb {R}_+^\mathcal {X}$ and $J_t:= (J_t^z)_{z \in \mathbb {Z}_\varepsilon ^d} \subseteq \mathbb {R}_a^\mathcal {E}$ satisfy

$$\begin{aligned} ( m_t^z , J_t^z ) \in {{\,\mathrm{{\textsf{Rep}}}\,}}\big ( r_t(z), u_t(z) \big ) \end{aligned}$$

for every $t \in \mathcal {I}$ and $z \in \mathbb {Z}_\varepsilon ^d$. Consider their gluings $\widehat{m}_t:= \mathcal {G}_\varepsilon m_t$ and $\widehat{J}_t:= \mathcal {G}_\varepsilon J_t$ and define, for $z \in \mathbb {Z}_\varepsilon ^d$ and $x \in \mathcal {X}_\varepsilon$,

8.18 $$\begin{aligned} g_t(x)&:= \partial _t \widehat{m}_t(x) + {{\,\mathrm{\text {\textsf{div}}}\,}}\widehat{J}_t(x), \end{aligned}$$

8.19 $$\begin{aligned} g_t(z; x)&:= \partial _t \widehat{m}_t(x) {\textbf{1}}_{\{ z \}}(x_\textsf{z}) + \frac{1}{2} \sum _{y\sim x} \sum _{i=1}^d \sigma _i^{z; x_\textsf{z}, y_\textsf{z}} \Big ( \widetilde{J}_t(z; x, y) - \widetilde{J}_t(z - e_i; x, y) \Big ), \end{aligned}$$

where $\widetilde{J}_t(z; \cdot ): \mathcal {E}_\varepsilon \rightarrow \mathbb {R}$ is the $\mathbb {T}_\varepsilon ^d$-periodic map satisfying $\widetilde{J}_t\big (z; T_\varepsilon ^0(x'), T_\varepsilon ^0(y')\big ) = J_t^z(x', y')$ for all $(x', y') \in \mathcal {E}$. Then the following statements hold for every $t \in \mathcal {I}$:

(i) $g_t(z;x)$ is a localisation of the error $g_t(x)$ of $(\widehat{m}, \widehat{J})$ from solving $\mathcal{C}\mathcal{E}_\varepsilon ^\mathcal {I}$, i.e.,
  $$\begin{aligned} \sum _{z\in \mathbb {Z}^d_\varepsilon } g_t(z;x) = g_t(x) \quad \text {for all } x \in \mathcal {X}_\varepsilon . \end{aligned}$$
(ii) Each localised error $g_t(z;\cdot )$ has zero mass, i.e.,
  $$\begin{aligned} \sum _{x \in \mathcal {X}_\varepsilon } g_t(z; x) = 0 \quad \text {for all } z \in \mathbb {Z}_\varepsilon ^d. \end{aligned}$$

#### Proof

(*i*): For $(x,y) \in \mathcal {E}_\varepsilon$, consider the path $p(x_\textsf{z}, y_\textsf{z}) = (z_0, \ldots , z_N)$ constructed in Definition 8.20. For all $t \in \mathcal {I}$ we have

$$\begin{aligned}&\sum _{z \in \mathbb {Z}^d_\varepsilon } \sum _{i=1}^d \sigma _i^{z; x_\textsf{z}, y_\textsf{z}} \Big ( \widetilde{J}_t(z;x,y) - \widetilde{J}_t(z- e_i;x,y) \Big ) \\&= \sum _{k=1}^{N} \Big ( \widetilde{J}_t(z_k; x,y) - \widetilde{J}_t(z_{k-1}; x,y) \Big ) = \widetilde{J}_t(y_\textsf{z}; x,y) - \widetilde{J}_t(x_\textsf{z}; x,y) . \end{aligned}$$

Summation over all neighbours of $x\in \mathcal {X}_\varepsilon$ yields, for all $t\in \mathcal {I}$,

$$\begin{aligned} \sum _{z\in \mathbb {Z}^d_\varepsilon }g_t(z;x)&= \partial _t m_t(x) + \frac{1}{2} \sum _{y\sim x} \sum _{z \in \mathbb {Z}^d_\varepsilon } \sum _{i = 1}^d \sigma _i^{z; x_\textsf{z}, y_\textsf{z}} \Big ( \widetilde{J}_t(z; x, y) - \widetilde{J}_t(z-e_i; x, y) \Big ) \\&= \partial _t m_t(x) + \frac{1}{2} \sum _{y\sim x} \Big ( \widetilde{J}_t(y_\textsf{z};x, y) - \widetilde{J}_t(x_\textsf{z};x, y) \Big ) \\&= \partial _t m_t(x) + \frac{1}{2} \sum _{y\sim x} \Big ( \widetilde{J}_t(y_\textsf{z}; x, y) + \widetilde{J}_t(x_\textsf{z}; x, y) \Big ) = g_t(x), \end{aligned}$$

where we used the $\mathbb {Z}^d$-periodicity of $(\mathcal {X}, \mathcal {E})$ and the vanishing divergence of $J_t^{x_\textsf{z}}$.

(*ii*): Fix $z \in \mathbb {Z}_\varepsilon ^d$ and $t \in \mathcal {I}$. Using the periodicity of $\widetilde{J}_t(z; \cdot )$, the identity (8.17), the group structure of $\mathbb {Z}_\varepsilon ^d$, the relation between $\widetilde{J}$ and *J*, the fact that $J_t^z \in {{\,\mathrm{{\textsf{Rep}}}\,}}\big ( u_t(z) \big )$, and the identity (8.16), we obtain

$$\begin{aligned}&\sum _{(x,y) \in \mathcal {E}_\varepsilon } \sum _{i=1}^d \sigma _i^{z;x_\textsf{z},y_\textsf{z}} \left( \widetilde{J}_t(z; x,y) - \widetilde{J}_t(z-e_i; x,y) \right) \\&\quad = \sum _{\begin{array}{c} (x,y) \in \mathcal {E}_\varepsilon \\ x_\textsf{z}= z \end{array}} \sum _{\widetilde{z}\in \mathbb {Z}_\varepsilon ^d} \sum _{i=1}^d \sigma _i^{z;x_\textsf{z}+ \widetilde{z},y_\textsf{z}+ \widetilde{z}} \left( \widetilde{J}_t(z; x,y) - \widetilde{J}_t(z-e_i; x,y) \right) \\&\quad = \sum _{\begin{array}{c} (x,y)\in \mathcal {E}_\varepsilon \\ x_\textsf{z}= z \end{array}} \sum _{\widetilde{z}\in \mathbb {Z}_\varepsilon ^d} \sum _{i=1}^d \sigma _i^{z - \widetilde{z};x_\textsf{z},y_\textsf{z}} \left( \widetilde{J}_t(z; x,y) - \widetilde{J}_t(z-e_i; x,y) \right) \\&\quad = \sum _{\begin{array}{c} (x,y) \in \mathcal {E}_\varepsilon \\ x_\textsf{z}= z \end{array}} \sum _{i=1}^d \left( \widetilde{J}_t(z; x,y) - \widetilde{J}_t(z-e_i; x,y) \right) \left( \sum _{\widetilde{z}\in \mathbb {Z}_\varepsilon ^d} \sigma _i^{\widetilde{z};x_\textsf{z},y_\textsf{z}} \right) \\&\quad = \sum _{(x',y') \in \mathcal {E}^Q} \sum _{i=1}^d \Big ( J_t^z(x',y') - J_t^{z-e_i}(x',y') \Big ) (y'_\textsf{z}-x'_\textsf{z}) \cdot e_i \\&\quad = 2 \sum _{i=1}^d \big (u_t(z) - u_t(z-e_i)\big ) \cdot e_i. \end{aligned}$$

By definition of $g_t(z;\cdot )$ we obtain

$$\begin{aligned} \sum _{x\in \mathcal {X}_\varepsilon } g_t(z;x)&= \sum _{\begin{array}{c} x\in \mathcal {X}_\varepsilon \\ x _\textsf{z}= z \end{array}} \partial _t m_t(x) + \frac{1}{2} \sum _{i=1}^d \sum _{(x,y)\in \mathcal {E}_\varepsilon } \sigma _i^{z;x_\textsf{z},y_\textsf{z}} \Big (\widetilde{J}_t(z; x,y) - \widetilde{J}_t(z-e_i;x,y) \Big ) \\&= \partial _t r_t(z) + \sum _{i=1}^d \big (u_t(z)-u_t(z- e_i)\big )\cdot e_i = 0, \end{aligned}$$

where we used that $m_t^z \in {{\,\mathrm{{\textsf{Rep}}}\,}}\big (r_t(z)\big )$ and eventually that $(\pmb {r}, \pmb {u}) \in \textsf{CE}_{\varepsilon ,d}^\mathcal {I}$.

Now we are ready to prove Proposition 8.14.

#### Proof of Proposition 8.14

The proof consists of four steps. For simplicity: $\mathcal {I}:=\mathcal {I}_\eta$.

*Step 1: Regularisation*. Recall the operators $R_\delta$, $S_\lambda$, and $T_\tau$ as defined in Sect. 7.1. We define

$$\begin{aligned} {\pmb {m}}^* := \Big ( R_\delta \circ S_\lambda \circ T_\tau \Big ) \widehat{{\pmb {m}}} \quad \text { and }\quad \bar{\pmb {J}}^* := \Big ( R_\delta \circ S_\lambda \circ T_\tau \Big ) \widehat{\pmb {J}}, \end{aligned}$$

where $\delta , \lambda >0$, $0<\tau <\eta$ will be chosen sufficiently small, depending on the desired accuracy $\eta ' >0$. Due to special linear structure of the gluing operator $\mathcal {G}_\varepsilon$, it is clear that

$$\begin{aligned} {\pmb {m}}^* = \mathcal {G}_\varepsilon \overline{{\pmb {m}}}\quad \text { and }\quad \quad \bar{\pmb {J}}^* = \mathcal {G}_\varepsilon \bar{\pmb {J}}, \end{aligned}$$

for some $\big ( \overline{{\pmb {m}}}, \bar{\pmb {J}} \big ) = (\overline{m}_t^z, \bar{J}_t^z)_{t \in \mathcal {I}, z \in \mathbb {Z}_\varepsilon ^d}$. More precisely, they correspond to the regularised version of the measures $(m_t^z, J_t^z)_{t \in \mathcal {I}, z \in \mathbb {Z}_\varepsilon ^d}$ with respect to the graph structure of $\mathbb {Z}_\varepsilon ^d$. In particular, an application^4^ of Lemma 8.13, Lemma 7.6, and Lemma 7.7 yields

8.20 $$\begin{aligned} \begin{gathered} \sup _{t \in \mathcal {I}} \big \Vert \overline{m}_t^{\cdot + z} - \overline{m}_t \big \Vert _{\ell ^\infty (\mathbb {Z}_\varepsilon ^d \times \mathcal {X})} + \varepsilon \big \Vert \bar{J}_t^{\cdot + z} - \bar{J}_t \big \Vert _{\ell ^\infty (\mathbb {Z}_\varepsilon ^d \times \mathcal {E})} \le C |z| \varepsilon ^{d+1}, \\ \sup _{t \in \mathcal {I}} \big \Vert \partial _t \overline{m}_t \big \Vert _{\ell ^\infty (\mathbb {Z}_\varepsilon ^d \times \mathcal {X})} \le C \varepsilon ^d, \end{gathered} \end{aligned}$$

for any $z \in \mathbb {Z}_\varepsilon ^d$, as well as the domain regularity

8.21 $$\begin{aligned} \bigg \{ \bigg ( \frac{\overline{m}_t^z}{\varepsilon ^d}, \frac{\bar{J}_t^z}{\varepsilon ^{d-1}} \bigg ) \ : \ z \in \mathbb {Z}_\varepsilon ^d, \, t \in \mathcal {I}\bigg \} \subset K \Subset ({{\,\mathrm{\textsf{D}}\,}}F)^\circ , \end{aligned}$$

for a constant *C* and a compact set *K* depending only on *M*, *A*, $\delta$, $\lambda$, and $\tau$. We can then apply Lemma 8.7 and deduce that for every $t \in \mathcal {I}$, $\varepsilon \le \varepsilon _0$ (depending on *K* and *F*),

8.22 $$\begin{aligned} \mathcal {F}_\varepsilon \big ( m_t^*, \bar{J}_t^* \big ) \le \sum _{z \in \mathbb {Z}_\varepsilon ^d} \varepsilon ^d F \bigg ( \frac{\overline{m}_t^z}{\varepsilon ^d} , \frac{\bar{J}_t^z}{\varepsilon ^{d-1}} \bigg ) + c \varepsilon , \end{aligned}$$

for a $c \in \mathbb {R}^+$ depending on the same set of parameters (via *C* and $\textrm{Lip}(F;K)$) and $R_1$.

*Step 2: Construction of a solution to* $\mathcal{C}\mathcal{E}_\varepsilon ^\mathcal {I}$. From now on, the constant *C* appearing in the estimates might change line by line, but it always depends on the same set of parameters as the constant *C* in Step 1, and possibly on the size of the time interval $|\mathcal {I}|$.

The next step is to find a quantitative small corrector $\pmb {V}$ in such a way that $({\pmb {m}}^*, \bar{\pmb {J}}^* + \pmb {V}) \in \mathcal{C}\mathcal{E}_\varepsilon ^\mathcal {I}$. To do so, we observe that by construction we have for every $t \in \mathcal {I}$

$$\begin{aligned} \Big ( \overline{m}_t^z , \bar{J}_t^z \Big ) \in {{\,\mathrm{{\textsf{Rep}}}\,}}\Big ( r_t^*(z) , u^*_t(z) \Big ), \end{aligned}$$

where $(\pmb {r}^*, \pmb {u}^*) \in \textsf{CE}_{\varepsilon ,d}^\mathcal {I}$ (by the linearity of equation (8.1)). Consider the corresponding error functions, for $(x,y) \in \mathcal {E}_\varepsilon$, $t \in \mathcal {I}$, $z \in \mathbb {Z}_\varepsilon ^d$ given by (8.18) and (8.19),

$$\begin{aligned} g_t(x)&:= \partial _t m_t^*(x) + {{\,\mathrm{\text {\textsf{div}}}\,}}\bar{J}_t^*(x), \\ g_t(z;x)&:=\partial _t m_t^*(x) \mathbb {1}_{\{ x_\textsf{z}= z \}}(x) + \frac{1}{2} \sum _{y\sim x}\sum _{i=1}^d \sigma _i^{z;x_\textsf{z},y_\textsf{z}}(\widetilde{J}(z;x,y)-\widetilde{J}(z-e_i;x,y)), \end{aligned}$$

where $\widetilde{J}(z;\cdot ):\mathcal {E}_\varepsilon \rightarrow \mathbb {R}$ is the $\mathbb {T}_\varepsilon ^d$-periodic map satisfying $\widetilde{J}(z;T_\varepsilon ^0(x'),T_\varepsilon ^0(y')) = \bar{J}_t^z(x',y')$, for any $(x',y')\in \mathcal {E}$. Thanks to Lemma 8.22, we know that

$$\begin{aligned} \sum _{x\in \mathcal {X}_\varepsilon } g_t(z; x) = 0, \quad \sum _{z' \in \mathbb {Z}_\varepsilon ^d} g_t(z';x) = g_t(x), \quad \forall x \in \mathcal {X}_\varepsilon , \, z \in \mathbb {Z}_\varepsilon ^d. \end{aligned}$$

Moreover, from the regularity estimates (8.20) and the local finiteness of the graph $(\mathcal {X},\mathcal {E})$, we infer for every $z \in \mathbb {Z}_\varepsilon ^d$

8.23 $$\begin{aligned} \left\| g_t(z;\cdot ) \right\| _{\ell ^\infty (\mathcal {X}_\varepsilon )} \le C \varepsilon ^d, \quad {{\,\textrm{supp}\,}}g_t(z; \cdot ) \subset \{ x \in \mathcal {X}_\varepsilon \ : \ \Vert x_\textsf{z}- z \Vert _{\ell ^\infty (\mathbb {Z}_\varepsilon ^d)} \le C' \}, \end{aligned}$$

where $C'$ only depends on $(\mathcal {X},\mathcal {E})$. Hence, as an application of Lemma 8.16, we deduce the existence of corrector vector fields $V_t \in \mathbb {R}_a^{\mathbb {Z}_\varepsilon ^d \times \mathcal {E}_\varepsilon }$ such that

8.24 $$\begin{aligned} \begin{gathered} {{\,\mathrm{\text {\textsf{div}}}\,}}V_t(z;\cdot ) = g_t(z;\cdot ) \ , \quad {{\,\textrm{supp}\,}}V_t(z;\cdot ) \subset \{ (x,y) \in \mathcal {E}_\varepsilon \ : \ \Vert x_\textsf{z}- z \Vert _{\ell ^\infty (\mathbb {Z}_\varepsilon ^d)} \le \widetilde{C}' \}, \\ \Vert V_t(z;\cdot ) \Vert _{\ell ^\infty (\mathcal {E}_\varepsilon )} \le \tfrac{1}{2} \Vert g_t(z;\cdot ) \Vert _{\ell ^1(\mathcal {X}_\varepsilon )} \le C \varepsilon ^d, \end{gathered} \end{aligned}$$

for every $t \in \mathcal {I}$, $z \in \mathbb {Z}_\varepsilon ^d$. The existence of a measurable (in $t \in \mathcal {I}$ and $z \in \mathbb {Z}_\varepsilon ^d$) map $V_t(z;\cdot )$ follows from the measurability of $g_t(z;\cdot )$ and Remark 8.17.

We then define $\pmb {V}: \mathcal {I}\rightarrow \mathbb {R}_a^{\mathcal {E}_\varepsilon }$ and $\pmb {J}^*: \mathcal {I}\rightarrow \mathbb {R}_a^{\mathcal {E}_\varepsilon }$ as

$$\begin{aligned} \pmb {V}:= \sum _{z \in \mathbb {Z}_\varepsilon ^d}\pmb {V}(z;\cdot ), \quad \pmb {J}^*:= \bar{\pmb {J}}^* + \pmb {V}, \end{aligned}$$

and obtain a solution to the discrete continuity equation $({\pmb {m}}^*, \pmb {J}^*) \in \mathcal{C}\mathcal{E}_\varepsilon ^\mathcal {I}$.

*Step 3: Energy estimates*. The locality property (8.24) of $V_t(z;\cdot )$ and local finiteness of the graph $(\mathcal {X},\mathcal {E})$ allow us to deduce the same uniform estimates on the global corrector as well. Indeed for every $t\in \mathcal {I}$, $x \in \mathcal {X}_\varepsilon$ we have

$$\begin{aligned} V_t(x,y) := \sum _{z \in B_\infty (x_\textsf{z}; \widetilde{C}') } V(z;x,y), \quad B_\infty (x_\textsf{z}; \widetilde{C}') := \left\{ z \in \mathbb {Z}_\varepsilon ^d \ : \ \Vert z - x_\textsf{z}\Vert _{\ell ^\infty (\mathbb {Z}_\varepsilon ^d)} \le \widetilde{C}' \right\} , \end{aligned}$$

and hence from the estimate (8.24) we also deduce $\Vert \pmb {V}\Vert _{\ell ^\infty (\mathcal {I}\times \mathcal {E}_\varepsilon )} \le C \varepsilon ^d$.

Since (8.21) implies that $\displaystyle \bigg (\frac{\tau _\varepsilon ^z m_t^*}{\varepsilon ^d}, \frac{\tau _\varepsilon ^z \bar{J}_t^*}{\varepsilon ^{d-1}} \bigg ) \in K$, it then follows that $\displaystyle \bigg (\frac{\tau _\varepsilon ^z m_t^*}{\varepsilon ^d}, \frac{\tau _\varepsilon ^z J_t^*}{\varepsilon ^{d-1}} \bigg ) \in K'$ for $0<\varepsilon \le \varepsilon _0$ sufficiently small, where $\varepsilon _0$ depends on *K* and *C*. Here $K'$ is a compact set, possibly slightly larger than *K*, contained in ${{\,\mathrm{\textsf{D}}\,}}(F)^\circ$.

Therefore, we can estimate the energy

$$\begin{aligned} \sup _{t \in \mathcal {I}} \sup _{z \in \mathbb {Z}_\varepsilon ^d} \left| F \bigg (\frac{\tau _\varepsilon ^z m_t^*}{\varepsilon ^d} , \frac{\tau _\varepsilon ^z \bar{J}_t^*}{\varepsilon ^{d-1}} \bigg ) - F \bigg (\frac{\tau _\varepsilon ^z m_t^*}{\varepsilon ^d} , \frac{\tau _\varepsilon ^z J_t^*}{\varepsilon ^{d-1}} \bigg ) \right| \le \textrm{Lip}(F;K') \frac{1}{\varepsilon ^{d-1}} \Vert \pmb {V}\Vert _{\ell ^\infty (\mathcal {I}\times \mathcal {E}_\varepsilon )} \le C \varepsilon , \end{aligned}$$

and hence $\mathcal {A}_\varepsilon ^\mathcal {I}\big ( {\pmb {m}}^*, \pmb {J}^* \big ) \le \mathcal {A}_\varepsilon ^\mathcal {I}\big ( {\pmb {m}}^*, \bar{\pmb {J}}^* \big ) + C \varepsilon$. Together with (8.22), this yields

$$\begin{aligned} \mathcal {A}_\varepsilon ^\mathcal {I}\big ( {\pmb {m}}^*, \pmb {J}^* \big ) \le \int _\mathcal {I}\sum _{z \in \mathbb {Z}_\varepsilon ^d} \varepsilon ^d F \bigg ( \frac{\overline{m}_t^z}{\varepsilon ^d} , \frac{\bar{J}_t^z}{\varepsilon ^{d-1}} \bigg ) \, \textrm{d}t + C \varepsilon . \end{aligned}$$

Finally, to control the action of the regularised microstructures $(\bar{{\pmb {m}}}, \bar{\pmb {J}})$, we take advantage (as in (7.16)) of Lemma 7.5, Lemma 7.6 (*i*), and Lemma 7.7 (*i*) to obtain^5^for a $c' < \infty$, where at last we used Proposition 8.11 and that $f_\textrm{hom}$ is Lipschitz on $\widetilde{K}$.

For every given $\eta '>0$, the action bound (8.12b) then follows choosing $\tau ,\delta >0$ small enough.

*Step 4: Measures comparison.* We have seen in (7.15) that Lemma 7.8 implies

$$\begin{aligned} \Vert \iota _\varepsilon {\pmb {m}}^* - \iota _\varepsilon \widehat{{\pmb {m}}} \Vert _{\textrm{KR}([0,T] \times \mathbb {T}^d)} \lesssim M( \tau + \sqrt{\lambda } + \delta ) + m^\circ (\mathcal {X}^Q) \delta , \end{aligned}$$

where we also used that mass preservation of the gluing operator, see Remark 8.6. For every $\eta '>0$, the distance bound (8.12a) can be then obtained choosing $\tau$, $\lambda$, $\delta$ sufficiently small.

### Proof of the upper bound

This subsection is devoted to the proof of the limsup inequality in Theorem 5.1. First we formulate the existence of a recovery sequence in the smooth case.

#### Proposition 8.23

(Existence of a recovery sequence, smooth case) Fix $\mathcal {I}= (a,b)$, $a<b$, $\eta >0$, and set $\mathcal {I}_\eta := (a-\eta , b+\eta )$. Let $({\varvec{\mu }}, {\varvec{\nu }}) \in \mathbb{C}\mathbb{E}^{\mathcal {I}_\eta }$ be a solution to the continuity equation with smooth densities $(\rho _t, j_t)_{t\in \mathcal {I}_\eta }$ and such that

8.25 $$\begin{aligned} \mathbb {A}_\textrm{hom}^{\mathcal {I}_\eta }({\varvec{\mu }}, {\varvec{\nu }}) < \infty \quad \text { and }\quad \Big \{ \big (\rho _t(x),j_t(x)\big ) \ : \ (t,x) \in \mathcal {I}_\eta \times \mathbb {T}^d\Big \} \Subset {{\,\mathrm{\textsf{D}}\,}}(f_{\textrm{hom}})^\circ . \end{aligned}$$

Then there exists a sequence of curves $(m_t^\varepsilon )_{t \in \overline{\mathcal {I}}} \subseteq \mathbb {R}_+^{\mathcal {X}_\varepsilon }$ such that $\iota _\varepsilon {\pmb {m}}^\varepsilon \rightarrow {\varvec{\mu }}|_{\overline{\mathcal {I}}\times \mathbb {T}^d}$ weakly in $\mathcal {M}_+(\overline{\mathcal {I}}\times \mathbb {T}^d)$ as $\varepsilon \rightarrow 0$ and

8.26 $$\begin{aligned} \limsup _{\varepsilon \rightarrow 0} \mathcal {A}_\varepsilon ^\mathcal {I}({\pmb {m}}^\varepsilon ) \le \mathbb {A}_\textrm{hom}^{\mathcal {I}_\eta }({\varvec{\mu }}, {\varvec{\nu }}) + C \eta |\mathcal {I}| \big (\mu _0(\mathbb {T}^d) + 1\big ), \end{aligned}$$

for some $$C < \infty $$.

#### Proof

We write $\textrm{KR}_\mathcal {I}:= \textrm{KR}(\overline{\mathcal {I}} \times \mathbb {T}^d)$. Let $({\varvec{\mu }},{\varvec{\nu }})\in \mathbb{C}\mathbb{E}^{\mathcal {I}_\eta }$ be smooth curves of measures satisfying the assumptions (8.25). Let $(\widehat{{\pmb {m}}}, \widehat{\pmb {J}})$ be the gluing of a measurable family of optimal microstructure associated with $({\varvec{\mu }}, {\varvec{\nu }})$, for every $\varepsilon >0$. For every $\eta '>0$, Proposition 8.14 yields the existence of $({\pmb {m}}^{\eta '}, \pmb {J}^{\eta '}) \in \mathcal{C}\mathcal{E}_\varepsilon ^\mathcal {I}$, a constant $C_{\eta '}$, and $\varepsilon _0=\varepsilon _0(\eta ')$ depending on $\eta '$ such that

$$\begin{aligned} \Vert \iota _\varepsilon ({\pmb {m}}^{\eta '} - \widehat{{\pmb {m}}} ) \Vert _{\textrm{KR}_\mathcal {I}} \le \eta ', \quad \mathcal {A}_\varepsilon ({\pmb {m}}^{\eta '}, \pmb {J}^{\eta '}) \le \mathbb {A}_{\textrm{hom}}({\varvec{\mu }}, {\varvec{\nu }}) + \eta ' + \varepsilon C_{\eta '}, \end{aligned}$$

for every $$\varepsilon \le \varepsilon _0$$.

Using Remark 8.6, in particular (8.3), and that $({\pmb {m}}^{\eta '}, \pmb {J}^{\eta '}) \in \mathcal{C}\mathcal{E}_\varepsilon ^\mathcal {I}$, we infer

$$\begin{aligned} \Vert \iota _\varepsilon ({\pmb {m}}^{\eta '}) - {\varvec{\mu }}\Vert _{\textrm{KR}_\mathcal {I}} \le \eta ' + {\varvec{\mu }}(\overline{\mathcal {I}} \times \mathbb {T}^d) \varepsilon ^d, \quad \mathcal {A}_\varepsilon ({\pmb {m}}^{\eta '}) \le \mathbb {A}_{\textrm{hom}}({\varvec{\mu }}, {\varvec{\nu }}) + \eta ' + \varepsilon C_{\eta '}. \end{aligned}$$

for every $\varepsilon \le \varepsilon _0$. Therefore, we can find a diagonal sequence $\eta '=\eta '(\varepsilon ) \rightarrow 0$ as $\varepsilon \rightarrow 0$ such that, if we set ${\pmb {m}}^\varepsilon := {\pmb {m}}^{\eta '(\varepsilon )}$, we obtain

$$\begin{aligned} \lim _{\varepsilon \rightarrow 0} \Vert \iota _\varepsilon ({\pmb {m}}^\varepsilon ) - {\varvec{\mu }}\Vert _{\textrm{KR}_\mathcal {I}} = 0, \\ \limsup _{\varepsilon \rightarrow 0} \mathcal {A}_\varepsilon ^\mathcal {I}({\pmb {m}}^\varepsilon ) \le \mathbb {A}_{\textrm{hom}}^\mathcal {I}({\varvec{\mu }}, {\varvec{\nu }}) \le \mathbb {A}_{\textrm{hom}}^{\mathcal {I}_\eta }({\varvec{\mu }}, {\varvec{\nu }}) + C \eta |\mathcal {I}| (\mu _0(\mathbb {T}^d) +1), \end{aligned}$$

where at last we used the growth condition (3.2).$\square$

In order to apply Proposition 8.23 for the existence of the recovery sequence in Theorem 5.1 we prove that the set of solutions to the continuity equation (3.4) with smooth densities are dense-in-energy for $\mathbb {A}_\textrm{hom}^\mathcal {I}$.

#### Definition 8.24

(Affine change of variable in time) Fix $\mathcal {I}= (a,b)$. For any $\eta >0$, we consider the unique bijective increasing affine map $S^\eta :\mathcal {I}\rightarrow (a-2\eta , b + 2\eta )$. For every interval $\widetilde{\mathcal {I}} \subseteq \mathcal {I}$ and every vector-valued measure ${\varvec{\xi }}\in \mathcal {M}^n(\widetilde{\mathcal {I}} \times \mathbb {T}^d)$, $n \in \mathbb {N}$, we define the changed-variable measure

8.27 $$\begin{aligned} \begin{gathered} S ^\eta [{\varvec{\xi }}] \in \mathcal {M}^n(S^\eta (\widetilde{\mathcal {I}}) \times \mathbb {T}^d), \quad S ^\eta [{\varvec{\xi }}]:= \frac{|\mathcal {I}|+4\eta }{|\mathcal {I}|}\big ( S^\eta , {{\,\textrm{id}\,}}\big )_{\#} {\varvec{\xi }}. \end{gathered} \end{aligned}$$

#### Remark 8.25

(Properties of $\text{ S}^\eta$) The scaling factor of $S^\eta [{\varvec{\xi }}]$ is chosen so that if ${\varvec{\xi }}\ll \mathscr {L}^{d+1}$, then $\text{ S}^\eta [{\varvec{\xi }}] \ll \mathscr {L}^{d+1}$ and we have for $(t,x) \in S^\eta (\widetilde{\mathcal {I}}) \times \mathbb {T}^d$ the equality of densities

8.28 $$\begin{aligned} \frac{ \, \textrm{d}\text{ S}^\eta [{\varvec{\xi }}] }{ \, \textrm{d}\mathscr {L}^{d+1} }(t,x) = \frac{ \, \textrm{d}{\varvec{\xi }}}{ \, \textrm{d}\mathscr {L}^{d+1} }((S^\eta )^{-1}(t),x). \end{aligned}$$

Moreover, if $({\varvec{\mu }},{\varvec{\nu }}) \in \mathbb{C}\mathbb{E}^\mathcal {I}$ then $\big ( \frac{|\mathcal {I}|+4\eta }{|\mathcal {I}|} \text{ S}^\eta [{\varvec{\mu }}], \text{ S}^\eta [{\varvec{\nu }}] \big ) \in \mathbb{C}\mathbb{E}^{S^\eta (\mathcal {I})}$.

We are ready to state and prove the last result of this section.

#### Proposition 8.26

(Smooth approximation of finite action solutions to $\mathbb{C}\mathbb{E}^\mathcal {I}$) Fix $\mathcal {I}:= (a,b)$ and fix $({\varvec{\mu }},{\varvec{\nu }})$ $\in \mathbb{C}\mathbb{E}^\mathcal {I}$ with $\mathbb {A}_\textrm{hom}({\varvec{\mu }},{\varvec{\nu }})<\infty$. Then there exists a sequence $\{ \eta _k\}_k \subset \mathbb {R}^+$ such that $\eta _k \rightarrow 0$ as $k \rightarrow \infty$ and measures $({\varvec{\mu }}^k,{\varvec{\nu }}^k) \in \mathbb{C}\mathbb{E}^{\mathcal {I}_k}$ for $\mathcal {I}_k:= (a-\eta _k, b+ \eta _k)$ so that as $k \rightarrow \infty$

8.29 $$\begin{aligned}{} & {} ({\varvec{\mu }}^k,{\varvec{\nu }}^k) \rightarrow ({\varvec{\mu }}, {\varvec{\nu }}) \; \text {weakly in } \mathcal {M}_+\big (\mathcal {I}\times \mathbb {T}^d\big ) \times \mathcal {M}^d \big (\mathcal {I}\times \mathbb {T}^d\big ), \end{aligned}$$

8.30 $$\begin{aligned}{} & {} \frac{\, \textrm{d}{\varvec{\mu }}^k}{\, \textrm{d}\mathscr {L}^{d+1}} \in \mathcal {C}_b^\infty \big (\mathcal {I}_k\times \mathbb {T}^d\big ), \quad \frac{\, \textrm{d}{\varvec{\nu }}^k}{\, \textrm{d}\mathscr {L}^{d+1}} \in \mathcal {C}_b^\infty \big (\mathcal {I}_k\times \mathbb {T}^d;\mathbb {R}^d\big ), \end{aligned}$$

and such that the following action bound holds true:

8.31 $$\begin{aligned} \limsup _{k \rightarrow \infty } \mathbb {A}_\textrm{hom}^{\mathcal {I}_k}({\varvec{\mu }}^k,{\varvec{\nu }}^k) \le \mathbb {A}_\textrm{hom}^\mathcal {I}({\varvec{\mu }},{\varvec{\nu }}). \end{aligned}$$

Moreover, for any given $k\in \mathbb {N}$ we have the inclusion

8.32 $$\begin{aligned} \Big \{ \Big ( \frac{\, \textrm{d}{\varvec{\mu }}^k}{\, \textrm{d}\mathscr {L}^{d+1}}(t,x),\frac{\, \textrm{d}{\varvec{\nu }}^k}{\, \textrm{d}\mathscr {L}^{d+1}}(t,x) \Big ) : (t,x)\in \mathcal {I}_k\times \mathbb {T}^d \Big \} \Subset ({{\,\mathrm{\textsf{D}}\,}}f_\textrm{hom})^\circ . \end{aligned}$$

#### Proof

Without loss of generality we can assume $f_\textrm{hom}\ge 0$, if not we simply consider $g(\rho ,j) = f_\textrm{hom}(\rho ,j) + C\rho + C$ for $C \in \mathbb {R}_+$ as in Lemma 3.14. For simplicity, we also assume $\mathcal {I}:= (0,T)$, the extension to a generic interval is straightforward.

Fix $$({\varvec{\mu }},{\varvec{\nu }})$$ $$\in \mathbb{C}\mathbb{E}^T$$ with $$\mathbb {A}_\textrm{hom}({\varvec{\mu }}, {\varvec{\nu }})<\infty $$.

*Step 1: regularisation*. The first step is to regularise in time and space. To do so, we consider two sequences of smooth mollifiers $\varphi _1^k: \mathbb {R}\rightarrow \mathbb {R}_+$, $\varphi _2^k: \mathbb {T}^d\rightarrow \mathbb {R}$ for $k \in \mathbb {N}$ of integral 1, where ${{\,\textrm{supp}\,}}\varphi _1^k = [-\alpha _k, \alpha _k]$, ${{\,\textrm{supp}\,}}\varphi _2^k =B_{\frac{1}{k}}(0)\subset \mathbb {T}^d$ with $\alpha _k \rightarrow 0$ as $k \rightarrow \infty$ to be suitably chosen. We then set $\varphi ^k:\mathbb {R}\times \mathbb {T}^d\rightarrow \mathbb {R}_+$ as $\varphi ^k(t,x):= \varphi _1^k(t)\varphi _2^k(x)$.

We define space-time regular solutions to the continuity equation as

$$\begin{aligned} (\widetilde{{\pmb {m}}}u^k, \widetilde{{\varvec{\nu }}}^k )&:= \varphi ^k * ({\varvec{\mu }}, {\varvec{\nu }}) \in \mathbb{C}\mathbb{E}^{(\alpha _k,T-\alpha _k)} , \\ (\widehat{{\pmb {m}}}u^k, \widehat{{\varvec{\nu }}}^k )&:=\Big ( \frac{T+4\eta _k}{T}\text{ S}^{\eta _k}[\widetilde{{\pmb {m}}}u^k], \text{ S}^{\eta _k}[\widetilde{\varvec{\nu }}^k] \Big ) \in \mathbb{C}\mathbb{E}^{\mathcal {I}_k}, \end{aligned}$$

where $\mathcal {I}_k:= S^{\eta _k}\big ((\alpha _k, T-\alpha _k)\big )$. Note that the mollified measures are defined only We choose $\alpha _k:= \frac{T\eta _k}{T+4\eta _k}$, so that $\mathcal {I}_k = (-\eta _k,T+\eta _k)$.

Finally, for $(\rho ^\circ ,j^\circ )$ as given in (4.8), we define

8.33 $$\begin{aligned} \begin{gathered} ({\varvec{\mu }}^k, {\varvec{\nu }}^k):= (1-\delta _k) (\widehat{{\pmb {m}}}u^k, \widehat{{\varvec{\nu }}}^k) + \delta _k (\rho ^\circ , j^\circ ) \mathscr {L}^{d+1} \in \mathbb{C}\mathbb{E}^{\mathcal {I}_k}, \end{gathered} \end{aligned}$$

for some suitable choice of $\eta _k, \delta _k \rightarrow 0$.

*Step 2: Properties of the regularised measures.* First of all, we observe that $({\varvec{\mu }}^k,{\varvec{\nu }}^k) \ll \mathscr {L}^{d+1}$ with smooth densities for every $k \in \mathbb {N}$, so that (8.30) is satisfied. Secondly, the convergence (8.29) easily follows by the properties of the mollifiers and the fact that $S^\eta \rightarrow {{\,\textrm{id}\,}}$ uniformly in (0, *T*) as $\eta \rightarrow 0$.

Moreover, we note that for $t>0$, using that $\mu _t(\mathbb {T}^d)$ is constant on (0, *T*) one gets

8.34 $$\begin{aligned} \begin{aligned} \sup _{t \in (\alpha _k, T-\alpha _k)} \Big \Vert \frac{ \, \textrm{d}\widetilde{\mu }_t^k }{ \, \textrm{d}x } \Big \Vert _\infty&\le \Vert \varphi _2^k \Vert _\infty {\varvec{\mu }}\big ((0,T)\times \mathbb {T}^d\big ) =: C_k< +\infty , \\ \Big \Vert \frac{ \, \textrm{d}\widetilde{{\varvec{\nu }}}^k }{ \, \textrm{d}\mathscr {L}^{d+1} } \Big \Vert _\infty&\le \Vert \varphi ^k \Vert _\infty |{\varvec{\nu }}| \big ((0,T)\times \mathbb {T}^d\big ) < \infty , \end{aligned} \end{aligned}$$

and thanks to (8.28) an analogous uniform estimate holds true for $(\widehat{\varvec{\mu }}^k, \widehat{{\varvec{\nu }}}^k)$ too. We can then apply Lemma C.1 and find convex compact sets $K_k \subset ({{\,\mathrm{\textsf{D}}\,}}f_\textrm{hom})^\circ$ such that $\displaystyle \Big \{\Big ( \frac{\, \textrm{d}{\varvec{\mu }}^k}{\, \textrm{d}\mathscr {L}^{d+1}}(\cdot ), \frac{\, \textrm{d}{\varvec{\nu }}^k}{\, \textrm{d}\mathscr {L}^{d+1}}(\cdot ) \Big )\Big \} \subset K_k$, so that (8.32) follows.

Additionally, pick $\theta >0$ such that $B^\circ :=B((\rho ^\circ , j^\circ ), \theta ) \subset ({{\,\mathrm{\textsf{D}}\,}}f_\textrm{hom})^\circ$. From (8.28), if one sets $S_k:= S^{\eta _k}$, we see that

8.35 $$\begin{aligned} \Big ( \frac{\, \textrm{d}{\varvec{\mu }}^k}{\, \textrm{d}\mathscr {L}^{d+1}},\frac{\, \textrm{d}{\varvec{\nu }}^k}{\, \textrm{d}\mathscr {L}^{d+1}}\Big ) (t,x)=(1-\delta _k) \Big ( \frac{\, \textrm{d}\widetilde{\varvec{\mu }}^{k}}{\, \textrm{d}\mathscr {L}^{d+1}},\frac{\, \textrm{d}\widetilde{\varvec{\mu }}^{k}}{\, \textrm{d}\mathscr {L}^{d+1}}\Big ) (S_k^{-1}(t),x) +\delta _k (\widetilde{\rho }_t^k(x), j^\circ ) \end{aligned}$$

for $t \in \mathcal {I}_k$ and $x \in \mathbb {T}^d$, where the functions $\widetilde{\rho } ^k$ are given by

$$\begin{aligned} \widetilde{\rho }_t^k(x) := \rho ^\circ +\frac{1-\delta _k}{\delta _k} 2\eta _k \frac{\, \textrm{d}\widetilde{\varvec{\mu }}^{k}}{\, \textrm{d}\mathscr {L}^{d+1}}(S_k^{-1}(t),x). \end{aligned}$$

We choose $\delta _k$ such that $\theta \delta _k > 2\eta _k C_k$ and from (8.34) we get that

8.36 $$\begin{aligned} (\widetilde{\rho }_t^k(x),j^\circ ) \in B^\circ , \quad \forall t \in \mathcal {I}_k, \; x \in \mathbb {T}^d, \; k \in \mathbb {N}. \end{aligned}$$

For example we can pick $\eta _k:= (4kC_k)^{-1}$ and $\theta \delta _k = k^{-1}$, both going to zero when $k \rightarrow +\infty$.

*Step 3: action estimation*. As the next step we show that

8.37 $$\begin{aligned} \mathbb {A}_\textrm{hom}^{(\alpha _k,T-\alpha _k)} \big ( \widetilde{\varvec{\mu }}^k, \widetilde{{\varvec{\nu }}}^k \big ) \le \mathbb {A}_\textrm{hom}^T ({\varvec{\mu }}, {\varvec{\nu }}), \quad \forall k \in \mathbb {N}. \end{aligned}$$

One can prove (8.37) using e.g. the fact [10] that for every interval $\mathcal {I}$ the action $\mathbb {A}_\textrm{hom}^\mathcal {I}$ is the relaxation of the functional

$$\begin{aligned} ({\varvec{\mu }}, {\varvec{\nu }}) \mapsto {\left\{ \begin{array}{ll} \displaystyle \int _{\mathcal {I}\times \mathbb {T}^d}f_\textrm{hom}\left( \frac{\, \textrm{d}{\varvec{\mu }}}{\, \textrm{d}\mathscr {L}^{d+1}}, \frac{\, \textrm{d}{\varvec{\nu }}}{\, \textrm{d}\mathscr {L}^{d+1}} \right) \, \textrm{d}\mathscr {L}^{d+1}, &{}\text { if }( {\varvec{\mu }}, {\varvec{\nu }}) \ll \, \textrm{d}\mathscr {L}^{d+1}, \\ +\infty , &{}\text {otherwise}, \end{array}\right. } \end{aligned}$$

for which (8.37) follows from the convexity and nonnegativity of $f_\textrm{hom}$ and the properties of the mollifiers $\varphi ^k$.

We shall then estimate the action of $({\varvec{\mu }}^k, {\varvec{\nu }}^k)$. From (8.35) and (8.36), using the convexity of $f_\textrm{hom}$ and the definition of the map $S^\eta$, we obtain

$$\begin{aligned} \mathbb {A}_\textrm{hom}^{\mathcal {I}_k}({\varvec{\mu }}^k,{\varvec{\nu }}^k)&- (1+2\eta _k) \delta _k \sup _{B^\circ } f_\textrm{hom}\\&\le (1-\delta _k) \int \limits _{\mathcal {I}_k \times \mathbb {T}^d} f_\textrm{hom}\Big (\frac{\, \textrm{d}\widetilde{\varvec{\mu }}^k}{\, \textrm{d}\mathscr {L}^{d+1}}(S_k^{-1}(t),x), \frac{\, \textrm{d}\widetilde{{\varvec{\nu }}}^k}{\, \textrm{d}\mathscr {L}^{d+1}}(S_k^{-1}(t),x) \Big ) \, \textrm{d}\mathscr {L}^{d+1} \\&\le (1-\delta _k)(1+4\eta _k) \mathbb {A}_\textrm{hom}^{(\alpha _k, T- \alpha _k)}(\widetilde{\varvec{\mu }}^k, \widetilde{{\varvec{\nu }}}^k) \le (1-\delta _k) (1+4\eta _k) \mathbb {A}_\textrm{hom}^T({\varvec{\mu }}, {\varvec{\nu }}), \end{aligned}$$

where in the last inequality we used (8.37). Taking the limsup in $k \rightarrow \infty$

8.38 $$\begin{aligned} \limsup _{k \rightarrow +\infty } \mathbb {A}_\textrm{hom}^{\mathcal {I}_k}({\varvec{\mu }}^k,{\varvec{\nu }}^k) \le \mathbb {A}_\textrm{hom}^T ({\varvec{\mu }}, {\varvec{\nu }}) \end{aligned}$$

which concludes the proof of (8.31).

Now we are ready to prove the limsup inequality (5.2) in Theorem 5.1.

#### Proof of Theorem 5.1 (upper bound)

Fix ${\varvec{\mu }}\in \mathcal {M}_+\big (\mathcal {I}\times \mathbb {T}^d\big )$. By definition of $\mathbb {A}_\textrm{hom}^\mathcal {I}({\varvec{\mu }})$, it suffices to prove that for every ${\varvec{\nu }}\in \mathcal {M}^d(\mathcal {I}\times \mathbb {T}^d)$ such that $({\varvec{\mu }},{\varvec{\nu }}) \in \mathbb{C}\mathbb{E}^T$ and $\mathbb {A}_\textrm{hom}^\mathcal {I}({\varvec{\mu }},{\varvec{\nu }}) < +\infty$, we can find ${\pmb {m}}^\varepsilon : \overline{\mathcal {I}} \rightarrow \mathbb {R}_+^{\mathcal {X}_\varepsilon }$ such that $\iota _\varepsilon {\pmb {m}}^\varepsilon \rightarrow {\varvec{\mu }}$ weakly in $\mathcal {M}_+(\mathcal {I}\times \mathbb {T}^d)$ and $\limsup _\varepsilon \mathcal {A}_\varepsilon ^\mathcal {I}({\pmb {m}}^\varepsilon ) \le \mathbb {A}_\textrm{hom}^\mathcal {I}({\varvec{\mu }},{\varvec{\nu }})$.

For any such $({\varvec{\mu }}, {\varvec{\nu }})$, we apply Proposition 8.26 and find a smooth sequence $({\varvec{\mu }}^k,{\varvec{\nu }}^k)_k \in \mathbb{C}\mathbb{E}^{\mathcal {I}(k)}$ where $\mathcal {I}(k) = (-\eta _k, T+ \eta _k)$, where $\eta _k \rightarrow 0$ and such that (8.31) and (8.32) hold with $({\varvec{\mu }}^k, {\varvec{\nu }}^k) \rightarrow ({\varvec{\mu }},{\varvec{\nu }})$ weakly in $\mathcal {M}_+(\mathcal {I}\times \mathbb {T}^d) \times \mathcal {M}^d(\mathcal {I}\times \mathbb {T}^d)$ as $k \rightarrow +\infty$. In particular

8.39 $$\begin{aligned} \sup _{k \in \mathbb {N}} \sup _{t \in \mathcal {I}} \mu _t^k(\mathbb {T}^d) = \sup _{k \in \mathbb {N}} \mu _0^k(\mathbb {T}^d) <\infty . \end{aligned}$$

Hence we can apply Proposition 8.23 and find ${\pmb {m}}^{\varepsilon ,k} \in \mathcal {M}_+(\overline{\mathcal {I}} \times \mathbb {T}^d)$ such that $\iota _\varepsilon {\pmb {m}}^{\varepsilon ,k} \rightarrow {\varvec{\mu }}^k$ weakly in $\mathcal {M}_+(\overline{\mathcal {I}} \times \mathbb {T}^d)$ and for each $k \in \mathbb {N}$,

8.40 $$\begin{aligned} \limsup _{\varepsilon \rightarrow 0} \mathcal {A}_\varepsilon ^\mathcal {I}({\pmb {m}}^{\varepsilon ,k}) \le \mathbb {A}_\textrm{hom}^{\mathcal {I}(k)}({\varvec{\mu }}^k, {\varvec{\nu }}^k) + C \eta _k |\mathcal {I}|\big (\mu _0^k(\mathbb {T}^d) + 1\big ). \end{aligned}$$

We conclude by extracting a subsequence ${\pmb {m}}^\varepsilon := {\pmb {m}}^{\varepsilon ,k(\varepsilon )}$ such that $\iota _\varepsilon {\pmb {m}}^\varepsilon \rightarrow {\varvec{\mu }}$ weakly in $\mathcal {M}_+(\mathcal {I}\times \mathbb {T}^d)$ as $\varepsilon \rightarrow 0$ and from (8.39), (8.40), (8.31) we have

$$\begin{aligned} \limsup _{\varepsilon \rightarrow 0} \mathcal {A}_\varepsilon ^\mathcal {I}({\pmb {m}}^\varepsilon ) \le \mathbb {A}_\textrm{hom}^\mathcal {I}({\varvec{\mu }},{\varvec{\nu }}), \end{aligned}$$

which concludes the proof.

## Analysis of the cell problem

In the final section of this work, we discuss some properties of the limit functional $\mathbb {A}_\textrm{hom}$ and analyse examples where explicit computations can be performed. For $\rho \in \mathbb {R}_+$ and $j \in \mathbb {R}^d$, recall that

$$\begin{aligned} \begin{aligned} f_{\textrm{hom}}(\rho ,j)&:= \inf \big \{ F(m,J) \ : \ (m,J) \in {{\,\mathrm{{\textsf{Rep}}}\,}}(\rho ,j) \big \}, \end{aligned} \end{aligned}$$

where ${{\,\mathrm{{\textsf{Rep}}}\,}}(\rho ,j)$ denotes the set of representatives of $(\rho ,j)$, i.e., all $\mathbb {Z}^d$-periodic functions $m \in \mathbb {R}_+^\mathcal {X}$ and $J \in \mathbb {R}_a^\mathcal {E}$ satisfying

$$\begin{aligned} \sum _{x \in \mathcal {X}^Q} m(x) = \rho , \quad {{\,\mathrm{\textsf{Eff}}\,}}(J) = \frac{1}{2} \sum _{(x,y) \in \mathcal {E}^Q} J(x,y) (y_\textsf{z}- x_\textsf{z}) = j , \quad \text { and } \quad {{\,\mathrm{\text {\textsf{div}}}\,}}J \equiv 0. \end{aligned}$$

### Invariance under rescaling

We start with an invariance property of the cell-problem. Fix a $\mathbb {Z}^d$-periodic graph $(\mathcal {X}, \mathcal {E})$ as defined in Assumption 2.1. For fixed $\varepsilon > 0$ with $\varepsilon \in \frac{1}{\mathbb {N}}$, we consider the rescaled $\mathbb {Z}^d$-periodic graph $(\widetilde{\mathcal {X}}, \widetilde{\mathcal {E}})$ obtained by zooming out by a factor $\frac{1}{\varepsilon }$, so that each unit cube contains $(\frac{1}{\varepsilon })^d$ copies of $\mathcal {X}^Q$. Slightly abusing notation, we will identify the corresponding set $\widetilde{V}$ with the points in $\mathbb {T}_\varepsilon ^d$.

Let $\widetilde{F}$ be the analogue of *F* on $(\widetilde{\mathcal {X}}, \widetilde{\mathcal {E}})$, and let $\widetilde{f}_\textrm{hom}$ be the corresponding limit density. In view of our convergence result, the cell-formula must be *invariant* under rescaling, namely $f_\textrm{hom}= \widetilde{f}_\textrm{hom}$. We will verify this identity using a direct argument that crucially uses the convexity of *F*.

One inequality follows from the natural inclusion of representatives

9.1 $$\begin{aligned} {{\,\mathrm{{\textsf{Rep}}}\,}}(\rho ) \hookrightarrow \varepsilon ^d \widetilde{{\,\mathrm{{\textsf{Rep}}}\,}}(\rho ), \quad {{\,\mathrm{{\textsf{Rep}}}\,}}(j) \hookrightarrow \varepsilon ^{d-1} \widetilde{{\,\mathrm{{\textsf{Rep}}}\,}}(j), \end{aligned}$$

which is obtained as $\widetilde{m}:=\varepsilon ^d (\tau _\varepsilon ^0)^{-1}(m)$ and $\widetilde{J}:= \varepsilon ^{d-1} (\tau _\varepsilon ^0)^{-1} (J)$ for every $(m,J) \in {{\,\mathrm{{\textsf{Rep}}}\,}}(\rho ,j)$. Here we note that the inverse of $\tau _\varepsilon ^0$ is well-defined on $\mathbb {Z}^d$-periodic maps. In particular we have

$$\begin{aligned} \sum _{x \in {\widetilde{X}}^Q} \widetilde{m}(x) = \sum _{x \in \mathcal {X}^Q} m(x) = \rho , \quad {{\,\mathrm{\textsf{Eff}}\,}}(\widetilde{J}) = {{\,\mathrm{\textsf{Eff}}\,}}(J) , \quad \text { and }\quad \widetilde{F}(\widetilde{m}, \widetilde{J}) = F(m,J), \end{aligned}$$

which implies that $f_\textrm{hom}\ge \widetilde{f}_\textrm{hom}$.

The opposite inequality is where the convexity of *F* comes into play. Pick $(\widetilde{m}, \widetilde{J}) \in \widetilde{{\,\mathrm{{\textsf{Rep}}}\,}}(\rho ,j)$. A first attempt to define a couple in ${{\,\mathrm{{\textsf{Rep}}}\,}}(\rho ,j)$ would be to consider the inverse map of what we did in (9.1), but the resulting maps would not be $\mathbb {Z}^d$-periodic (but only $\frac{1}{\varepsilon }\mathbb {Z}^d$-periodic). What we can do is to consider a convex combination of such values. Precisely, we define

$$\begin{aligned} m(x) := \varepsilon ^d \sum _{z \in \mathbb {Z}_\varepsilon ^d} \frac{\tau _\varepsilon ^z \widetilde{m}(x)}{\varepsilon ^d} \quad \text { and }\quad J(x,y) := \varepsilon ^d \sum _{z \in \mathbb {Z}_\varepsilon ^d} \frac{\tau _\varepsilon ^z \widetilde{J}(x,y)}{\varepsilon ^{d-1}} \end{aligned}$$

for all $(x,y) \in \mathcal {X}^Q$. The linearity of the constraints implies that $(m,J) \in {{\,\mathrm{{\textsf{Rep}}}\,}}(\rho ,j)$. Moreover, using the convexity of *F* we obtain

$$\begin{aligned} F(m,J) = F \bigg ( \varepsilon ^d \sum _{z \in \mathbb {Z}_\varepsilon ^d} \bigg ( \frac{\tau _\varepsilon ^z \widetilde{m}}{\varepsilon ^d} , \frac{\tau _\varepsilon ^z \widetilde{J}}{\varepsilon ^{d-1}} \bigg ) \bigg ) \le \sum _{z \in \mathbb {Z}_\varepsilon ^d} \varepsilon ^d F\bigg ( \frac{\tau _\varepsilon ^z m}{\varepsilon ^d} , \frac{\tau _\varepsilon ^z J}{\varepsilon ^{d-1}} \bigg ) = \widetilde{F}(\widetilde{m}, \widetilde{J}), \end{aligned}$$

which in particular proves that $f_\textrm{hom}\le \widetilde{f}_\textrm{hom}$.

### The simplest case: $V= \{v\}$ and nearest-neighbor interaction.

The easiest example we can consider is the one where the set *V* consists of only one element $v \in V$. In other words, we focus on the case when $\mathcal {X}\simeq \mathbb {Z}^d$ and thus $\mathcal {X}_\varepsilon \simeq \mathbb {T}_\varepsilon ^d$. We then consider the graph structure defined via the *nearest-neighbor interaction*, meaning that $\mathcal {E}$ consists of the elements of $(x,y) \in \mathbb {Z}^d \times \mathbb {Z}^d$ such that $|x-y|_\infty = 1$.

In this setting, $\mathcal {X}^Q\simeq V$ consists of only one element and $\mathcal {E}^Q\simeq \left\{ (v, v \pm e_i) \,: \, i=1, \dots , d \right\}$ has cardinality 2*d*. In particular, for every $\rho \in \mathbb {R}_+$ and $j \in \mathbb {R}^d$, the set ${{\,\mathrm{{\textsf{Rep}}}\,}}(\rho , j)$ consists of only one element $(\underline{m}, \underline{J})$ given by

$$\begin{aligned} \underline{m} (x) = \rho , \quad \underline{J}(v, v \pm e_i) = \pm j_i, \quad \text {for all}\ (x,y) \in \mathcal {E}\ \ \text {and} \ \ i = 1, \dots , d. \end{aligned}$$

Consequently, the homogenised energy density is given by $f_\textrm{hom}(\rho ,j) = F(\underline{m}, \underline{J})$.

In the special case where *F* is edge-based (see Remark 2.5) with edge-energies $\{ F_{\pm i} \}$ for $i = 1, \ldots , d$, we have

$$\begin{aligned} F(m, J )&= \sum _{i = 1}^d F_i\big ( m(0), m(e_i), J(0, e_i) \big ) + F_{-i}\big ( m(0), m(- e_i), J(0, - e_i) \big ), \quad \text {and} \\ f_\textrm{hom}(\rho ,j)&= \sum _{i = 1}^d F_i\big ( \rho , \rho , j_i \big ) + F_{-i}\big ( \rho , \rho , -j_i \big ) \text { for all } \quad \rho \in \mathbb {R}_+ , \; j \in \mathbb {R}^d. \end{aligned}$$

The even more special case of the discretised *p*-Wasserstein distance corresponds to $F_i(\rho _1, \rho _2, j) = \frac{|j|^p}{2\Lambda (\rho _1,\rho _2)^{p-1}}$, where the mean $\Lambda$ is a mean as in (2.3). We then obtain

$$\begin{aligned} f_\textrm{hom}(\rho ,j) = \frac{|j|_p^p}{\rho ^{p-1}}, \end{aligned}$$

for $\rho \in \mathbb {R}_+$ and $j \in \mathbb {R}^d$, which corresponds to the *p*-Wasserstein distance induced by the $\ell _p$-distance $| \cdot |_p$ on the underlying space $\mathbb {T}^d$. The case $p = 2$ corresponds to the framework studied in [24].

As we will discuss in Sect. 9.4, this result can also be cast in the more general framework of isotropic finite-volume partitions of $\mathbb {T}^d$.

### Embedded graphs

In this section, we shall use an equivalent *geometric* definition of the effective flux. We can indeed formulate an interesting expression for $$f_\textrm{hom}$$ in the case where $$(\mathcal {X},\mathcal {E})$$ is an embedded $$\mathbb {Z}^d$$-periodic graph in $$\mathbb {T}^d$$, in the sense of Remark 2.2. We thus choose $$V$$ to be a subset of $$[0,1)^d$$ and use the identification $$(z, v) \equiv z + v$$, so that $$\mathcal {X}$$ can be identified with a $$\mathbb {Z}^d$$-periodic subset of $$\mathbb {R}^d$$.

Let us define

$$\begin{aligned} \textsf{Eff}_{\textrm{geo}}(J) := \frac{1}{2} \sum _{(x,y) \in \mathcal {E}^Q} J(x,y) \big ( y - x \big ). \end{aligned}$$

Note that we simply replaced $y_\textsf{z}- x_\textsf{z}\in \mathbb {Z}$ by $y - x \in \mathbb {R}^d$ in the definition of ${{\,\mathrm{\textsf{Eff}}\,}}(J)$. Remarkably, the following result shows that ${{\,\mathrm{\textsf{Eff}}\,}}(J) = \textsf{Eff}_{\textrm{geo}}(J)$ for any periodic and divergence-free vector field *J*. In particular, $\textsf{Eff}_{\textrm{geo}}(J)$ does *not* depend on the choice of the embedding into $\mathbb {T}^d$. As a consequence, one can equivalently define ${{\,\mathrm{{\textsf{Rep}}}\,}}(j)$, and hence the homogenised energy density $f_\textrm{hom}(\rho , j)$, in terms of $\textsf{Eff}_{\textrm{geo}}(J)$ instead of ${{\,\mathrm{\textsf{Eff}}\,}}(J)$.

#### Proposition 9.1

For every periodic and divergence-free vector field $J \in \mathbb {R}_a^\mathcal {E}$ we have ${{\,\mathrm{\textsf{Eff}}\,}}(J) = \textsf{Eff}_{\textrm{geo}}(J)$.

#### Proof

Note first that any given point configuration can be transformed into any other configuration by successively shifting each of the points. Therefore it suffices to show that $\textsf{Eff}_{\textrm{geo}}(J)$ is invariant when perturbing the location of any single point.

Fix $x_0 \in \mathcal {X}^Q$. For a positive (small enough) parameter $t > 0$ and a vector $v \in \mathbb {R}^d$, consider the modified embedded $\mathbb {Z}^d$-periodic graph $(\mathcal {X}(t),\mathcal {E}(t))$ in $\mathbb {T}^d$ obtained from $\mathcal {X}$ by *shifting* the nodes $x_0 + \mathbb {Z}^d$ by $t v \in \mathbb {T}^d$, i.e., we consider the shifted node $x_0(t):= x_0 + t v$ instead of $x_0$ (and with it, the associated edges). Fix a divergence-free and $\mathbb {Z}^d$-periodic discrete vector field $J \in \mathbb {R}_a^\mathcal {E}\simeq \mathbb {R}_a^{\mathcal {E}(t)}$ and consider, for $t > 0$, the corresponding effective flux

$$\begin{aligned} \textsf{Eff}_{\textrm{geo}}(t,J) := \frac{1}{2} \sum _{(x,y) \in \mathcal {E}^Q(t)} J(x,y) \big ( y - x \big ). \end{aligned}$$

We claim that $\frac{\textrm{d}}{\textrm{d}t} \textsf{Eff}_{\textrm{geo}}(t,J) = 0$. Indeed, by construction we have

$$\begin{aligned} 2\frac{\textrm{d}}{\textrm{d}t} \textsf{Eff}_{\textrm{geo}}(t,J)&= - \sum _{y \sim x_0} J(x_0,y) v + \sum _{z \in \mathbb {Z}^d} \sum _{\begin{array}{c} x \in \mathcal {X}^Q\\ x \sim x_0 + z \end{array}} J(x, x_0 + z) v \\&{\mathop {=}\limits ^{J \text { per.}}} - {{\,\mathrm{\text {\textsf{div}}}\,}}J(x_0) v + \sum _{z \in \mathbb {Z}^d} \sum _{\begin{array}{c} x \in \mathcal {X}^Q\\ x - z \sim x_0 \end{array}} J(x - z, x_0) v \\&= - {{\,\mathrm{\text {\textsf{div}}}\,}}J(x_0) v + \sum _{x' \sim x_0} J(x',x_0) v \\ {}&= - {{\,\mathrm{\text {\textsf{div}}}\,}}J(x_0) v + \sum _{x' \sim x_0} J(x_0,x') v = -2 {{\,\mathrm{\text {\textsf{div}}}\,}}J(x_0) v. \end{aligned}$$

Since *J* is divergence-free, this proves the claim. In particular, $$t \mapsto \textsf{Eff}_{\textrm{geo}}(t,J)$$ is constant, hence the value of $$\textsf{Eff}_{\textrm{geo}}$$ does not depend on the location of the embedded points. This also implies the sought equality $${{\,\mathrm{\textsf{Eff}}\,}}(J) = \textsf{Eff}_{\textrm{geo}}(J)$$, since $${{\,\mathrm{\textsf{Eff}}\,}}(J)$$ corresponds to the limiting case where all the elements of *V* “collapse” into a single point of $$[0,1)^d$$.

### Periodic finite-volume partitions

The next class of examples are the graph structures associated with $\mathbb {Z}^d$-periodic *finite-volume partitions* (FVPs) $\mathcal {T}$ of $\mathbb {R}^d$. We refer to [14] for a general treatment.

#### Definition 9.2

($\mathbb {Z}^d$-periodic finite-volume partition) Consider a countable, locally finite, $\mathbb {Z}^d$-periodic family of points $\mathcal {X}\subseteq \mathbb {R}^d$ together with a family of nonempty open bounded convex polytopes $K_x \subseteq \mathbb {R}^d$ for $x \in \mathcal {X}$, such that $K_{x+z} = K_x + z$ for all $x \in \mathcal {X}$ and $z \in \mathbb {Z}^d$. We call

$$\begin{aligned} \mathcal {T}:= \Big \{ (x, K_x) \ : \ x \in \mathcal {X}\Big \} \end{aligned}$$

a $\mathbb {Z}^d$-*periodic finite-volume partition* of $\mathbb {R}^d$ if

1. $$\bigcup _{x\in \mathcal {X}} \overline{K_x} = \mathbb {R}^d$$;
2. $$K_x \cap K_y = \emptyset $$ whenever $$x\ne y \in \mathcal {X}$$;
3. $$y-x \perp \partial K_x \cap \partial K_y$$ whenever $$\mathscr {H}^{d-1}(\partial K_x \cap \partial K_y) > 0$$.

We define a graph structure on $\mathcal {X}$ by declaring those pairs $(x,y)\in \mathcal {X}\times \mathcal {X}$ with $\mathscr {H}^{d-1}(\partial K_x \cap \partial K_y) > 0$ to be nearest neighbors.

It is not difficult to see that the graph $(\mathcal {X}, \mathcal {E})$ is connected, $\mathbb {Z}^d$-periodic, and locally finite, even if $x \notin K_x$. Throughout this section we use the following notation for $x, y \in \mathcal {X}$:

$$\begin{aligned} |K_x|&:= \mathscr {L}^d(K_x),&d_{xy}&:= |y-x|, \\ s_{xy}&:= \mathscr {H}^{d-1}(\partial K_x \cap \partial K_y),&n_{xy}&:= \frac{y-x}{d_{xy}} \in \mathcal {S}^{d-1}. \end{aligned}$$

In the finite-volume framework, we are interested in transport distances with a nonlinear mobility. These distances were introduced in [13] as natural generalisations of the 2-Wasserstein metric. We thus fix a concave upper-semicontinuous function $\mathfrak {m}: \mathbb {R}_+ \times \mathbb {R}^d \rightarrow \mathbb {R}_+$ and consider the energy density functional

9.2 $$\begin{aligned} f(\rho , j) := {\left\{ \begin{array}{ll} \frac{|j|_2^2}{\mathfrak {m}(\rho )} &{} \text { if } \mathfrak {m}(\rho ) > 0,\\ +\infty &{} \text { if }\mathfrak {m}(\rho ) = 0 \text { and } j \ne 0,\\ 0 &{} \text { if } \mathfrak {m}(\rho ) = 0 \text { and } j = 0. \end{array}\right. } \end{aligned}$$

To discretise this energy density, we fix for every edge $(x,y) \in \mathcal {E}$ an *admissible version* of $\mathfrak {m}:\mathbb {R}_+\rightarrow \mathbb {R}_+$, i.e., a nonnegative concave upper-semicontinuous function $\mathfrak {m}_{xy}: \mathbb {R}_+ \times \mathbb {R}_+ \rightarrow \mathbb {R}_+$ satisfying $\mathfrak {m}_{xy}(\rho ,\rho ) = \mathfrak {m}(\rho )$ for all $\rho \in \mathbb {R}_+$ and $(x,y)\in \mathcal {E}$. We always assume that $\mathfrak {m}_{xy}(\rho _1,\rho _2) = \mathfrak {m}_{yx}(\rho _2,\rho _1)$ for all $\rho _1, \rho _2 \in \mathbb {R}_+$. It is easy to check that *F* satisfies the superlinear growth condition 5.4. Furthermore, concavity of $\mathfrak {m}_{xy}$ implies convexity of *F*.^6^ We then consider the edge-based cost defined by

9.3 $$\begin{aligned} F(m,J) := \frac{1}{2} \sum _{(x,y) \in \mathcal {E}^Q} \frac{d_{xy}}{s_{xy}} \frac{J(x,y)^2}{\mathfrak {m}_{xy} \Big ( \frac{m(x)}{|K_x|}, \frac{m(y)}{|K_y|} \Big )}, \end{aligned}$$

Consistent with (9.2), we use the convention that

9.4 $$\begin{aligned} \frac{J(x,y)^2}{\mathfrak {m}_{xy} \Big ( \frac{m(x)}{|K_x|}, \frac{m(y)}{|K_y|} \Big ) } = {\left\{ \begin{array}{ll} + \infty &{} \text { if } \mathfrak {m}_{xy} \Big ( \frac{m(x)}{|K_x|}, \frac{m(y)}{|K_y|} \Big ) = 0 \text { and } J(x,y) \ne 0, \\ 0 &{} \text { if } \mathfrak {m}_{xy} \Big ( \frac{m(x)}{|K_x|}, \frac{m(y)}{|K_y|} \Big ) = 0 \text { and } J(x,y) = 0. \end{array}\right. } \end{aligned}$$

It is now natural to ask whether the discrete action functionals associated to *F* converge to the continuous action funtional associated to *f*: is it true that $f_\textrm{hom}= f$?

In the linear case where $\mathfrak {m}(\rho ) = \rho$, which corresponds to the 2-Wasserstein metric, this question has been extensively studied in [26] for a large class of (not necessarily periodic) meshes. The main result in [26] asserts that the limit of the discrete transport distances $\mathcal {W}_\varepsilon$ (in the Gromov-Hausdorff sense) as $\varepsilon \rightarrow 0$ coincides with the 2-Wasserstein distance $\mathbb {W}_2$ on $\mathcal {P}(\mathbb {T}^d)$ if an asymptotic local isotropy condition is satisfied. Moreover, it is shown that this convergence fails to hold if the isotropy condition fails to hold (in a sufficiently strong sense).

For periodic finite-volume partitions we show here that these results are direct consequences of Theorem 5.1. In particular, the following result contains a necessary and condition on a periodic finite-volume partition that ensures that $f_\textrm{hom}= f$.

#### Proposition 9.3

Consider a $\mathbb {Z}^d$-periodic finite-volume partition of $\mathbb {R}^d$, and let *F* and *f* be as in (9.2) and (9.3) respectively. The following assertions hold:

(i) $f_\textrm{hom}(\rho ,j) \le f(\rho ,j)$ for all $\rho \in \mathbb {R}_+$ and $j\in \mathbb {R}^d$.
(ii) Suppose that for every $\rho \in \mathbb {R}_+$ and $j\in \mathbb {R}^d$ there is a family of vectors $(p^{xy})_{(x,y) \in \mathcal {E}} \subseteq \mathbb {R}^2$ such that
  9.5 $$\begin{aligned}&p^{xy} = (p^{xy}_1,p^{xy}_2) \in \partial ^+ \mathfrak {m}_{xy}(\rho ,\rho ){} & {} \text {for all } (x,y) \in \mathcal {E}, \text { and } \end{aligned}$$
  9.6 $$\begin{aligned}&\frac{1}{|K_x|} \sum _{y \sim x} (p^{xy}_1 + p^{yx}_2) d_{xy} s_{xy} (n_{xy} \cdot j)^2{} & {} \text {is independent of } x \in \mathcal {X}. \end{aligned}$$
  Then: $$f_\textrm{hom}= f$$.
(iii) Suppose that all $\mathfrak {m}_{xy}$ are differentiable in a neigbourhood of the diagonal in $(0,\infty )^2$. Then $f_\textrm{hom}= f$ if and only if
  9.7 $$\begin{aligned} \sum _{y \sim x} \frac{\partial _1\mathfrak {m}_{xy}(\rho ,\rho )}{\mathfrak {m}'(\rho ) } d_{xy} s_{xy} n_{xy} \otimes n_{xy} = |K_x| {{\,\textrm{id}\,}}\quad \text { for all } x \in \mathcal {X}\text { and } \rho > 0. \end{aligned}$$

#### Remark 9.4

The condition (*iii*) is satisfied for a class of meshes satisfying a weighted isotropy condition. For given edge weights $\lambda _{xy} \in (0,1)$, this condition reads as

$$\begin{aligned} \sum _{y \sim x} \lambda _{x y} d_{xy} s_{xy} n_{xy} \otimes n_{xy} = |K_x| {{\,\textrm{id}\,}}\quad \text { for all } x \in \mathcal {X}. \end{aligned}$$

We refer to [26, Definition 1.4] for this notion on domains in $\mathbb {R}^d$ and to [25, Definition 4.3] for the one-dimensional periodic setting. In this case, given a mobility function $\mathfrak {m}$, the functions $\mathfrak {m}_{xy}$ can be chosen to be of the form $\mathfrak {m}_{xy}(\rho , \rho ') = \mathfrak {m}(\theta _{xy}(\rho ,\rho '))$ where $\theta _{xy}$ is a mean that is compatible with $\lambda _{xy}$ in the sense that $\partial _1\theta _{xy}(1,1) = \lambda _{xy}$; see [26, Definition 1.4]. In this situation the identity $f = f_\textrm{hom}$ holds for all choices of the mobility $\mathfrak {m}$, since $\partial _1\mathfrak {m}_{xy}(\rho ,\rho ) = \mathfrak {m}'(\rho ) \partial _1\theta _{xy}(\rho , \rho ) = \mathfrak {m}'(\rho ) \lambda _{xy}$. Therefore, the condition (9.7) reduces to the isotropy condition above; in particular, it does not depend on $\mathfrak {m}$.

Before we prove Proposition 9.3, we first show an elementary identity for finite-volume partitions; see also [26, Lemma 5.4] for a similar result in a non-periodic setting.

#### Lemma 9.5

Let $\mathcal {T}$ be a $\mathbb {Z}^d$-periodic finite-volume partition of $\mathbb {R}^d$. Then

9.8 $$\begin{aligned} \frac{1}{2} \sum _{(x,y)\in \mathcal {E}^Q} d_{xy} s_{xy} n_{xy} \otimes n_{xy} = {{\,\textrm{id}\,}}. \end{aligned}$$

#### Proof

For $v\in \mathbb {R}^d{\setminus } \{0\}$ and $(x,y)\in \mathcal {E}$, consider the open bounded convex polytope

$$\begin{aligned} C_{xy}:= \big \{ z \in (\partial K_x \cap \partial K_y) + \mathbb {R}v:\,z \cdot v \in \big (\textrm{conv}(x\cdot v, y\cdot v)\big )^\circ \big \}. \end{aligned}$$

Note that $C_{xy} = C_{yx}$. We claim that the family $\{C_{xy}:\,(x,y)\in \mathcal {E}\}$ forms a partition of $\mathbb {R}^d$ up to a set of Lebesgue measure zero. To see this, fix a point $z \in \mathbb {R}^d$ and consider the function $X: \mathbb {R}\rightarrow \mathcal {X}$ defined by $X(t) = x$ if $z + t v \in K_x$. If *v* is not orthogonal to any of the finitely many $n_{xy}$, then *X*(*t*) is well-defined up to a countable set $N \subset \mathbb {R}$. By Fubini’s theorem, it follows that $\mathscr {L}^d\big ( \mathbb {R}^d {\setminus } \bigcup _{(x,y)\in \mathcal {E}} C_{xy} \big ) = 0$.

If $t \in N$ and $X(t^-) = x$, $X(t^+) = y$, then $(y-x)\cdot v = d_{xy} n_{xy} \cdot v > 0$. This shows that $t\mapsto v\cdot X(t)$ is nondecreasing and that *z* is in at most one parallelepiped.

On the other hand, we have

$$\begin{aligned} \mathscr {L}^d(C_{xy}) = d_{xy} s_{xy} \left( n_{xy} \cdot \frac{v}{|v|}\right) ^2. \end{aligned}$$

Then we have

$$\begin{aligned} 1&= \frac{1}{2} \sum _{x\in \mathcal {X}} \sum _{y\sim x} \mathscr {L}^d(C_{xy} \cap [0,1)^d) = \frac{1}{2} \sum _{x\in \mathcal {X}^Q} \sum _{y\sim x} \mathscr {L}^d(C_{xy}) \\ {}&= \frac{1}{2}\sum _{(x,y)\in \mathcal {E}^Q} d_{xy} s_{xy} \bigg (n_{xy} \cdot \frac{v}{|v|}\bigg )^2 = \frac{v}{|v|} \cdot \bigg (\frac{1}{2} \sum _{(x,y)\in \mathcal {E}^Q} d_{xy} s_{xy} n_{xy} \otimes n_{xy}\bigg ) \frac{v}{|v|}. \end{aligned}$$

Since this identity holds for almost every $v \in \mathbb {R}^d$, (9.8) holds by polarization.

#### Proof of Proposition 9.3

*(i)*: We construct a competitor $(m^\star ,J^\star )$ to the cell problem (4.6) for $\rho \in \mathbb {R}_+$ and $j \in \mathbb {R}^d$. Define

9.9 $$\begin{aligned} m^\star (x) := |K_x|\rho \quad \text { and }\quad J^\star (x,y) := s_{xy} (j \cdot n_{xy}). \end{aligned}$$

We claim that $(m^\star , J^\star )\in {{\,\mathrm{{\textsf{Rep}}}\,}}(\rho ,j)$. Indeed, the periodicity of $\mathcal {T}$ yields

$$\begin{aligned} \sum _{x\in \mathcal {X}^Q} m^\star (x) = \rho \sum _{x\in \mathcal {X}^Q} |K_x| = \rho \mathscr {L}^d([0,1)^d) = \rho , \end{aligned}$$

which shows that $m^\star \in {{\,\mathrm{{\textsf{Rep}}}\,}}(\rho )$. To show that $J^\star \in {{\,\mathrm{{\textsf{Rep}}}\,}}(j)$, we use the divergence theorem to obtain, for $x \in \mathcal {X}$,

$$\begin{aligned} {{\,\mathrm{\text {\textsf{div}}}\,}}J^\star (x) = \sum _{y\sim x} J^\star (x,y) = \sum _{y\sim x} s_{xy} (j \cdot n_{xy}) = \int _{\partial K_x} j \cdot n \, \textrm{d}\mathscr {H}^{d-1} = 0. \end{aligned}$$

Moreover, using Proposition 9.1 and Lemma 9.5 we find

$$\begin{aligned} {{\,\mathrm{\textsf{Eff}}\,}}(J^\star )&= \frac{1}{2} \sum _{(x,y)\in \mathcal {E}^Q} J^\star (x,y) (y_\textsf{z}- x_\textsf{z}) = \frac{1}{2} \sum _{(x,y)\in \mathcal {E}^Q} J^\star (x,y) (y - x) \\&= \frac{1}{2} \sum _{(x,y)\in \mathcal {E}^Q} s_{xy} (j \cdot n_{xy}) (y-x) = \frac{1}{2} \sum _{(x,y)\in \mathcal {E}^Q} d_{xy} s_{xy} (n_{xy} \otimes n_{xy}) j = j, \end{aligned}$$

which proves that $J^\star \in {{\,\mathrm{{\textsf{Rep}}}\,}}(j)$. Therefore, using that $\mathfrak {m}_{xy}$ is an admissible version of $\mathfrak {m}$, another application of Lemma 9.5 yields (taking (9.4) into account),

$$\begin{aligned} f_\textrm{hom}(\rho ,j)&\le F(m^\star , J^\star ) = \frac{1}{2} \sum _{(x,y)\in \mathcal {E}^Q} \frac{d_{xy}}{s_{xy}} \frac{J^\star (x,y)^2}{\mathfrak {m}_{xy}(\rho ,\rho )} \\ {}&= \frac{1}{\mathfrak {m}(\rho )}j \cdot \left( \frac{1}{2} \sum _{(x,y)\in \mathcal {E}^Q} d_{xy} s_{xy} n_{xy} \otimes n_{xy} \right) j = \frac{|j|^2}{\mathfrak {m}(\rho )} = f(\rho , j), \end{aligned}$$

which proves *(i)*.

*(iii)*: Suppose first that condition (9.7) holds. We will show that $(m^\star , J^\star )$ is a critical point of *F*. Take $(\widetilde{m}, \widetilde{J}) \in {{\,\mathrm{{\textsf{Rep}}}\,}}(0,0)$ and define, for $\varepsilon > 0$ sufficiently small,

$$\begin{aligned} m_\varepsilon := m^\star + \varepsilon \widetilde{m} \quad \text { and }\quad J_\varepsilon := J^\star + \varepsilon \widetilde{J}. \end{aligned}$$

Then:

$$\begin{aligned} \partial _\varepsilon \big |_{\varepsilon = 0} F(m^\star ,J_\varepsilon )&= \frac{1}{2} \partial _\varepsilon \big |_{\varepsilon = 0} \sum _{(x,y)\in \mathcal {E}^Q} \frac{d_{xy}}{s_{xy}} \frac{J^\star (x,y)^2}{\mathfrak {m}_{xy}(\rho ,\rho )} = \frac{1}{\mathfrak {m}(\rho )} \sum _{(x,y)\in \mathcal {E}^Q} \frac{d_{xy}}{s_{xy}} J^\star (x,y) \widetilde{J}(x,y) \\ {}&= \frac{1}{\mathfrak {m}(\rho )} \sum _{(x,y)\in \mathcal {E}^Q} j \cdot (y-x) \widetilde{J}(x,y) = \frac{1}{\mathfrak {m}(\rho )} j \cdot {{\,\mathrm{\textsf{Eff}}\,}}(\widetilde{J}) = 0. \end{aligned}$$

Furthermore, using the symmetry $\mathfrak {m}_{xy}(a,b) = \mathfrak {m}_{yx}(b,a) =$ for $a, b \ge 0$, we obtain

9.10 $$\begin{aligned} \begin{aligned} \partial _\varepsilon \big |_{\varepsilon = 0} F(m_\varepsilon , J^\star )&= - \frac{1}{2} \sum _{(x,y)\in \mathcal {E}^Q} \frac{d_{xy}}{s_{xy}} \frac{J^\star (x,y)^2}{\mathfrak {m}(\rho )^2} \bigg ( \frac{\widetilde{m}(x)}{|K_x|} \partial _1 \mathfrak {m}_{xy}(\rho , \rho ) + \frac{\widetilde{m}(y)}{|K_y|} \partial _2 \mathfrak {m}_{xy}(\rho , \rho ) \bigg ) \\ {}&= - \sum _{(x,y)\in \mathcal {E}^Q} \frac{d_{xy}}{s_{xy}} \frac{J^\star (x,y)^2}{\mathfrak {m}(\rho )^2} \frac{\widetilde{m}(x)}{|K_x|} \partial _1 \mathfrak {m}_{xy}(\rho , \rho ) \\ {}&= - \frac{\mathfrak {m}'(\rho )}{\mathfrak {m}^2(\rho )} |j|^2 \sum _{x \in \mathcal {X}^Q} b_x(\rho , j) \frac{ \widetilde{m}(x) }{ |K_x| } , \end{aligned} \end{aligned}$$

where we write $b_{x}(\rho , j):= \sum _{(x,y \in \mathcal {E}^Q} \frac{\partial _1 \mathfrak {m}_{xy}(\rho , \rho )}{\mathfrak {m}'(\rho )} d_{xy} s_{xy} \frac{(n_{xy} \cdot j)^2}{|j|^2}$, so that the condition (9.7) reads as $b_x(\rho , j) = |K_x|$ for all $\rho > 0$, $j \in \mathbb {R}^d$, and $x \in \mathcal {X}^Q$. Hence, if this condition holds, we obtain, since $\widetilde{m}(x) \in {{\,\mathrm{{\textsf{Rep}}}\,}}(0)$,

$$\begin{aligned} \partial _\varepsilon \big |_{\varepsilon = 0} F(m_\varepsilon , J^\star ) = - \frac{\mathfrak {m}'(\rho ) }{\mathfrak {m}^2(\rho )} |j|^2 \sum _{x \in \mathcal {X}^Q} \widetilde{m}(x) = 0. \end{aligned}$$

Adding the identities above, we conclude that $\frac{\textrm{d}}{\textrm{d}\varepsilon }\big |_{\varepsilon = 0} F(m_\varepsilon , J_\varepsilon ) = 0$ whenever (9.7) holds. Therefore, $(m^\star , J^\star )$ is a critical point of *F* in ${{\,\mathrm{{\textsf{Rep}}}\,}}(\rho , j)$. By convexity of *F*, it is a minimiser. Consequently, using Lemma 9.5, we obtain

$$\begin{aligned} f_\textrm{hom}(\rho , j) = F(m^\star , J^\star ) = \frac{1}{2 \mathfrak {m}(\rho )} \sum _{(x,y)\in \mathcal {E}^Q} d_{xy}{s_{xy}} (j \cdot n_{xy})^2 = \frac{|j|^2}{\mathfrak {m}(\rho )} = f(\rho , j), \end{aligned}$$

which is the desired identity.

To prove the converse, we assume that (9.7) does not hold, i.e., we have $b_{\bar{x}}(\rho , j) \ne |K_{\bar{x}}|$ for some $\rho > 0$, $j \in \mathbb {R}^d$, and $\bar{x} \in \mathcal {X}$. On the other hand, we claim that

$$\begin{aligned} \sum _{x \in \mathcal {X}^Q} b_x(\rho , j) = 1. \end{aligned}$$

To see this, observe first that, by definition of admissibility of $\mathfrak {m}_{xy}$ and the symmetry assumption $\mathfrak {m}_{xy}(a, b) = \mathfrak {m}_{yx}(b, a)$, we have

$$\begin{aligned} \mathfrak {m}'(\rho ) = \partial _\varepsilon \big |_{\varepsilon = 0} \mathfrak {m}(\rho + \varepsilon ) = \partial _\varepsilon \big |_{\varepsilon = 0} \mathfrak {m}_{xy}(\rho + \varepsilon ,\rho + \varepsilon )&= \partial _1 \mathfrak {m}_{xy}(\rho , \rho ) + \partial _2 \mathfrak {m}_{xy}(\rho , \rho ) \\ {}&= \partial _1 \mathfrak {m}_{xy}(\rho , \rho ) + \partial _1 \mathfrak {m}_{yx}(\rho , \rho ). \end{aligned}$$

Using this identity, the periodicity of *m* and *J*, and the identity (9.8) we obtain

$$\begin{aligned} \sum _{x \in \mathcal {X}^Q} b_x(\rho , j)&= \sum _{(x,y \in \mathcal {E}^Q} \frac{\partial _1 \mathfrak {m}_{xy}(\rho , \rho )}{\mathfrak {m}'(\rho )} d_{xy} s_{xy} \frac{(n_{xy} \cdot j)^2}{|j|^2} \\ {}&= \frac{1}{2} \sum _{(x,y \in \mathcal {E}^Q} \frac{\partial _1 \mathfrak {m}_{xy}(\rho , \rho ) + \partial _1 \mathfrak {m}_{yx}(\rho , \rho )}{\mathfrak {m}'(\rho )} d_{xy} s_{xy} \frac{(n_{xy} \cdot j)^2}{|j|^2} \\ {}&= \frac{1}{2} \sum _{(x,y \in \mathcal {E}^Q} d_{xy} s_{xy} \frac{(n_{xy} \cdot j)^2}{|j|^2} = 1, \end{aligned}$$

which proves the claim.

We thus infer that $b_x(\rho , j) / |K_x|$ is non-constant in *x*. (If it were, the identity $\sum _x |K_x| = 1 = \sum _x b_x(\rho , j)$ would imply that $b_x(\rho , j) = |K_x|$ for all *x*. But we assume that this doesn’t hold for $x = \bar{x}$.) Consequently, there exists a $\mathbb {Z}^d$-periodic function $\widetilde{m}: \mathcal {X}\rightarrow \mathbb {R}$ with $\sum _{x \in \mathcal {X}^Q} \widetilde{m}(x) = 0$ such that

$$\begin{aligned} \sum _{x \in \mathcal {X}^Q} b_x(\rho , j) \frac{\widetilde{m}(x)}{|K_x|} \ne 0. \end{aligned}$$

As before, we consider $(m^\star , J^\star ) \in {{\,\mathrm{{\textsf{Rep}}}\,}}(\rho , j)$ defined by (9.9). In view of (9.10), we infer that $(m^\star , J^\star )$ is *not* a critical point of *F* in ${{\,\mathrm{{\textsf{Rep}}}\,}}(\rho , j)$. As $(m^\star , J^\star )$ is a relatively interior point of ${{\,\mathrm{{\textsf{Rep}}}\,}}(\rho , j)$, it cannot be a minimiser, hence $f_\textrm{hom}(\rho ,j) < F(m^\star , J^\star ) = f(\rho , j)$.

*(ii)*: We construct an element of the subgradient $(p_m,p_J)\in \partial ^- F(m,J)$ with $\langle p_m, dm \rangle = \langle p_J, dJ \rangle = 0$ for all $dm\in {{\,\mathrm{{\textsf{Rep}}}\,}}(0)$, $dJ\in {{\,\mathrm{{\textsf{Rep}}}\,}}(0)$.

We set

$$\begin{aligned} p_m(x):= \sum _{y\sim x}\frac{J(x,y)^2}{|K_x|\mathfrak {m}^2(\rho )} \frac{d_{xy}}{s_{xy}}(p^{xy}_1 + p^{yx}_2) \end{aligned}$$

and check by a simple calculation involving the chosen supergradients $p^{xy}$ that $F(m+dm,J) - F(m,J) \ge \langle p_m, dm \rangle$ for all $dm\in \mathbb {R}^\mathcal {X}$ periodic. The isotropy condition (9.6) implies that $p_m$ is independent of *x* and thus $\langle p_m, dm \rangle = 0$ for all $dm\in {{\,\mathrm{{\textsf{Rep}}}\,}}(0)$.

Since *F* is differentiable in *J*, we have to choose $p_J:= \partial _J F(m,J)$. By the same calculation as in (3) we see that $\langle p_J, dJ \rangle = 0$ for all $dJ\in {{\,\mathrm{{\textsf{Rep}}}\,}}(0)$.

To see that (*m*, *J*) is indeed a local (and thus global) minimiser of *F* in ${{\,\mathrm{{\textsf{Rep}}}\,}}(\rho ,j)$, we introduce a parameter $\varepsilon >0$ and show that

9.11 $$\begin{aligned} \liminf _{\varepsilon \searrow 0} \frac{1}{\varepsilon }\left( F(m+\varepsilon dm, J+\varepsilon dJ) - F(m,J) \right) \ge 0 \end{aligned}$$

for all $dm\in {{\,\mathrm{{\textsf{Rep}}}\,}}(0)$ and $dJ\in {{\,\mathrm{{\textsf{Rep}}}\,}}(0)$.

To see this, we expand the difference

$$\begin{aligned}&\frac{1}{\varepsilon }\left( F(m+\varepsilon dm, J+\varepsilon dJ) - F(m,J) \right) \\ =&\frac{1}{\varepsilon }\left( F(m+\varepsilon dm, J+\varepsilon dJ) - F(m + \varepsilon dm,J) \right) + \underbrace{\frac{1}{\varepsilon }\left( F(m + \varepsilon dm, J) - F(m,J) \right) }_{\ge 0}\\ \ge&\langle \partial _J F(m,J) + o(1), dJ \rangle \rightarrow _{\varepsilon \rightarrow 0} 0, \end{aligned}$$

where we used that $(m,J) \mapsto \partial _J F(m,J)$ is continuous. Because *F* is convex, (9.11) implies that (*m*, *J*) is a minimiser of *F* in ${{\,\mathrm{{\textsf{Rep}}}\,}}(\rho ,j)$.

#### Remark 9.6

Given a concave mobility $\mathfrak {m}:\mathbb {R}_+ \rightarrow \mathbb {R}_+$, a popular admissible version is to take $\mathfrak {m}_{xy}(a,b):= \mathfrak {m}(\lambda _{xy} a + (1-\lambda _{xy}) b)$, with weights $\lambda _{xy}\in [0,1]$. If $\mathfrak {m}$ is differentiable, this means that $\partial _1 \mathfrak {m}_{xy}(\rho ,\rho ) = \lambda _{xy} \mathfrak {m}'(\rho )$. As a result, for certain finite-volume partitions we have to choose the weights $\lambda _{xy}$ to satisfy (9.7).

Of particular importance is the $\mathbb {W}_2$ case $\mathfrak {m}(\rho ) = \rho$, which was treated in [26] and [25]. Here an admissible version $\mathfrak {m}_{xy}$ is called an admissible mean. For differentiable $\mathfrak {m}_{xy}$, condition (9.7) reduces to

$$\begin{aligned} \sum _{y\sim x} \partial _1 \mathfrak {m}(\rho ,\rho ) d_{xy}s_{xy} n_{xy} \otimes n_{xy} = |K_x| {{\,\textrm{id}\,}}. \end{aligned}$$

We note that condition (9.7) cannot be satisfied for a large class of finite-volume partitions, although the square partition fulfills it with $\partial _1 \mathfrak {m}(\rho ,\rho ) = 1/2$. The condition also holds for some other partitions that are not $\mathbb {Z}^d$-periodic, such as the equilateral triangular and hexagonal partitions; see [26].

If we allow ourselves to use nonsmooth admissible versions of $\mathfrak {m}$, it makes sense to use $\mathfrak {m}_{xy}(a,b):= \mathfrak {m}(\min (a,b))$, as this choice guarantees the largest possible supergradient $\partial ^+ \mathfrak {m}_{xy} = \partial ^+ \mathfrak {m}\{(\lambda , 1-\lambda )\,:\,\lambda \in [0,1] \}$ along the diagonal, making it more likely that $f_\textrm{hom}(\rho ,j) = \frac{|j|_2^2}{\mathfrak {m}(\rho )}$.

**Fig. 6:**
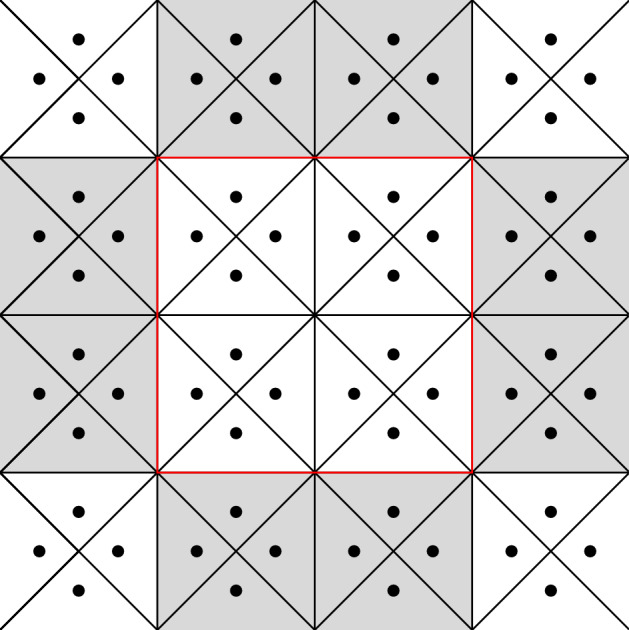
A $\mathbb {Z}^2$-periodic finite-volume partition of $\mathbb {R}^2$. The unit cube $[0,1]^2 \subseteq \mathbb {R}^2$ is shown in red (color figure online)

#### Example 9.7

Let us consider the triangulation given in Fig. 6, where each unit square consists of four triangles: north, south, west, and east. We now show that (9.7) cannot be satisfied here, but (9.6) is satisfied for the particular nonsmooth choice $\mathfrak {m}_{xy}(\rho _1,\rho _2)=\min (\rho _1,\rho _2)$.

For the smooth case we assume that $\mathfrak {m}_{xy}(\rho ,\rho )=\rho$ and define $\lambda _{xy}=\partial _1\mathfrak {m}_{xy}$ and $\lambda _{yx}=\partial _2\mathfrak {m}_{xy}$. Note that by the chain rule $\lambda _{xy}+\lambda _{yx}=1$. Let

$$\begin{aligned} A_x:=\sum _{y\sim x} \lambda _{xy} d_{xy}s_{xy}n_{xy}\otimes n_{xy}. \end{aligned}$$

For $x_N$ in the north triangle and $x_S$ in the south triangle we obtain that

$$\begin{aligned} e_2\cdot (A_{N}+A_{S})e_2=\frac{1}{2}+\frac{1}{8}(\lambda _{SE}+\lambda _{NE}+\lambda _{SW}+\lambda _{NW}) \end{aligned}$$

since $d_{NW}s_{NW}=\frac{1}{4}$, $d_{NS}s_{NS}=\frac{1}{2}$, $n_{NS}=e_2$ and $n_{NE}=(\frac{1}{\sqrt{2}},-\frac{1}{\sqrt{2}})^T$ Similarly we obtain for $x_W$ in the west and $x_E$ in the east triangle

$$\begin{aligned} e_1\cdot (A_{W}+A_{E})e_1=\frac{1}{2}+\frac{1}{8}(\lambda _{ES}+\lambda _{EN}+\lambda _{WS}+\lambda _{WN}). \end{aligned}$$

Inserting the last two equalities into (9.7) we find that $e_2\cdot A_xe_2 = e_1 \cdot A_x e_1 = \frac{1}{4}$ for all $x\in \{S,E,N,W\}$, i.e. that

$$\begin{aligned} \lambda _{SE}+\lambda _{NE}+\lambda _{SW}+\lambda _{NW}=\lambda _{ES}+\lambda _{EN}+\lambda _{WS}+\lambda _{WN}=0. \end{aligned}$$

But this is a contradiction to $\lambda _{xy}+\lambda _{yx}=1$. In particular there exists no $\mathfrak {m}_{xy}$ satisfying (9.7).

For the nonsmooth case note that the supergradient for $\mathfrak {m}_{xy}(\rho _1,\rho _2)=\min (\rho _1,\rho _2)$ is given by

$$\begin{aligned} \partial ^+\mathfrak {m}_{xy}(\rho ,\rho )=\{(\lambda ,1-\lambda ) : \lambda \in [0,1]\}. \end{aligned}$$

For $\rho \in \mathbb R_+$ and $j\in \mathbb R^d$ we set

$$\begin{aligned} p^{NS}=p^{SN}=p^{EW}=p^{WE}=&\left( \frac{1}{2},\frac{1}{2}\right) \in \partial ^+\mathfrak {m}_{xy}(\rho ,\rho )\\ p^{NE}=p^{NW}=p^{SE}=p^{SW}=&\left( \frac{j_1^2}{|j|_2^2},\frac{j_2^2}{|j|^2_2}\right) \in \partial ^+\mathfrak {m}_{xy}(\rho ,\rho )\\ p^{EN}=p^{WN}=p^{ES}=p^{WS}=&\left( \frac{j_2^2}{|j|_2^2},\frac{j_1^2}{|j|^2_2}\right) \in \partial ^+\mathfrak {m}_{xy}(\rho ,\rho ). \end{aligned}$$

We need to show that $a_{x,j}:=\frac{1}{|K_x|}\sum _{y\sim x}(p_1^{xy}+p_2^{yx})d_{xy}s_{xy}(n_{xy}\cdot j)^2$ is independent of *x*. For *x* in the north or the south triangle we find

$$\begin{aligned} a_{S,j}= a_{N,j}=&4\left( \frac{1}{2} j_2^2+\frac{2}{8} \frac{2j_1^2}{|j|_2^2}\left( \frac{(j_1-j_2)^2}{2}+\frac{(j_1+j_2)^2}{2}\right) \right) \\ =&4\left( \frac{1}{2} j_2^2+\frac{1}{2} \frac{j_1^2}{|j|_2^2}|j|_2^2\right) =2|j|_2^2. \end{aligned}$$

Similarly for x in the west or east triangle we obtain

$$\begin{aligned} a_{E,j}=a_{W,j} =&4\left( \frac{1}{2} j_1^2+\frac{2}{8} \frac{2j_2^2}{|j|_2^2}\left( \frac{(j_2-j_1)^2}{2}+\frac{(j_1+j_2)^2}{2}\right) \right) \\ =&4\left( \frac{1}{2} j_1^2+\frac{1}{2} \frac{j_2^2}{|j|_2^2}|j|_2^2\right) =2|j|_2^2. \end{aligned}$$

Consequently, this is independent of *x* and (9.6) holds.

## Data Availability

Data sharing not applicable to this article as no datasets were generated or analysed during the current study.

## Appendix A: The Kantorovich–Rubinstein metric on signed measures

We collect some facts on the Kantorovich–Rubinstein metric that are used in the paper. We refer to [8, Section 8.10(viii)] for more details.

Let (*X*, *d*) be a metric space. Let $\mathcal {M}(X)$ denote the space of finite signed Borel measures on *X*. For $\mu \in \mathcal {M}(X)$, let $\mu ^+, \mu ^- \in \mathcal {M}_+(X)$ be the positive and negative parts, respectively. Let $|\mu | = \mu ^+ + \mu ^-$ be its variation, and $\Vert \mu \Vert _\textrm{TV}:= |\mu |(X)$ be its total variation.

### Definition A.1

(Weak and vague convergence) Let $\mu , \mu _n \in \mathcal {M}(X)$ for $n = 1, 2, \ldots$.

(i) We say that $\mu _n \rightarrow \mu$ weakly in $\mathcal {M}(X)$ if $\int _{X} \psi \, \textrm{d}\mu _n \rightarrow \int _{X} \psi \, \textrm{d}\mu$ for every $\psi \in \mathcal {C}_\textrm{b}(X)$.
(ii) We say that $\mu _n \rightarrow \mu$ vaguely in $\mathcal {M}(X)$ if $\int _{X} \psi \, \textrm{d}\mu _n \rightarrow \int _{X} \psi \, \textrm{d}\mu$ for every $\psi \in \mathcal {C}_\textrm{c}(X)$.

If (*X*, *d*) is compact, $\mathcal {M}(X)$ is a Banach space endowed with the norm $\Vert \mu \Vert _\textrm{TV}$. By the Riesz-Markov theorem, it is the dual space of the Banach space $\mathcal {C}(X)$ of all continuous functions $\psi : X \rightarrow \mathbb {R}$ endowed with the supremum norm $\Vert \psi \Vert _\infty = \sup _{x \in X} |\psi (x)|$.

For $\psi : X \rightarrow \mathbb {R}$ let $\textrm{Lip}(\psi ):= \sup _{x \ne y} \frac{|\psi (x) - \psi (y)|}{d(x,y)}$ be its Lipschitz constant.

### Definition A.2

Let (*X*, *d*) be a compact metric space. The *Kantorovich–Rubinstein norm* on $\mathcal {M}(X)$ is defined by

A.12 $$\begin{aligned} \Vert \mu \Vert _{\textrm{KR}(X)} := \sup \bigg \{ \int _X \psi \, \textrm{d}\mu \ : \ \psi \in \mathcal {C}(X), \ \Vert \psi \Vert _\infty \le 1, \ \textrm{Lip}(\psi ) \le 1 \bigg \}. \end{aligned}$$

In non-trivial situations (i.e., when *X* contains an infinite convergent sequence), the norms $\Vert \cdot \Vert _\textrm{KR}$ and $\Vert \cdot \Vert _\textrm{TV}$ are not equivalent. Thus, by the open mapping theorem, $(\mathcal {M}(X), \Vert \cdot \Vert _\textrm{KR})$ is not a complete space.

A closely related norm on $\mathcal {M}(X)$ that is often considered is

$$\begin{aligned} \Vert \mu \Vert _{\widetilde{\textrm{KR}}(X)} := |\mu (X)| + \sup \bigg \{ \int _{X} \psi \, \textrm{d}\mu \ : \ \psi \in \mathcal {C}(X), \ \psi (x_0) = 0, \ \textrm{Lip}(\psi ) \le 1 \bigg \}, \end{aligned}$$

for some fixed $x_0 \in X$; see [8, Section 8.10(viii)]. The next result shows that these norms are equivalent.

### Proposition A.3

Let (*X*, *d*) be a compact metric space. For $\mu \in \mathcal {M}(X)$ we have

$$\begin{aligned} \Vert \mu \Vert _{\textrm{KR}(X)} \le \Vert \mu \Vert _{\widetilde{\textrm{KR}}(X)} \le c_X \Vert \mu \Vert _{\textrm{KR}(X)}, \end{aligned}$$

where $c_X < \infty$ depends only on ${{\,\textrm{diam}\,}}(X)$.

### Proof

We start with the first inequality. Let $\psi \in \mathcal {C}(X)$ with $\Vert \psi \Vert _\infty \le 1$ and $\textrm{Lip}(\psi ) \le 1$. Define $\varphi := \psi - \psi (x_0)$, so that $\varphi (x_0) = 0$ and $\textrm{Lip}(\varphi ) = \textrm{Lip}(\psi ) \le 1$. Then

$$\begin{aligned} \int \psi \, \textrm{d}\mu = \int \psi (x_0) + \varphi \, \textrm{d}\mu = \psi (x_0) \mu (X) + \int \varphi \, \textrm{d}\mu \le |\mu (X)| + \int \varphi \, \textrm{d}\mu \le \Vert \mu \Vert _{\widetilde{\textrm{KR}}}. \end{aligned}$$

Taking the supremum over $\psi$ yields the desired bound.

Let us now prove the second inequality. Set $\Delta := 1 \vee {{\,\textrm{diam}\,}}(X)$. Take $\psi \in \mathcal {C}(X)$ with $\psi (x_0) = 0$ and $\textrm{Lip}(\psi ) \le 1$. Then $|\psi (x)| = |\psi (x) - \psi (x_0)| \le d(x,x_0) \le {{\,\textrm{diam}\,}}(X) \le \Delta$ for all $x \in X$, so that $\Vert \frac{\psi }{\Delta }\Vert _\infty \le 1$ and $\textrm{Lip}(\frac{\psi }{\Delta }) \le 1$. We obtain

$$\begin{aligned} \int \psi \, \textrm{d}\mu = \Delta \int \frac{\psi }{\Delta } \, \textrm{d}\mu \le \Delta \Vert \mu \Vert _{\textrm{KR}}. \end{aligned}$$

Moreover, $|\mu (X)| \le \Vert \mu \Vert _{\textrm{KR}}$ as can be seen by taking $\psi = \pm 1$ in (A.12) It follows that

$$\begin{aligned} \Vert \mu \Vert _{\widetilde{\textrm{KR}}} \le (1 + \Delta ) \Vert \mu \Vert _{\textrm{KR}}, \end{aligned}$$

as desired.

### Proposition A.4

(Relation to $\mathbb {W}_1$) Let (*X*, *d*) be a compact metric space. If $\mu _1, \mu _2 \in \mathcal {M}_+(X)$ are nonnegative measures of equal total mass, we have $\Vert \mu _1 - \mu _2 \Vert _{\widetilde{\textrm{KR}}} = \mathbb {W}_1(\mu _1, \mu _2)$.

### Proof

This follows from the Kantorovich duality for the distance $\mathbb {W}_1$.

On the subset of *nonnegative* measures, the $\textrm{KR}$-norm induces the weak$^*$ topology:

### Proposition A.5

(Relation to weak$^*$-convergence) Let (*X*, *d*) be a compact metric space. For $\mu _n, \mu \in \mathcal {M}_+(X)$ we have

$$\begin{aligned} \mu _n \rightarrow \mu \, \text {weakly} \quad \text {if and only if} \quad \Vert \mu _n - \mu \Vert _{\textrm{KR}} \rightarrow 0. \end{aligned}$$

### Proof

See [8, Theorem 8.3.2].

### Remark A.6

(Testing against smooth functions) If $X = \mathbb {T}^d$, the space of $\mathcal {C}^1$ functions $\psi$ with $\textrm{Lip}(\psi ) \le 1$ is dense in the set of Lipschitz functions with $\textrm{Lip}(\psi ) \le 1$; see, e.g., [40, Proposition A.5]. Consequently,

A.13 $$\begin{aligned} \Vert \mu \Vert _{\textrm{KR}(X)} = \sup \bigg \{ \int _X \psi \, \textrm{d}\mu \ : \ \psi \in \mathcal {C}^1(\mathbb {T}^d), \ \Vert \psi \Vert _\infty \le 1, \ \Vert \nabla \psi \Vert _\infty \le 1 \bigg \}. \end{aligned}$$

### Remark A.7

The identity (A.13) shows that $\Vert \cdot \Vert _{\textrm{KR}}$ is the dual norm of the separable Banach space $\mathcal {C}^1(Q)$. The dual space of $\mathcal {C}^1(Q)$ is a strict superset of the finite Borel measures.

## Appendix B: Norms on curves in the space of measures

We work with curves of bounded variation taking values in the space $\mathcal {M}_+(\mathbb {T}^d)$.

### Definition B.1

(Curves of bounded variation) The space $\textrm{BV}_{\textrm{KR}}(\mathcal {I}; \mathcal {M}_+(\mathbb {T}^d))$ consists of all curves of measures ${\varvec{\mu }}: \mathcal {I}\rightarrow \mathcal {M}_+(\mathbb {T}^d)$ such that the $\textrm{BV}$-seminorm

B.14 $$\begin{aligned} \Vert {\varvec{\mu }}\Vert _{\textrm{BV}_{\textrm{KR}}(\mathcal {I}; \mathcal {M}_+(\mathbb {T}^d))} := \sup \left\{ \int _\mathcal {I}\int _{\mathbb {T}^d} \partial _t \varphi _t \, \textrm{d}\mu _t \, \textrm{d}t \ : \ \varphi \in \mathcal {C}_c^1\big (\mathcal {I}; \mathcal {C}^1(\mathbb {T}^d)\big ), \ \max _{t\in \mathcal {I}} \Vert \varphi \Vert _{\mathcal {C}^1(\mathbb {T}^d)} \le 1 \right\} \end{aligned}$$

is finite.

### Remark B.2

The space $\textrm{BV}_{\textrm{KR}}(\mathcal {I}; \mathcal {M}_+(\mathbb {T}^d))$ is a (non-closed) subset of the space $\textrm{BV}(\mathcal {I}; X^*)$, where *X* is the separable Banach space $\mathcal {C}^1(\mathbb {T}^d)$. We refer to [28, Section 2] for the equivalence of several definitions of $\textrm{BV}\big (\mathcal {I}; X^* \big )$.

### Definition B.3

The space $W^{1,1}_\textrm{KR}(\mathcal {I}; \mathcal {M}_+(\mathbb {T}^d))$ consists of all curves $(\mu _t)_{t \in \mathcal {I}}$ in the Banach space-valued Sobolev space $W^{1,1}\big (\mathcal {I}; (\mathcal {C}^1(\mathbb {T}^d))^*\big )$ such that $\mu _t \in \mathcal {M}_+(\mathbb {T}^d)$ for a.e. $t \in \mathcal {I}$.

## Appendix C: Domain property of convex functions

### Lemma C.1

(Domain properties of convex functions) Let $f: \mathbb {R}^n \rightarrow \mathbb {R}\cup \{ + \infty \}$ be convex, and let $x^\circ \in {{\,\mathrm{\textsf{D}}\,}}(f)^\circ$. For every $\lambda \in (0, 1)$ and every bounded set $K \subseteq {{\,\mathrm{\textsf{D}}\,}}(f)$, there exists a compact convex set $K_{\lambda } \subseteq {{\,\mathrm{\textsf{D}}\,}}(f)^\circ$ such that

$$\begin{aligned} (1-\lambda ) K + \lambda x^\circ \subseteq K_{\lambda }. \end{aligned}$$

### Proof

Let $K \subseteq {{\,\mathrm{\textsf{D}}\,}}(f)$ be bounded and $\lambda \in (0,1)$. Since $x^\circ \in {{\,\mathrm{\textsf{D}}\,}}(f)^\circ$, we can pick $r > 0$ such that $B(x^\circ , r) \subseteq {{\,\mathrm{\textsf{D}}\,}}(f)^\circ$. Fix $y \in \bar{K}$ and set $y_\lambda := (1-\lambda ) y + \lambda x^\circ$. We claim that $B( y_\lambda , \lambda r) \subseteq {{\,\mathrm{\textsf{D}}\,}}(f)^\circ$.

To prove the claim, it suffices to show that $B( y_\lambda , \lambda r) \subseteq {{\,\mathrm{\textsf{D}}\,}}(f)$, since $B( y_\lambda , \lambda r)$ is open. Take $z \in B( y_\lambda , \lambda r)$ and pick a sequence $(y_n)_n \subset K$ such that $y_n \rightarrow y$. Observe that $z = (1-\lambda ) y_n + \lambda \widetilde{x}_n$ with $\widetilde{x}_n \in B(x^\circ , r)$ if *n* is large enough (indeed, $\widetilde{x}_n - x^\circ = \frac{1}{\lambda }(z - y_\lambda )+ \frac{1-\lambda }{\lambda }(y-y_n)$ and $|z - y_\lambda | < \lambda r$ ). Since $y_n, \widetilde{x}_n \in {{\,\mathrm{\textsf{D}}\,}}(f)$, the claim follows by convexity of *f*.

We now define

$$\begin{aligned} C_{\lambda } := \bigcup _{y \in K} B\Big (y_\lambda , \frac{\lambda r}{3} \Big ) \quad \text { and }\quad K_\lambda := \mathop {\textrm{Conv}}\limits ( \overline{ C_\lambda } ). \end{aligned}$$

By construction, $K_{\lambda }$ is convex, bounded, and closed, thus compact. Let us show that $K_{\lambda } \subseteq {{\,\mathrm{\textsf{D}}\,}}(f)^\circ$.

By convexity of *f*, it suffices to show that $\overline{C_\lambda } \subseteq {{\,\mathrm{\textsf{D}}\,}}(f)^\circ$. Pick $z \in \overline{C_\lambda }$ and $\{z_n\}_n \subseteq C_\lambda$ such that $z_n \rightarrow z$. Then there exists $y_n \in K$ such that $z_n \in B\big ( (y_n)_\lambda , \frac{\lambda r}{3} \big )$. Passing to a subsequence, we may assume that $y_n \rightarrow \bar{y}$ for some $\bar{y} \in \bar{K}$ and $z_n \in B\big ( {\bar{y}}_\lambda , \frac{\lambda r}{2} \big )$ for $n \ge \bar{n} \in \mathbb {N}$. Taking the limit as $n \rightarrow +\infty$ we infer that $z \in \overline{ B\big ( {\bar{y}}_\lambda , \frac{\lambda r}{2} \big )}$. Since $B\big ( {\bar{y}}_\lambda , \lambda r \big ) \subseteq {{\,\mathrm{\textsf{D}}\,}}(f)^\circ$, it follows that $z \in {{\,\mathrm{\textsf{D}}\,}}(f)^\circ$.

## Appendix D: Notation

For the convenience of the reader we collect some notation used in this paper.

**Table:**

$A^\circ$	Topological interior of a set *A*.
${{\,\mathrm{\textsf{D}}\,}}(F)$	The domain ${{\,\mathrm{\textsf{D}}\,}}(F) = \{x \in X : F(x) < \infty \}$ of $F :X \rightarrow \mathbb {R}\cup \{ + \infty \}$.
$\mathcal {I}$	Bounded open time interval.
$\mathcal {M}^d(A)$	The space of finite $\mathbb {R}^d$-valued Radon measures on *A*.
$\mathcal {M}_+(A)$	The space of finite (positive) Radon measures on *A*.
$\mathbb {R}_a^\mathcal {E}$	Anti-symmetric vector fields on $\mathcal {E}$: $\mathbb {R}_a^\mathcal {E}=\{ J \in \mathbb {R}^\mathcal {E}\ : \ J(x,y) = J(y,x) \}$.
$\mathcal {X}^Q$	The set of all $x \in \mathcal {X}$ with $x_\textsf{z}= 0$.
$\mathcal {E}^Q$	The set of all $(x,y) \in \mathcal {E}$ with $x_\textsf{z}= 0$.
$\mathbb {R}_a^\mathcal {E}$	The set of anti-symmetric real functions on $\mathcal {E}$.
$\mathbb {T}_\varepsilon ^d$, $\mathbb {Z}_\varepsilon ^d$	The discrete torus of mesh size $\varepsilon >0$: $\mathbb {T}_\varepsilon ^d = ( \varepsilon \mathbb {Z}/\mathbb {Z})^d = \varepsilon \mathbb {Z}_\varepsilon ^d$.
${{\,\mathrm{\textsf{Eff}}\,}}(J)$	The effective flux of *J*: ${{\,\mathrm{\textsf{Eff}}\,}}(J) = \frac{1}{2} \sum _{(x,y) \in \mathcal {E}^Q} J(x,y) (y_\textsf{z}- x_\textsf{z})$.
${{\,\mathrm{{\textsf{Rep}}}\,}}(\rho )$	The set of representatives of $\rho \in \mathbb {R}_+$, i.e, all $m \in \mathbb {R}_+^\mathcal {X}$ s.t. $\sum _{x \in \mathcal {X}^Q} m(x) =\rho$.
${{\,\mathrm{{\textsf{Rep}}}\,}}(j)$	The set of representatives of $j \in \mathbb {R}^d$, i.e, all $J \in \mathbb {R}_a^\mathcal {E}$ divergence-free and s.t.
	$$\frac{1}{2}\sum _{(x,y) \in \mathcal {X}^Q} J(x,y) (y_\textsf{z}- x_\textsf{z}) = j$$.

**Table:**

${{\,\mathrm{{\textsf{Rep}}}\,}}(\rho ,j)$	The set of representatives of $\rho \in \mathbb {R}_+$, $j \in \mathbb {R}^d$: ${{\,\mathrm{{\textsf{Rep}}}\,}}(\rho ,j) = {{\,\mathrm{{\textsf{Rep}}}\,}}(\rho ) \times {{\,\mathrm{{\textsf{Rep}}}\,}}(j)$.
$Q_\varepsilon ^z$	The cube of size $\varepsilon >0$ centered in $\varepsilon z \in \mathbb {T}^d$: for $z \in \mathbb {Z}_\varepsilon ^d$, $Q_\varepsilon ^z := [0,\varepsilon )^d + \varepsilon z$.
$S^{\bar{z}}$	Shift operator: $S_\varepsilon ^{\bar{z}} : \mathcal {X}\rightarrow \mathcal {X}$, $S_\varepsilon ^{\bar{z}}(x) = (\bar{z} + z, v)$ for $x = (z,v) \in \mathcal {X}$.
	Shift operator: $S_\varepsilon ^{\bar{z}}: \mathcal {E}\rightarrow \mathcal {E}$, $S_\varepsilon ^{\bar{z}}(x,y) := \big ( S_\varepsilon ^{\bar{z}}(x), S_\varepsilon ^{\bar{z}}(y) \big )$ for $(x,y) \in \mathcal {E}_\varepsilon$
$\sigma ^z$	$\sigma _\varepsilon ^{\bar{z}} \psi : \mathcal {X}_\varepsilon \rightarrow \mathbb {R}, \quad (\sigma _\varepsilon ^{\bar{z}} \psi )(x) := \psi (S_\varepsilon ^{\bar{z}}(x)) \quad \text {for} \; x \in \mathcal {X}_\varepsilon .$
	$$\sigma _\varepsilon ^{\bar{z}}J: \mathcal {E}_\varepsilon \rightarrow \mathbb {R}, \quad (\sigma _\varepsilon ^{\bar{z}} J)(x,y) := J(S_\varepsilon ^{\bar{z}}(x,y)) \quad \text {for} \; (x,y) \in \mathcal {E}_\varepsilon . $$
$T_\varepsilon ^{\bar{z}}$	Rescaling operator: $T_\varepsilon ^{\bar{z}} : \mathcal {X}\rightarrow \mathcal {X}_\varepsilon$: $T_\varepsilon ^{\bar{z}}(x) = (\varepsilon (\bar{z} + z), v)$ for $x = (z,v) \in \mathcal {X}$.
$\tau _\varepsilon ^z$	$\tau _\varepsilon ^{\bar{z}} \psi : \mathcal {X}\rightarrow \mathbb {R}, \quad \big (\tau _\varepsilon ^{\bar{z}} \psi \big )(x) := \psi \big ( T_\varepsilon ^{\bar{z}}(x) \big ) \quad \text { for } x \in \mathcal {X}$.
	$$\tau _\varepsilon ^{\bar{z}} J : \mathcal {E}\rightarrow \mathbb {R}, \quad \big ( \tau _\varepsilon ^{\bar{z}} J \big )(x,y) := J \big ( T_\varepsilon ^{\bar{z}}(x), T_\varepsilon ^{\bar{z}}(y) \big ) \quad \text { for } (x,y) \in \mathcal {E}$$.
$\mathcal{C}\mathcal{E}$	Discrete continuity equation: $({\pmb {m}},\pmb {J}) \in \mathcal{C}\mathcal{E}$ iff $\partial _t m_t + {{\,\mathrm{\text {\textsf{div}}}\,}}J = 0$ on $(\mathcal {X},\mathcal {E})$.
$\mathbb{C}\mathbb{E}$	Continuous continuity equation: $({\varvec{\mu }},{\varvec{\nu }}) \in \mathbb{C}\mathbb{E}$ iff $\partial _t \mu _t + \nabla \cdot {\varvec{\nu }}= 0$ on $\mathbb {T}^d$.
$\textrm{BV}$	More precisely $\textrm{BV}_{\textrm{KR}}(\mathcal {I}; \mathcal {M}_+(\mathbb {T}^d))$: the space of time-dependent curves of
	(Positive) measures with bounded variation with respect to the $\textrm{KR}$ norm
	(Kantorovich–Rubenstein) on $\mathcal {M}_+(\mathbb {T}^d)$.
$W^{1.1}$	More precisely $W^{1,1}_\textrm{KR}(\mathcal {I}; \mathcal {M}_+(\mathbb {T}^d))$: the space of time-dependent curves of
	(Positive) measures belonging to the Banach space $W^{1,1}\big (\mathcal {I}; (\mathcal {C}^1(\mathbb {T}^d))^*\big )$.
$\textrm{P}_\varepsilon \mu , \textrm{P}_\varepsilon \nu$	Discretisation of $\mu \in \mathcal {M}_+(\mathbb {T}^d)$, $\nu \in \mathcal {M}^d(\mathbb {T}^d)$: for $z \in \mathbb {Z}_\varepsilon ^d$, ($\textrm{P}_\varepsilon \mu (z), \textrm{P}_\varepsilon \nu (z)) \in$
	$\mathbb {R}_+ \times \mathbb {R}^d$, given by $\textrm{P}_\varepsilon \mu (z) = \mu (Q_\varepsilon ^z)$, $\textrm{P}_\varepsilon \nu (z) = \big ( (\nu \cdot e_i)(\partial Q_\varepsilon ^z\cap \partial Q_\varepsilon ^{z+ e_i}) \big )_i$.

In the paper we use some standard terminology from graph theory. Let $(\mathcal {X}, \mathcal {E})$ be a locally finite graph.

A *discrete vector field* is an anti-symmetric function $J: \mathcal {E}\rightarrow \mathbb {R}$.

Its *discrete divergence* is the function ${{\,\mathrm{\text {\textsf{div}}}\,}}J: \mathcal {X}\rightarrow \mathbb {R}$ defined by

B.15 $$\begin{aligned} {{\,\mathrm{\text {\textsf{div}}}\,}}J(x) := \sum _{y \sim x} J(x,y). \end{aligned}$$

We say that *J* is *divergence-free* if ${{\,\mathrm{\text {\textsf{div}}}\,}}J = 0$.

## Footnotes

1 In the sequel we consider more general discrete energy densities *F*(*m*, *J*), not necessarily sums of edge-energies.

2 We regard measures on $\mathcal {I}_n \times \mathbb {T}^d$ as measures on the bigger set $\mathcal {I}\times \mathbb {T}^d$ by the canonical inclusion.

3 See also Sect. 9.2.

4 To be precise, this is an application of these lemmas to the case of $V:= \{v\}$, thus $\mathcal {X}_\varepsilon \simeq \mathbb {Z}_\varepsilon ^d$.

5 As before, it’s an application of these lemmas on $\mathbb {Z}_\varepsilon ^d$ (corresponding to $V= \{v\}$).

6 Concavity of $\mathfrak {m}_{xy}$ is not necessary for convexity of *F*. If $\mathfrak {m}_{xy}$ is not concave, a local version of the supergradient can be substituted into (9.6). For readability we restrict ourselves to the concave case.
